# Analysis of the International, Regional, and National Endocarditis-Related Disease Burdens (1990–2021), and Changes to Projections for the Next 15 Years: A Population-Based Study

**DOI:** 10.31083/RCM27168

**Published:** 2025-05-20

**Authors:** Qiyuan Bai, Hao Chen, Hongxu Liu, Xuhua Li, Yang Chen, Dan Guo, Bing Song, Cuntao Yu

**Affiliations:** ^1^The First Clinical Medical College of Lanzhou University, 730000 Lanzhou, Gansu, China; ^2^Department of Cardiovascular Surgery, First Hospital of Lanzhou University, 730013 Lanzhou, Gansu, China; ^3^Department of Cardiovascular Surgery, Fuwai Hospital, National Center for Cardiovascular Diseases, Chinese Academy of Medical Sciences, Peking Union Medical College, 100006 Beijing, China

**Keywords:** endocarditis, incidence, prevalence, deaths, disability-adjusted life-years, disease burden, GBD

## Abstract

**Background:**

Endocarditis can lead to health loss and even death, making it one of the major contributors to the global disease burden, with its incidence continuously increasing. This study aimed to assess the trends and frontier analysis of the worldwide burden of endocarditis over the past 30 years and to improve the predictions of its future burden by 2035.

**Methods:**

We analyzed the trends of global endocarditis incidence, prevalence, deaths, and disability-adjusted life years (DALYs) at international, regional, and national levels from 1990 to 2021 using a comprehensive, localized, and multidimensional approach. Clustering analysis assessed the changing patterns of disease burden related to endocarditis in the Global Burden of Disease (GBD) study regions. Correlation analysis was conducted to determine the potential relationships between the burden of endocarditis and the socio-demographic index (SDI) and the Human Development Index (HDI). Frontier analysis was performed to identify possible areas for improvement and the disparities in development status among countries. Additionally, we projected the changes in the burden of endocarditis by 2035.

**Results:**

From a global perspective, between 1990 and 2021, the incidence, prevalence, mortality, and DALYs associated with endocarditis have shown a continuous upward trend. At the national level, significant differences were observed in the incidence, prevalence, mortality, and DALYs of endocarditis worldwide. The United States had the highest number of deaths; India had the highest number of DALYs; Thailand had the highest incidence; Sri Lanka had the highest prevalence. The age-standardized rates (ASRs) for endocarditis prevalence, incidence, mortality, and DALYs increased steadily with age, peaking in the 95-year-old and above age group. The incidence, prevalence, mortality, and DALYs for males were 1.27 times, 1.02 times, 1.06 times, and 1.37 times those of females, respectively. Clustering analysis results indicated a significant increase in the estimated annual percentage change (EAPC) of mortality and DALY rates for endocarditis in East Asia. A significant correlation exists between EAPC and the ASRs of disease burden. Frontier analysis showed that countries and regions with higher SDIs have greater potential for improving the disease burden. The Bayesian age–period–cohort (BAPC) results indicated that the incidence, prevalence, mortality, and DALYs case numbers are expected to increase, with the ASRs for incidence and prevalence also projected to show a continuous upward trend by 2035.

**Conclusions:**

The global burden of endocarditis, a significant public health issue, has shown an overall upward trend from 1990 to 2021. The continuous increase in the prevalence and incidence of endocarditis, driven by population growth and aging, has become a major challenge for its control and management, which may guide better public health policy formulation and the rational allocation of medical resources. This targeted approach is crucial for effectively alleviating the burden of this disease.

## 1. Introduction

Endocarditis occurs when bacterial or fungal pathogens enter the bloodstream and attach to the inner layer of the heart (the endocardium), usually affecting the heart valves [1, 2, 3]. Endocarditis was first described over 350 years ago and involves the infection of the surface of the heart’s endocardium. The clinical manifestations of infective endocarditis can affect various organ systems, with cardiac symptoms including valve vegetations, abscesses, extension of infection to surrounding tissues, and myocarditis or pericarditis [4, 5]. In the general population, it affects 3 to 10 people per 100,000 people each year, and epidemiological studies indicate that the incidence is on the rise [3, 6]. Even in high-income countries, providing a high level of coordinated care within healthcare systems remains challenging, while low-income countries often face even more dire circumstances [4]. In the United States, there are 40,000 to 50,000 new cases annually, with average hospitalization costs exceeding $120,000 per patient [7]. The trend of infective endocarditis in California and New York from 1998 to 2013 shows that infective endocarditis has long been a clinically challenging disease to treat. The incidence of endocarditis continues to rise, with reported cases in the United States increasing by 35% from 2000 to 2013 [6, 8]. Therefore, despite the increasing availability of antimicrobial therapies and improvements in surgical techniques, endocarditis remains a highly morbid disease, with a total mortality rate of 20% within 30 days [9, 10].

Clinical medical risk factors leading to endocarditis are similar to the main risk factors for infection. Contact with healthcare systems itself may be a risk factor: a cohort study on endocarditis found that 25% of affected patients had a recent healthcare exposure [11]. In addition to non-cardiac diseases and high-risk behaviors that may increase the risk of endocarditis in patients, there has also been an increase in the number of patients at risk due to the materials used in prosthetic heart valves [12]. Moreover, an international cohort study indicated that a longer follow-up period after hospitalization for endocarditis was associated with higher mortality rates among endocarditis patients. In this cohort, the in-hospital mortality rate was between 15% and 20%, while the one-year mortality rate approached 40% [11]. In another study targeting older adults aged 65 and older, the one-year mortality rate for those under 65 was found to be 13%. In contrast, the one-year mortality rate for older adults aged 65 and over was higher, at 37.3% [13]. For over forty years, chronic hemodialysis has been recognized as a risk factor for infectious complications such as bacteremia and infective endocarditis, with infective endocarditis being a common and severe complication among chronic hemodialysis patients [14, 15, 16]. It has been reported that the incidence of endocarditis can be as high as 6% in patients undergoing chronic hemodialysis [17, 18, 19]. Studies indicate that the incidence of endocarditis in hemodialysis patients may be significantly higher than previously anticipated, contributing to the high mortality rate in this population [20].

The aim of this study is to investigate the epidemiological indicators and burden of endocarditis in the 21 countries within the National Ageing Monitoring and Evaluation (NAME) from 1990 to 2021, categorized by national socio-demographic index (SDI), age groups, and sex. Data is updated from epidemiological studies on endocarditis published in the Global Burden of Disease (GBD) database, summarizing and calculating the estimated annual percentage change (EAPC) of global prevalence and mortality rates, as well as exploring temporal trends and geographic variations. Additionally, we conducted a decomposition analysis of the incidence of endocarditis from 1990 to 2021 to identify influencing factors and to predict the trends of disease burden after 2021 using a Bayesian age-period-cohort (BAPC) model.

## 2. Methods

### 2.1 Data Source

We used the Global Health Data Exchange (GHDx) query tool (https://vizhub.healthdata.org/gbd-results/) to obtain estimates of mortality rates due to endocarditis based on high systolic blood pressure, including the number of deaths, disability-adjusted life years (DALYs) rates, and the number of DALYs. This includes data on 369 diseases and injuries and 87 risk factors across 204 countries and regions, with assessments based on published literature, registries, vital registration systems, verbal autopsies, and hospital records. The relevant forecast statistics for the world population from 2022 to 2040 are obtained from the GBD database (https://ghdx.healthdata.org/record/ihme-data/global-population-forecasts-2017-2100). As defined by the GBD case definitions, the included cases of endocarditis are primarily based on clinical diagnoses. According to the International Classification of Diseases (ICD) 9th or 10th edition, each death is attributed to a single underlying cause that triggers a series of death events [21, 22]. Endocarditis cases were identified using validated primary or secondary International Classification of Diseases, Ninth Revision (ICD-9) diagnostic codes (421.0, 421.1, 421.9, 112.81, 036.42, 098.84, 424.90, 424.91, and 424.99) and International Classification of Diseases, Tenth Revision (ICD-10) diagnostic codes (I330, I339, I38, I39, A3282, A3951, B376) [23, 24].

### 2.2 Descriptive Analysis

To fully understand the disease burden of endocarditis, descriptive studies were conducted at global, regional and country levels. The global number of cases, coarse and age-standardized rate (ASR) incidence, prevalence, death, and DALY for endocarditis in both sexes, men, and women from 1990 to 2021 are visually displayed. Similar comparisons were also made at the global, regional (21 GBD geographical areas) and national (204 countries and territories) levels. The SDI is a composite indicator of total fertility rate, per capita income, and years of education, reflecting the level of social development. The 204 countries and regions are categorized into five groups based on SDI quintiles: low SDI, low-middle SDI, middle SDI, high-middle SDI, and high SDI regions. The SDI index ranges from 0 to 1, where 0 indicates the lowest years of education, lowest income per capita, and highest fertility rate, while 1 indicates the opposite [25, 26].

The GBD study employs a range of estimation and modeling techniques to produce comparable results on the global burden of diseases and injuries. Comprehensive documentation of the burden estimation processes from the 2021 GBD study is available from prior studies, including key metrics such as incidence, prevalence, mortality, years of life lost (YLLs), years lived with disability (YLD), and DALYs [27, 28]. Disability weights measure the relative valuation of health states, defined as individual functional levels within a range of health domains [29]. The age-standardized data for the global population from the GBD is derived from previously published GBD studies and is used to calculate ASR [30].

### 2.3 Trend Analysis

On the basis of descriptive analysis, exploring the time trend of endocarditis disease burden is an indispensable part of epidemiological research [31]. Our goal was to explore trends in endocarditis disease burden on a global, local and multi-dimensional scale. To capture the temporal trends of ASR for incidence, mortality, and DALYs, the EAPC was calculated. ASR is represented by the natural logarithm of the regression line, formulated as follows: A linear regression model was used to calculate EAPC and its 95% confidence interval (CI), expressed as: y = α + βx + ε, EAPC = 100 × (exp (β)-1). Here, y = ln(ASR) and x = calendar year. If both EAPC and its 95% CI are >0, a rising trend is determined.

The calculation of EAPC assumes that the trend in ASR changes is linear during the observation period and is not significantly affected by sudden events, such as outbreaks, major public health policy changes, or natural disasters. This linear assumption simplifies the model; however, if there are pronounced nonlinear trends or abrupt changes in the data, the accuracy of EAPC may be compromised.

### 2.4 Frontier Analysis 

To assess the relationship between endocarditis disease burden and sociodemographic development, we applied frontier analysis as a quantitative method to determine the lowest attainable age-standardized incidence, prevalence, death, and DALY rates based on development as measured by SDI [32, 33, 34]. Statistical software SAS Enterprise Guide 7.1 (SAS Institute Inc., Cary, NC, USA) was used for analysis.

### 2.5 BAPC Prediction Analysis

BAPC analysis techniques were employed to forecast global endocarditis mortality rates from 2020 to 2030. BAPC considers the impacts of age, period, and generational cohorts on endocarditis mortality outcomes. Bayesian inference in the BAPC model utilizes second-order random effects to smooth the three aforementioned values and predict posterior rates, employing Integrated Nested Laplace Approximation (INLA) to approximate the marginal posterior distribution, thereby avoiding the mixing and convergence issues encountered by traditional Bayesian methods through Markov Chain Monte Carlo techniques. This approach has been widely used to analyze trends in chronic diseases and predict future disease burdens [35, 36, 37]. All data analyses were conducted using the open-source software R (version 4.2.1, the R Foundation for Statistical Computing, Vienna, Austria).

## 3. Results

### 3.1 Descriptive Analysis of the Burden of Endocarditis at the Global, Regional, and National Levels

**Fig. 1.**
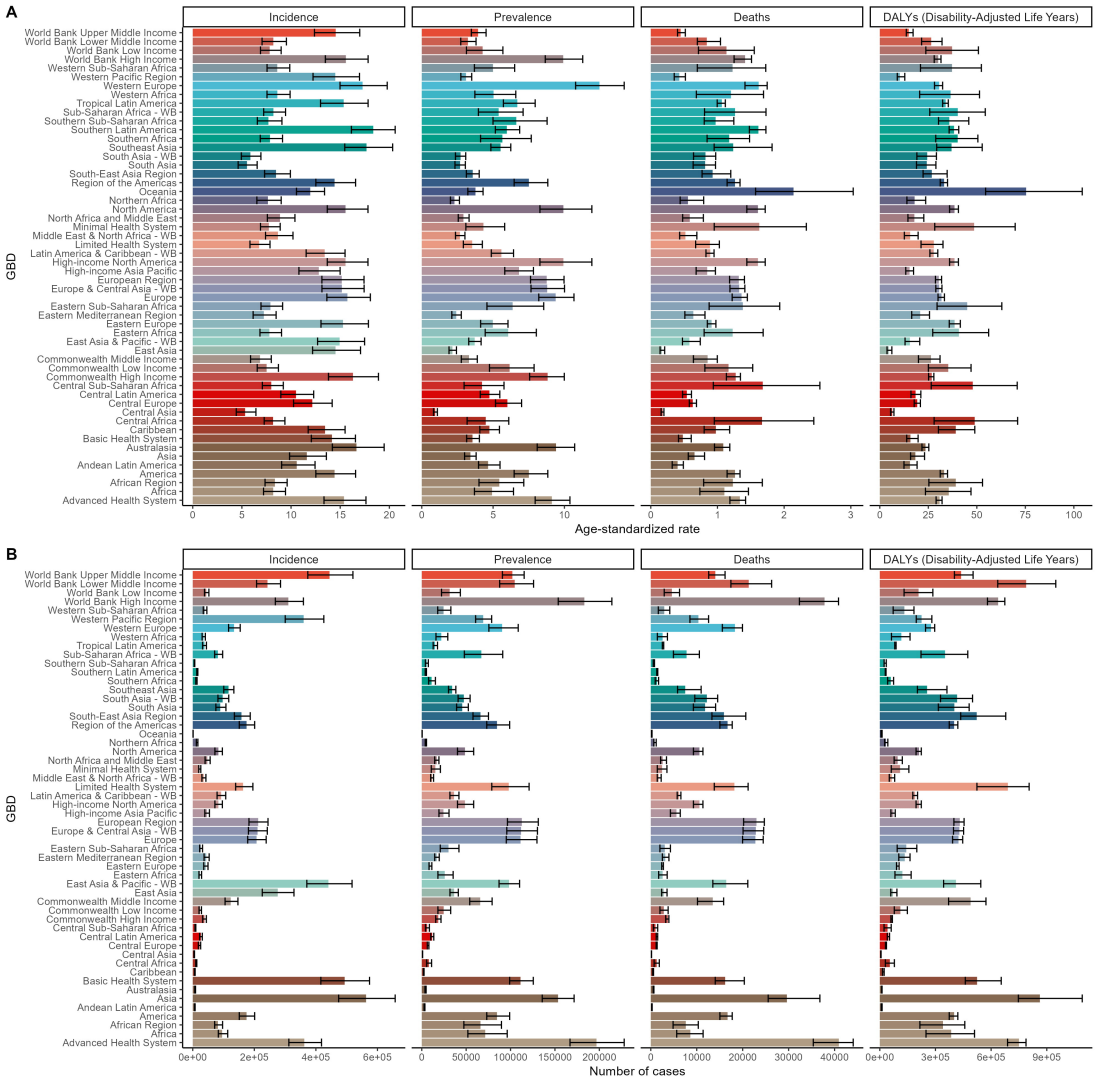
**The age-standardized rates (ASRs) (A) and total number of cases (B) of incidence, prevalence, deaths, and disability-adjusted life years (DALYs) for endocarditis in Global Burden of Disease (GBD) regions in 2021**. (A) Displays the ASRs for different GBD regions, including incidence, prevalence, deaths, and DALYs. Incidence: Incidence rates are higher in high-income regions and advanced health system areas, while lower in some low-income regions, such as sub-Saharan Africa. Prevalence: Prevalence rates are significantly higher in developed regions, such as North America, Europe, and Central Asia, reflecting the accumulation of chronic cases. Deaths: Death rates are relatively lower in developed regions. DALYs: The DALY burden is higher in resource-limited regions, such as sub-Saharan Africa, South Asia, and Central Africa, while it is lower in high-income regions. (B) Shows the total number of cases of endocarditis in different GBD regions, including incidence, prevalence, deaths, and DALYs. Incidence cases: The number of incidence cases is higher in high-income regions and populous regions, such as Asia. Prevalence cases: Prevalence cases are more numerous in advanced health systems and high-income regions, reflecting higher diagnostic levels and chronic case management. Death cases: Death cases are more prominent in high-income regions, likely due to higher diagnostic rates and the burden of disease among aging populations. DALY cases: The DALY burden is predominantly concentrated in resource-poor regions such as Central Africa and South Asia, while it is relatively smaller in high-income regions. WB, World Bank.

From a global perspective, between 1990 and 2021, the incidence, prevalence, deaths, and DALY associated with endocarditis have all shown a continuous upward trend. However, the ASR for these four indicators exhibited differing trends. Specifically, the ASR for endocarditis prevalence and incidence continued to rise, whereas the ASR for deaths and DALY displayed a downward trend (see **Supplementary Fig. 1**). This outcome may be closely linked to improvements in global healthcare standards. Fig. 1 illustrates the case numbers and ASR for the burden of endocarditis in terms of incidence, prevalence, and DALY in 2021.

Regionally, Asia reported the highest DALY cases for endocarditis at 862,935 (95% uncertainty interval (UI) 746,821 to 1,091,115); Advanced Health System had the highest number of deaths at 40,930 (95% CI 35,366 to 44,056); Asia also recorded the highest incidence cases at 563,021 (95% CI 473,893 to 657,525); and Advanced Health System had the highest prevalence cases at 196,813 (95% CI 166,977 to 227,996). The ASR for endocarditis deaths was highest in Oceania at 2.14 (95% CI 1.57 to 3.04); the ASR for DALY in Tropical Latin America was 75.41 (95% CI 54.52 to 104.32); the ASR for incidence in Southern Latin America was 18.36 (95% CI 16.14 to 20.59); and the ASR for prevalence in Western Europe was 12.46 (95% CI 10.78 to 14.19) (Fig. 1, **Supplementary Tables 1–4**). Notably, the regional distribution of the burden of endocarditis (deaths, DALY, incidence, prevalence) in terms of both case numbers and ASR was consistent.

At the national level, significant differences were observed in the incidence, prevalence, deaths, and DALY for endocarditis worldwide. Among these countries, the United States had the highest number of deaths; India had the highest number of DALY; China had the highest incidence; and the United States had the highest prevalence. China, etc., exhibited the highest ASR for DALY; China had the highest ASR for deaths; Thailand had the highest ASR for incidence (**Supplementary Fig. 2**).

### 3.2 Descriptive Analysis of the Burden of Endocarditis by Age and Sex

**Supplementary Fig. 3** presents the numbers and ASR of prevalence, incidence, deaths, and DALY for each age group in 2021. The ASR for endocarditis prevalence, incidence, deaths, and DALY increases continuously with age, peaking in the 95+ age group. The numbers for prevalence, incidence, deaths, and DALY in 2021 are more concentrated in the middle-aged and older age groups. The incidence of endocarditis is highest in the 65–69 years age group; prevalence is highest in the 70–74 years age group; DALY is highest in the 65–69 years age group; and deaths are highest in the 85–89 years age group. Between 1990 and 2021, both the prevalence and incidence rates for all age groups have shown a continuous upward trend, while the death rates and DALY rates have exhibited a downward trend in the past two years. It is noteworthy that while the numbers for prevalence, incidence, deaths, and DALY are rising across nearly all age groups, the incidence and DALYs for endocarditis in children under 5 years old continue to decline (see **Supplementary Fig. 4**). This indicates that the burden of this disease has been effectively curtailed among infants and young children.

In 2021, the numbers and ASR for endocarditis incidence, prevalence, deaths, and DALY were all higher in males than in females. The incidence, prevalence, deaths, and DALY for males were 1.27 times, 1.02 times, 1.06 times, and 1.37 times those of females, respectively. The ASR for male endocarditis incidence, prevalence, deaths, and DALY were 1.40 times, 1.14 times, 1.33 times, and 1.48 times those of females, respectively (see **Supplementary Fig. 5**). From 1990 to 2021, the indicators for males showed a higher rate of increase, particularly in incidence and DALYs. This indicates that the burden of this disease has been rising more rapidly among males (see **Supplementary Fig. 6**).

### 3.3 Overall Trends in the Burden of Endocarditis Using Comprehensive Estimation Methods

**Fig. 2.**
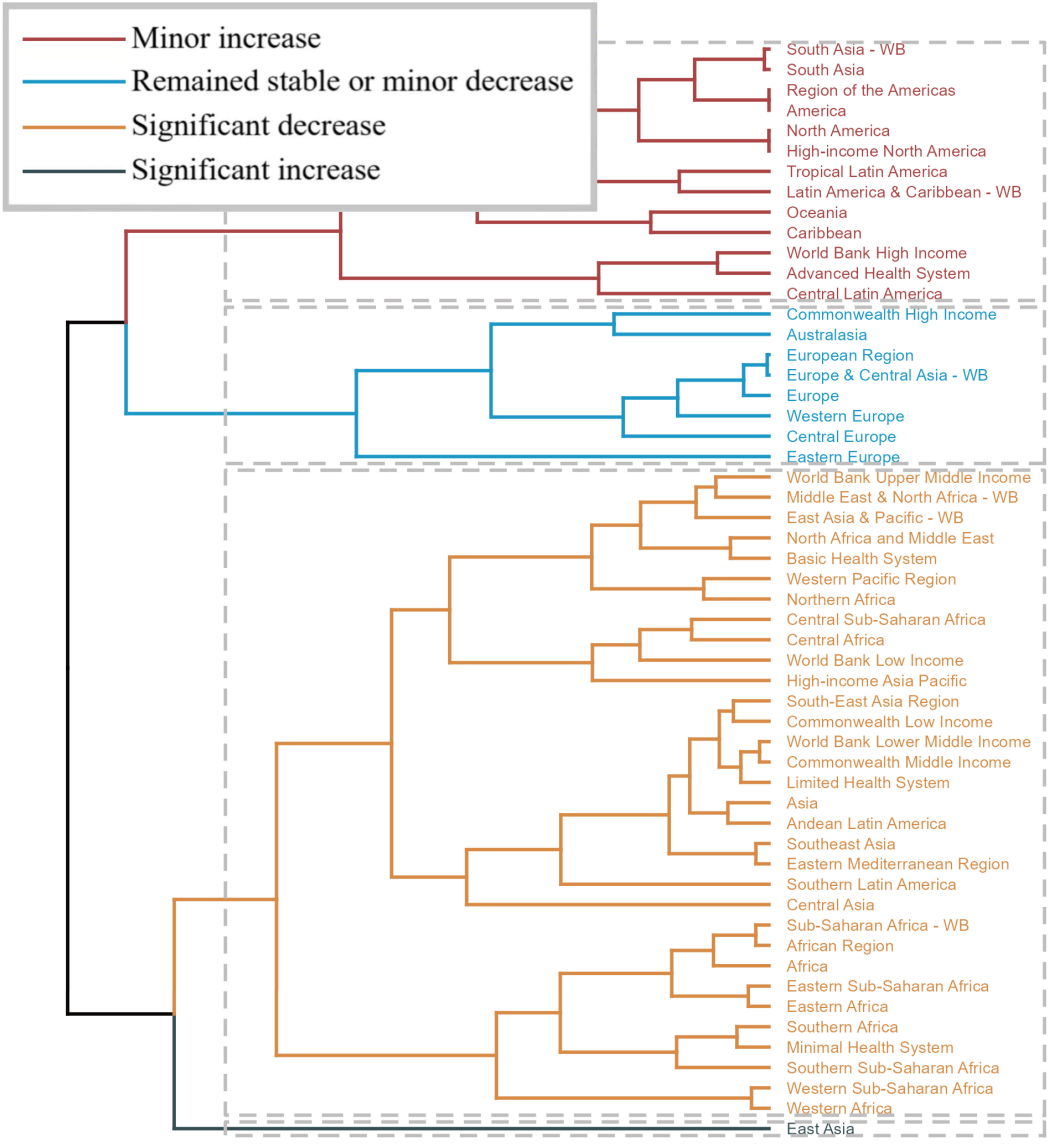
**Results of a cluster analysis based on estimated annual percentage change (EAPC) values for age-standardized mortality and disability-adjusted life years (DALYs) associated with endocarditis from 1990 to 2021**. Color Classification: Different colors represent the trend in EAPC values across regions: Dark Red (minor increase): Regions with a minor increase, such as South Asia, the Americas, North America, and Latin America & the Caribbean. Blue (remained stable or minor decrease): Regions that remained stable or experienced a minor decrease, including Commonwealth high-income countries, the European Region, and Europe & Central Asia. Orange (significant decrease): Regions with a significant decrease, such as Central Africa, Western Africa, sub-Saharan Africa, Southeast Asia, and South Asia. Black (significant increase): Regions showing a significant increase, reflecting a worsening disease burden. Cluster Structure: Regions are grouped based on trends in EAPC values, illustrating the substantial variations in the burden of endocarditis mortality and DALYs across different socioeconomic settings. Summary: This figure highlights the diversity of trends in endocarditis-related mortality and DALY burdens across global regions from 1990 to 2021. WB, World Bank.

From 1990 to 2021, globally, the ASR of incidence, prevalence, and deaths for endocarditis increased by 100% (95% CI 0.93 to 1.08), 203% (95% CI 1.9 to 2.16), and 6% (95% CI –0.1 to 0.22), respectively, while the ASR for DALY decreased by 34% (95% CI –0.45 to 0.24) (**Supplementary Tables 1–4**). The clustering analysis results based on the EAPC values for age-standardized mortality and disability-adjusted life years related to endocarditis from 1990 to 2021 are shown in Fig. 2.

The regions with a significant increase in mortality and disability-adjusted life years are East Asia, while those with a significant decrease include World Bank Upper Middle Income, Middle East & North Africa-World Bank (WB), East Asia & Pacific-WB, North Africa and Middle East, Basic Health System, Western Pacific Region, Northern Africa, Central Sub-Saharan Africa, Central Africa, World Bank Low Income, High-income Asia Pacific, South-East Asia Region, Commonwealth Low Income, World Bank Lower Middle Income, Commonwealth Middle Income, Limited Health System, Asia, Andean Latin America, Southeast Asia, Eastern Mediterranean Region, Southern Latin America, Central Asia, Sub-Saharan Africa-WB, African Region, Africa, Eastern Sub-Saharan Africa, Eastern Africa, Southern Africa, Minimal Health System, Southern Sub-Saharan Africa, Western Sub-Saharan Africa, and Western Africa.

**Fig. 3.**
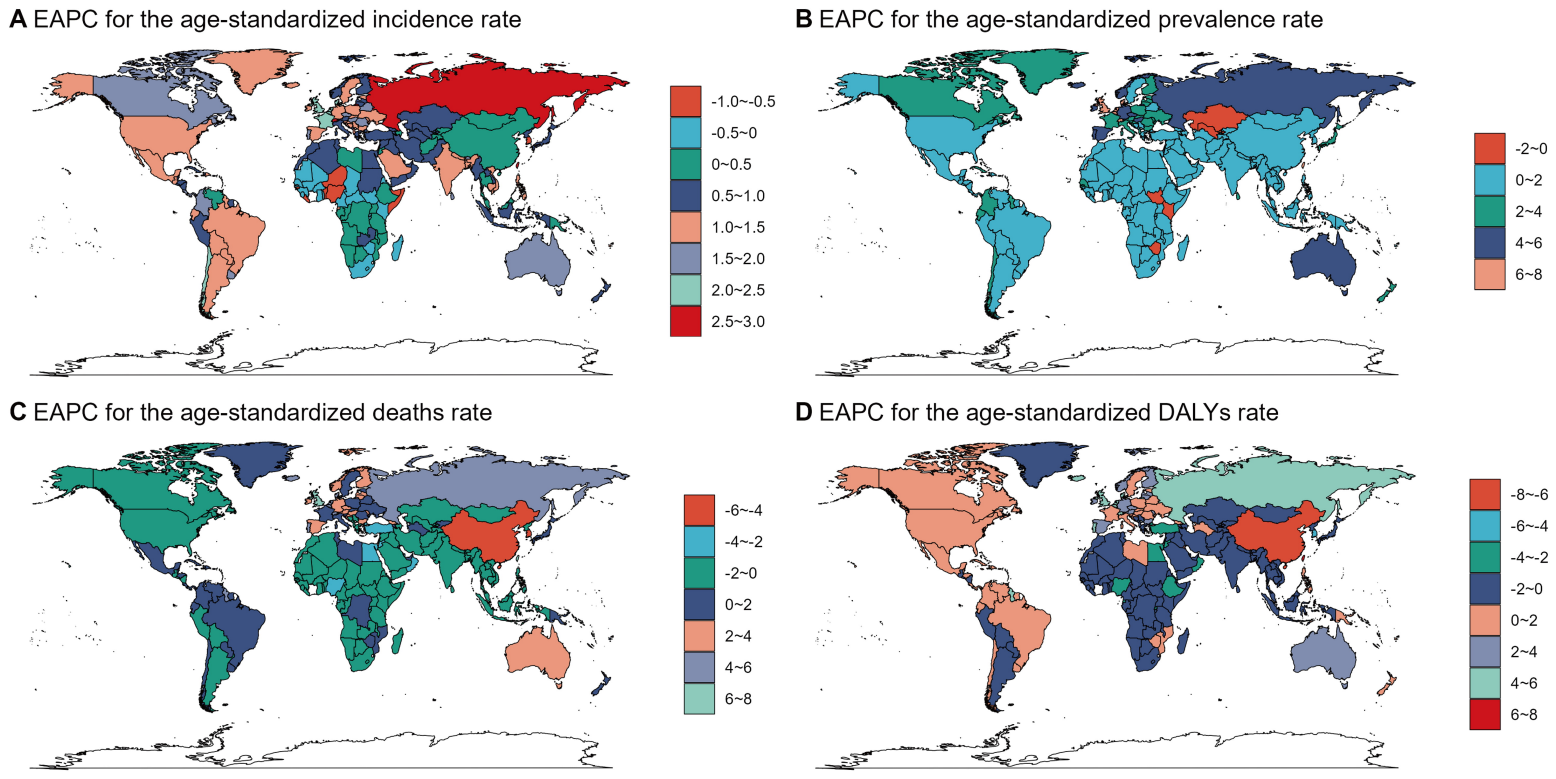
**The global distribution of age-standardized rates (ASRs) and estimated annual percentage change (EAPC) for the burden of endocarditis by country and region in 2021, including incidence (A), prevalence (B), deaths (C), and disability-adjusted life years (DALYs) (D)**. (A) (Incidence): Displays the EAPC distribution of age-standardized incidence rates across countries and regions. Certain countries, such as Russia, show significant increases (red), while Northern Europe, parts of South America, and Africa demonstrate slight or negative growth. (B) (Prevalence): The global EAPC distribution of age-standardized prevalence rates shows an increasing trend in North America, Europe, and parts of the Middle East (blue and green), whereas some African countries exhibit a declining trend (red). (C) (Deaths): The EAPC distribution of age-standardized death rates reveals a significant decline in sub-Saharan Africa and Central Asia (green and blue). (D) (DALYs): The EAPC distribution of age-standardized DALY rates indicates an upward trend in certain countries, such as Russia (light green), while some African countries demonstrate a decline (blue).

At the national level, there are significant differences in the overall trends of disease burden across 204 countries and regions (Fig. 3). The Russian Federation had the highest EAPC for incidence; Switzerland had the highest EAPC for prevalence; and the United Kingdom had the highest EAPC for deaths and DALY. From 1990 to 2021, there were differences in the changing trends of the burden of endocarditis across countries and regions, and disparities exist in disease management capabilities among these regions (see **Supplementary Fig. 7**).

### 3.4 Correlation Analysis of the Burden of Endocarditis

**Fig. 4.**
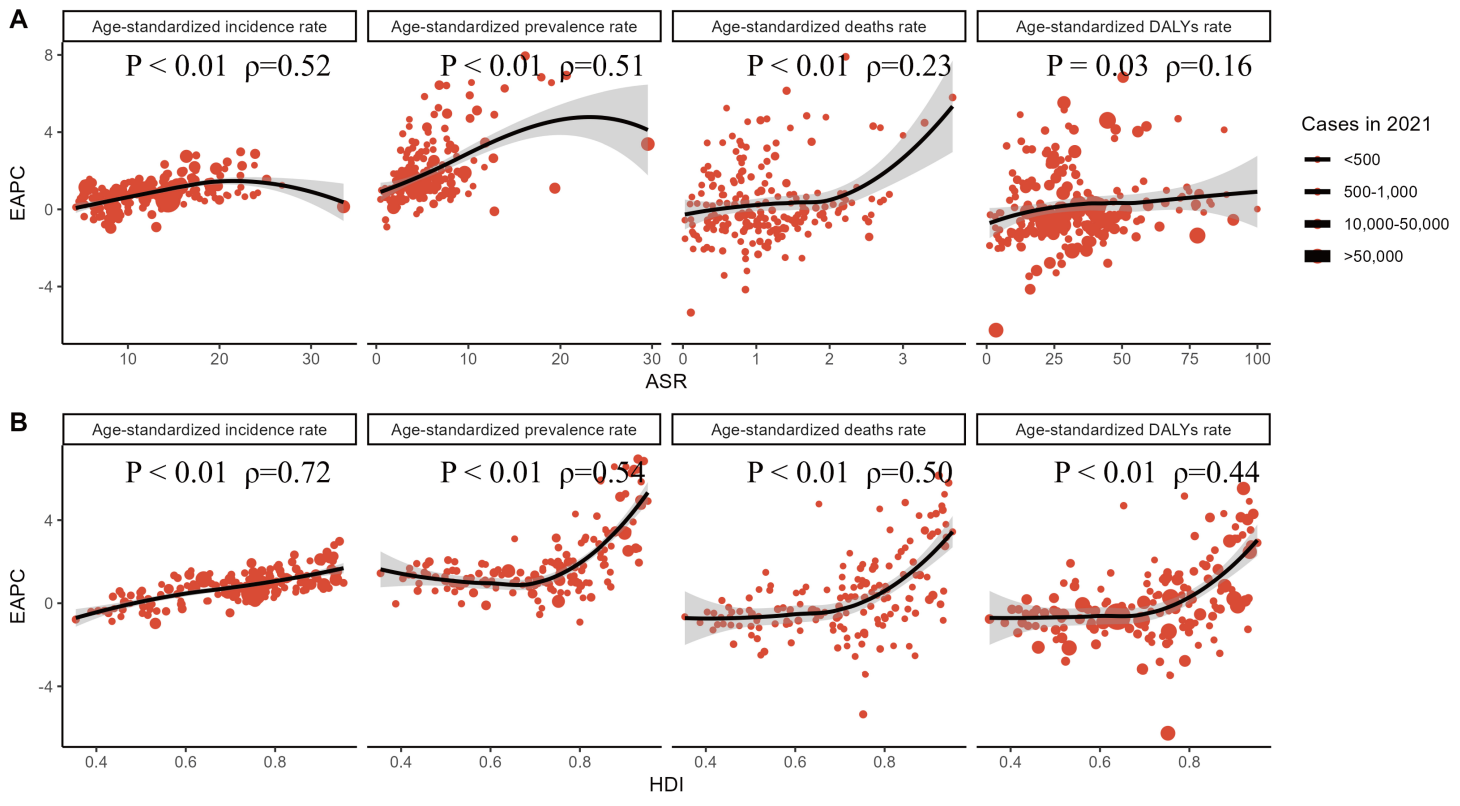
**Association between the estimated annual percentage change (EAPC) and age-standardized rates (ASRs) (A) as well as the Human Development Index (HDI) (B) for incidence, prevalence, deaths, and disability-adjusted life years (DALYs) of endocarditis in 2021**. (A) (EAPC vs. ASR): Displays the association between EAPC and ASRs. Incidence and Prevalence: EAPC shows a positive correlation with ASR (ρ = 0.52, ρ = 0.51, *p* < 0.01). Deaths: EAPC is positively correlated with ASR (ρ = 0.23, *p* < 0.01), but the correlation is weaker, indicating that changes in mortality are moderately related to baseline ASR. DALYs: EAPC has a weaker correlation with ASR (ρ = 0.16, *p* = 0.03), suggesting a relatively minor relationship between changes in disease burden and ASR. (B) (EAPC vs. HDI): Displays the association between EAPC and the HDI. Incidence: EAPC is strongly positively correlated with HDI (ρ = 0.72, *p* < 0.01). Prevalence: EAPC shows a positive correlation with HDI (ρ = 0.54, *p* < 0.01). Deaths: EAPC is positively correlated with HDI (ρ = 0.50, *p* < 0.01), with more noticeable changes in mortality rates observed in countries with higher HDI. DALYs: EAPC shows a positive correlation with HDI (ρ = 0.44, *p* < 0.01), indicating a stronger trend of annual changes in DALY burden in countries with higher HDI. Circle explanation: Each circle represents a country, and the size of the circle is proportional to the disease burden. The ρ index and *p*-values were obtained from Spearman correlation analysis.

We examined the associations between the EAPC and ASR for 2021, as well as the Human Development Index (HDI) for 2021 (Fig. 4). The results in Fig. 4 show a significant, though not completely linear, association between EAPC and ASR. When ASR is limited to relatively low levels, there is a positive correlation between the EAPC for endocarditis prevalence and incidence and ASR. In contrast, for ASR levels above a certain threshold, this association is reversed. Additionally, there is a significant positive correlation between the EAPC for endocarditis deaths and DALY and ASR. Furthermore, the EAPC is significantly positively correlated with HDI. From 1990 to 2021, countries with High SDI exhibited a faster growth rate in the EAPC for endocarditis prevalence, deaths, and DALY (see **Supplementary Fig. 8**). The burden of endocarditis, grouped by SDI, indicates that the incidence and prevalence rates are higher in high-SDI regions, while the mortality rate and DALYs are higher in low-SDI regions (see **Supplementary Fig. 9**).

There is also a significant positive correlation between the ASR for endocarditis incidence, prevalence, deaths, and DALY and regional SDI. A similar trend, though not completely linear, is observed between the ASR for endocarditis incidence, prevalence and deaths, and national SDI (see **Supplementary Figs. 10,11**).

### 3.5 Frontier Analysis of the Burden of Endocarditis

**Fig. 5.**
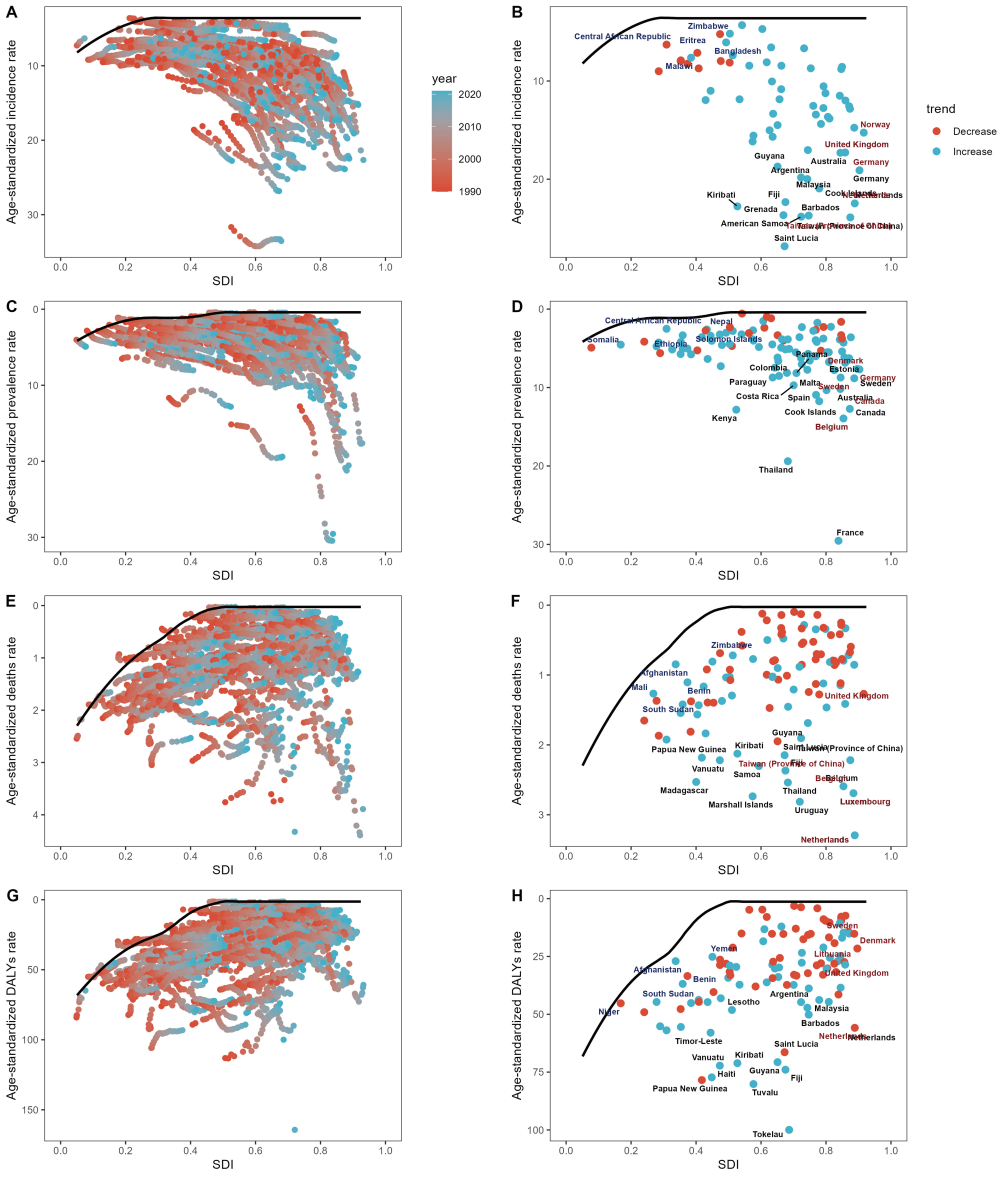
**Frontier analysis based on DALY age-standardized rates from 1990 to 2021, particularly in 2019 SDI**. (A,C,E,G) illustrate the frontier analysis based on ASR and SDI from 1990 to 2021. The color scale ranges from orange (1990) to light blue (2019). The black solid line depicts the boundary. (B,D,F,H) display the frontier analysis based on ASR and SDI for 2021. The black solid line indicates the boundary, with dots representing countries and regions. The top 15 countries and regions with the greatest effective differences are marked in black. Examples of frontier countries with low SDI (<0.5) and low effective differences are marked in blue, while examples of countries and regions with high SDI (>0.85) and relatively high effective differences are marked in red. Red dots indicate a decline in ASR, whereas blue dots indicate an increase in ASR from 1990 to 2021. Abbreviations: DALYs, disability-adjusted life years; ASR, age- standardized rate; SDI, socio-demographic index.

Fig. 5 shows the unrealized health gains in countries or regions at different levels of development from 1990 to 2021. As socio-demographic development progresses, effective differences generally increase to some extent, indicating that countries or regions with higher SDI have greater potential for burden improvement (Fig. 5). Additionally, we observed considerable heterogeneity between countries with similar SDI. Overall, the frontier remains stable when SDI >0.5.

### 3.6 Predictive Analysis of the Burden of Endocarditis by 2035

**Fig. 6.**
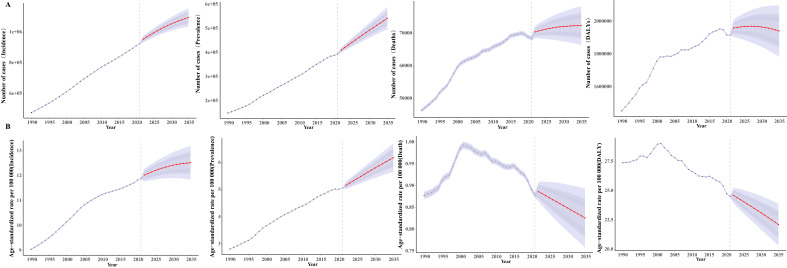
**Prediction analysis of the total number of cases (A) and age-standardized rates (ASRs) (B) for incidence, prevalence, deaths, and disability-adjusted life years (DALYs) of the burden of endocarditis**. (A) (Total number of cases): Displays actual case numbers from 1990 to 2020 and predicts trends from 2021 to 2035. Incidence cases: The total number of cases shows a significant upward trend and is expected to continue increasing in the future. Prevalence cases: The number of cases continues to rise after 2020, reflecting the cumulative effect of chronic cases. Death cases: The number of deaths peaked around 2020 and is projected to decline slightly, although it will remain at relatively high levels. DALY cases: The total number of DALY cases is expected to decrease gradually after 2020, indicating a reduction in disease burden, although regional differences may persist. (B) (ASR): Shows historical trends from 1990 to 2020 and forecasts ASR from 2021 to 2035. Incidence: ASR exhibits a continuous upward trend and is projected to keep increasing. Prevalence: ASR rises steadily post-2020, indicating a growing disease burden. Deaths: ASR shows a declining trend and is expected to continue decreasing, reflecting improvements in treatment and care. DALYs: ASR is projected to continue declining after 2020, suggesting an overall alleviation of the disease burden.

Fig. 6 shows the predicted case numbers and ASR for endocarditis incidence, prevalence, and DALY by 2035. Globally, it is expected that the case numbers for incidence, prevalence, deaths, and DALY will increase. The ASR for incidence and prevalence is also expected to show a continuous upward trend by 2035. In contrast, the ASR for endocarditis deaths and DALY is projected to gradually decline by 2035 (Fig. 6).

## 4. Discussion

This study provides the latest data on the incidence, prevalence, deaths, and DALYs of endocarditis at the global, regional, and national levels from 1990 to 2021. Based on the most recent data from the GBD database, we conducted descriptive, decomposition, and predictive analyses of the disease burden of endocarditis to comprehensively assess its development.

From 1990 to 2021, the global burden of endocarditis has shown a continuous increasing trend. Over the past thirty years, the number of deaths due to endocarditis has steadily risen, with both the number and rate of deaths being higher in males than in females. Endocarditis has a poor prognosis, and is associated with a hospital mortality of at least 25%. The 30-day mortality rate for all cases is 25.8%, and the 12-month mortality rate is 41.9% [38]. However, it is noteworthy that in a retrospective study involving 214 adult patients with infective endocarditis (131 males and 83 females), there were significant differences in the etiology of infective endocarditis between male and female patients. The microbiological differences included coagulase-negative staphylococci (15.0% in males vs. 3.8% in females, *p* = 0.011) and culture-negative endocarditis (8.7% in males vs. 23.8% in females, *p* = 0.004). The all-cause mortality rate was significantly higher in the female group compared to the male group [39]. Another nationwide study on infective endocarditis in Denmark also showed that female sex was associated with an increased in-hospital mortality rate, but there was no statistically significant difference in the 1-year and 5-year mortality rates between males and females after discharge [40].

The prevalence of endocarditis and its trends vary across different regions of the world. In 2021, among the 204 countries and regions globally, the three countries with the highest number of endocarditis-related deaths were the United States, India, and Japan. The incidence of endocarditis is expected to increase worldwide, with the highest number of cases in developed countries and the fastest growth in developing countries. The affected population will predominantly be male, but the gender gap is expected to narrow [41]. In countries with advanced healthcare systems, staphylococci have replaced streptococci as the most common cause of infective endocarditis, while this trend is less pronounced in developing countries [42]. Additionally, there are differences in the disease burden of endocarditis among countries and regions with varying income levels. In this study, the prevalence and mortality rates in high SDI regions were significantly higher than in other regions, while the incidence rate in middle SDI regions was significantly higher than in others. This finding may be closely related to the microbiological causes of endocarditis in low- and middle-income countries [43]. Economic improvements in low- and middle-income countries have led to medical advances, which may introduce new risk factors for endocarditis in these populations. In low-income countries, staphylococci have become the primary cause of endocarditis, a trend previously observed in high-income countries, potentially reflecting advances in medical technology, increased hospital exposure, and the rise of comorbidities [43, 44, 45].

According to the distribution by the four major regions of the world, the burden of endocarditis in Asia is particularly severe. Streptococcus species are the most common pathogens causing endogenous endophthalmitis due to infective endocarditis in East Asia [46]. Studies indicate that from 1990 to 2016, the prevalence of endocarditis in China increased by 26.7%, with a lower burden of cardiovascular diseases in the economically developed coastal provinces. The interprovincial disparity in the relative burden of cardiovascular diseases in China has widened, with a more rapid decrease in the economically developed provinces [47]. Asian internists and surgeons have made significant contributions in the past to determining the timing of surgery for complicated endocarditis and reducing the negative impact of intracranial hemorrhage in endocarditis patients [48].

Globally, the age-standardized mortality rate and DALYs due to endocarditis show an increasing trend with age. Recent changes in the epidemiology of endocarditis are mainly associated with an aging population and an increased frequency of risk factors [49]. However, it is noteworthy that the distribution of DALYs in children under 5 years of age is particularly prominent. Despite the gradually improving survival rates of children with congenital heart disease, the incidence is on the rise [50]. Endocarditis caused by Gram-negative bacteria is more common in children and adolescents than in adults. Additionally, compared to adults, there is a trend toward reduced mortality from complications of endocarditis in children [51].

In terms of gender differences, the burden of endocarditis is generally higher in males compared to females. In 2021, males had a higher incidence, mortality, and DALYs than females, with ASR also consistently higher. However, females exhibited more pronounced mortality rates and DALYs, suggesting that gender differences in immune response, infection pathways, and clinical outcomes may play a significant role.

Southeast Asia has among the highest incidence and mortality globally from endocarditis. In 2021, this region reported the highest number of deaths (7494.00) and the highest incidence (116,215.40), which is closely tied to limited medical resources and underdeveloped healthcare systems. In contrast, developed countries, particularly in Europe and North America, have experienced a decline in the burden of endocarditis due to advancements in diagnostic and treatment technologies. Additionally, the widespread use of medical devices, especially prosthetic heart valves, has altered the etiology of endocarditis in high-income regions, with staphylococci gradually replacing streptococci as the most common pathogens. However, this trend is less pronounced in developing countries.

Looking ahead, with the accelerating global aging population, particularly among individuals aged 85 years and older, the burden of endocarditis is expected to continue to rise, especially in high-income countries. These nations must strengthen cardiovascular disease prevention and management for elderly populations. Meanwhile, low- and middle-income countries are likely to see improvements in endocarditis treatment outcomes as their healthcare systems evolve. However, emerging risk factors, such as antimicrobial resistance, may also increase, underscoring the need for enhanced prevention and early intervention in these regions. Furthermore, gender and age differences will continue to influence the burden of endocarditis, necessitating tailored intervention strategies for different population groups. Overall, as the global health landscape evolves, the prevention and management of endocarditis will face complex challenges, requiring localized and customized approaches to effectively address diverse needs.

It is worth noting that existing studies have demonstrated the widespread application of the BAPC model in predicting epidemiological trends, with its excellent predictive performance which has been well-validated. According to our BAPC model projections, the mortality rate and DALYs attributable to endocarditis are expected to exhibit a slight downward trend over the next decade starting from 2021. However, despite the decline in age-standardized rates for these indicators, the total number of cases is projected to continue to increase. Notably, the trends for case numbers incidence, prevalence, deaths, as well as ASR for incidence and ASR for deaths, align with previous predictions based on the GBD 2019 database. These findings highlight that managing and controlling endocarditis will remain a significant challenge in the coming years.

Nonetheless, this study has certain limitations. First, since it relies on the GBD 2021 database, the analysis provides data on the global, regional, and national burden of disease but lacks individual-level data. Furthermore, it does not include critical quality-of-life metrics for endocarditis patients, underscoring the need for future research to address this important aspect. Additionally, the GBD data do not account for key risk factors such as prosthetic heart valves, previous history of endocarditis, or complex congenital heart disease, limiting the ability to analyze the independent impact of these risk factors. Data collection biases may also exist, particularly in low-income regions or areas with limited healthcare resources, potentially affecting the accuracy of global estimates.

In terms of time trend analysis, the EAPC assumes a linear extension of observed trends and does not consider potential disruptions such as advances in medical technology or changes in public health policies, which may lead to inaccuracies in prediction. Furthermore, in regions with limited or low-quality data, uncertainties in the data may affect the accuracy of the confidence intervals.

To address these limitations, future studies should aim to expand the temporal range of datasets, incorporate internal validation, and explore the development of cross-validation models to enhance the stability and generalizability of the BAPC model. Additionally, integrating nonlinear trend analyses and detecting potential breakpoint changes could provide a more comprehensive understanding of the dynamic shifts in disease burden. Future research should also prioritize factors such as quality of life, mental health, and social support to provide a more holistic assessment of the impact of endocarditis on patients.

## 5. Conclusions

In conclusion, endocarditis poses a significant disease burden globally, particularly in high SDI regions. Additionally, the evidence provided by our study indicates that men and the elderly are high-risk populations. We also found that the number of cases of morbidity and incidence is expected to continue to rise over the next 15 years, emphasizing that the burden of endocarditis remains an important public health issue that needs to be addressed.

## Availability of Data and Materials

The data used in this study came from a public database that everyone can access through the link provided in this article (https://vizhub.healthdata.org/gbd-results/).

## Supplementary Material

Supplementary material associated with this article can be found, in the online version, at https://doi.org/10.31083/RCM27168.
