# Supplementary material for: Analysis of the International, Regional, and National Endocarditis-Related Disease Burdens (1990–2021), and Changes to Projections for the Next 15 Years: A Population-Based Study
Source: Rev Cardiovasc Med. 2025 May 20;26(5):27168. doi: 10.31083/RCM27168 (PMC12135659; doi:10.31083/RCM27168)
Supplement: Supplementary file 1 [file 2153-8174-26-5-27168-s1.zip › Supplementary Tables.docx]

**Supplementary Table 1** Table 1 Data related to Incidence

|  | Number of incidence cases (95% UI) in 1990 | The age-standardized incidence rate/100000 (95% UI) in 1990 | Number of incidence cases (95% UI) in 2021 | The age-standardized incidence rate/100000 (95% UI) in 2021 | EAPC (95% CI) |
| --- | --- | --- | --- | --- | --- |
| Global | 443552 (375742-535453) | 9.35 (8.01-11.09) | 1042477 (893665-1204150) | 12.61 (10.84-14.55) | 1 (0.93-1.08) |
| Sex |  |  |  |  |  |
| Female | 210070 (176768-254383) | 8.65 (7.31-10.33) | 459198 (392157-535072) | 10.58 (9.05-12.28) | 0.65 (0.6-0.7) |
| Male | 233482 (199067-280439) | 10.11 (8.68-11.87) | 583280 (502014-670185) | 14.85 (12.84-17.06) | 1.31 (1.22-1.41) |
| Age |  |  |  |  |  |
| <5 years | 64404 (52167-79944) | 10.39 (8.41-12.9) | 49037 (40201-59719) | 7.45 (6.11-9.07) | -1.31 (-1.41--1.21) |
| 5-9 years | 36073 (25595-52428) | 6.18 (4.39-8.98) | 30525 (21364-44771) | 4.44 (3.11-6.52) | -1.44 (-1.56--1.32) |
| 10-14 years | 23111 (12674-37599) | 4.31 (2.37-7.02) | 21282 (11780-33570) | 3.19 (1.77-5.04) | -1.24 (-1.4--1.08) |
| 15-19 years | 22920 (12882-34788) | 4.41 (2.48-6.7) | 22370 (12880-33978) | 3.59 (2.06-5.45) | -0.75 (-0.85--0.65) |
| 20-24 years | 23452 (12784-35434) | 4.77 (2.6-7.2) | 25695 (14852-37851) | 4.3 (2.49-6.34) | -0.38 (-0.43--0.33) |
| 25-29 years | 24248 (13419-38251) | 5.48 (3.03-8.64) | 31579 (18559-49179) | 5.37 (3.15-8.36) | -0.12 (-0.15--0.1) |
| 30-34 years | 23213 (13331-36638) | 6.02 (3.46-9.51) | 38122 (23522-58392) | 6.31 (3.89-9.66) | 0.09 (0.06-0.13) |
| 35-39 years | 22671 (12697-35696) | 6.44 (3.6-10.13) | 39288 (22803-59621) | 7 (4.07-10.63) | 0.22 (0.17-0.26) |
| 40-44 years | 20402 (11773-30876) | 7.12 (4.11-10.78) | 41394 (25928-59135) | 8.27 (5.18-11.82) | 0.44 (0.37-0.51) |
| 45-49 years | 18711 (10917-33121) | 8.06 (4.7-14.26) | 48305 (28931-79747) | 10.2 (6.11-16.84) | 0.74 (0.63-0.85) |
| 50-54 years | 21325 (13122-32552) | 10.03 (6.17-15.31) | 62772 (41313-90080) | 14.11 (9.29-20.25) | 1.17 (1.04-1.31) |
| 55-59 years | 23462 (13872-35307) | 12.67 (7.49-19.06) | 79297 (51239-115038) | 20.04 (12.95-29.07) | 1.57 (1.43-1.72) |
| 60-64 years | 26238 (16674-37133) | 16.34 (10.38-23.12) | 91132 (63645-122850) | 28.47 (19.89-38.39) | 1.93 (1.79-2.08) |
| 65-69 years | 25656 (17028-37392) | 20.76 (13.78-30.25) | 110794 (76343-158501) | 40.17 (27.68-57.46) | 2.22 (2.08-2.36) |
| 70-74 years | 21262 (15138-29364) | 25.11 (17.88-34.68) | 108961 (80966-145595) | 52.93 (39.33-70.73) | 2.47 (2.29-2.64) |
| 75-79 years | 19004 (13207-25898) | 30.87 (21.45-42.07) | 87979 (63609-118143) | 66.71 (48.23-89.58) | 2.65 (2.43-2.87) |
| 80-84 years | 13994 (10076-18374) | 39.56 (28.48-51.94) | 71611 (52958-92325) | 81.76 (60.47-105.41) | 2.58 (2.37-2.79) |
| 85-89 years | 8385 (6062-11117) | 55.49 (40.11-73.57) | 46498 (34748-61219) | 101.7 (76-133.89) | 2.2 (2.06-2.34) |
| 90-94 years | 3706 (2835-4718) | 86.49 (66.15-110.1) | 24780 (19058-31008) | 138.52 (106.53-173.33) | 1.75 (1.63-1.87) |
| 95+ years | 1315 (884-1928) | 129.18 (86.79-189.36) | 11055 (7908-15871) | 202.83 (145.09-291.2) | 1.61 (1.5-1.73) |
| SDI region |  |  |  |  |  |
| High-middle SDI | 110269 (92249-132193) | 10.86 (9.18-12.99) | 252069 (212760-292725) | 14.7 (12.56-17.04) | 1.04 (1-1.09) |
| High SDI | 101182 (85943-119165) | 10.54 (8.98-12.5) | 274390 (236263-318814) | 15.77 (13.63-18.08) | 1.23 (1.1-1.36) |
| Low-middle SDI | 51160 (42854-62580) | 5.56 (4.72-6.61) | 120842 (103164-143215) | 7.29 (6.25-8.5) | 0.88 (0.86-0.9) |
| Low SDI | 29343 (25293-35094) | 6.61 (5.78-7.7) | 65871 (57092-78625) | 7.18 (6.34-8.3) | 0.26 (0.23-0.29) |
| Middle SDI | 151150 (127862-182316) | 10.22 (8.77-12.12) | 328360 (277094-382212) | 12.81 (10.94-14.87) | 0.75 (0.73-0.78) |
| GBD region |  |  |  |  |  |
| Advanced Health System | 141837 (119216-167476) | 9.85 (8.35-11.68) | 362375 (312176-418248) | 15.39 (13.35-17.62) | 1.45 (1.34-1.57) |
| Africa | 46064 (39726-54890) | 8.02 (7.02-9.38) | 95440 (83103-113596) | 8.19 (7.2-9.44) | -0.02 (-0.05-0.01) |
| African Region | 39770 (34268-47250) | 8.53 (7.46-9.93) | 81775 (71147-96962) | 8.36 (7.35-9.6) | -0.17 (-0.2--0.14) |
| America | 64009 (54445-76987) | 9.58 (8.18-11.4) | 174796 (151189-201100) | 14.43 (12.52-16.56) | 1.29 (1.11-1.47) |
| Andean Latin America | 2325 (2001-2762) | 7.79 (6.77-9.08) | 6579 (5603-7730) | 10.58 (9.04-12.44) | 0.98 (0.93-1.02) |
| Asia | 245395 (206206-299727) | 8.95 (7.59-10.68) | 563021 (473893-657525) | 11.6 (9.85-13.59) | 0.87 (0.85-0.89) |
| Australasia | 2419 (1986-2870) | 10.79 (8.88-12.82) | 7843 (6590-9115) | 16.65 (14.2-19.47) | 1.4 (1.27-1.53) |
| Basic Health System | 224810 (189390-271615) | 11.06 (9.45-13.16) | 493503 (416103-574246) | 14.16 (12.06-16.55) | 0.84 (0.81-0.87) |
| Caribbean | 3391 (2911-4043) | 10.64 (9.15-12.51) | 6770 (5880-7766) | 13.46 (11.74-15.5) | 0.77 (0.68-0.86) |
| Central Africa | 4513 (3880-5353) | 7.67 (6.73-8.88) | 11137 (9707-13218) | 8.17 (7.27-9.38) | 0.17 (0.13-0.22) |
| Central Asia | 2615 (2090-3248) | 4.53 (3.68-5.56) | 4718 (3889-5709) | 5.33 (4.43-6.43) | 0.6 (0.58-0.63) |
| Central Europe | 11004 (8958-13146) | 7.94 (6.56-9.5) | 21861 (18282-25734) | 12.16 (10.26-14.18) | 1.46 (1.35-1.57) |
| Central Latin America | 9274 (7717-11362) | 6.99 (5.85-8.37) | 26424 (22365-31035) | 10.5 (8.96-12.3) | 1.29 (1.14-1.43) |
| Central Sub-Saharan Africa | 3091 (2622-3716) | 6.99 (6.06-8.14) | 8342 (7198-10066) | 7.99 (7.08-9.21) | 0.44 (0.36-0.51) |
| Commonwealth High Income | 12193 (10070-14498) | 9.54 (7.96-11.53) | 37912 (32113-44226) | 16.3 (13.81-18.89) | 1.92 (1.84-2.01) |
| Commonwealth Low Income | 9504 (8043-11590) | 5.85 (5.06-6.88) | 23787 (20491-28386) | 7.51 (6.56-8.72) | 0.79 (0.77-0.81) |
| Commonwealth Middle Income | 49347 (41686-60025) | 5.35 (4.57-6.34) | 123379 (105018-146791) | 6.84 (5.88-7.99) | 0.8 (0.78-0.83) |
| East Asia | 137199 (113864-167537) | 12.25 (10.39-14.86) | 276168 (225844-328814) | 14.52 (12.19-17.07) | 0.57 (0.52-0.62) |
| East Asia & Pacific - WB | 201080 (169202-244202) | 12.04 (10.26-14.37) | 440702 (370558-517497) | 14.94 (12.7-17.48) | 0.68 (0.63-0.73) |
| Eastern Africa | 10591 (8997-12861) | 7.39 (6.4-8.68) | 23720 (20217-28766) | 7.79 (6.84-9.03) | 0.1 (0.07-0.14) |
| Eastern Europe | 20773 (16999-24757) | 8.16 (6.78-9.7) | 42342 (35726-49590) | 15.28 (13.05-17.87) | 2.37 (2.24-2.51) |
| Eastern Mediterranean Region | 17980 (15043-22089) | 5.79 (4.93-6.9) | 43845 (36750-53344) | 7.24 (6.14-8.48) | 0.77 (0.72-0.82) |
| Eastern Sub-Saharan Africa | 11699 (9972-14169) | 7.56 (6.58-8.84) | 26305 (22530-31919) | 7.9 (6.94-9.14) | 0.07 (0.03-0.11) |
| Europe | 87140 (73238-102144) | 9.57 (8.07-11.31) | 207333 (178476-238529) | 15.73 (13.64-18.07) | 1.75 (1.65-1.85) |
| Europe & Central Asia - WB | 88781 (74630-104194) | 9.34 (7.88-11.03) | 210501 (180918-242170) | 15.17 (13.14-17.43) | 1.71 (1.61-1.82) |
| European Region | 89492 (75241-105029) | 9.35 (7.88-11.04) | 212524 (182599-244481) | 15.16 (13.14-17.42) | 1.7 (1.6-1.81) |
| High-income Asia Pacific | 17677 (14789-21466) | 9.69 (8.16-11.68) | 46149 (39169-54242) | 12.82 (10.8-14.99) | 0.65 (0.47-0.82) |
| High-income North America | 31013 (26256-37182) | 10.29 (8.71-12.46) | 83307 (71986-96229) | 15.54 (13.68-17.82) | 1.22 (1.04-1.4) |
| Latin America & Caribbean - WB | 33469 (28346-40482) | 9.39 (8.02-11.01) | 92364 (79069-107140) | 13.42 (11.54-15.49) | 1.11 (0.98-1.24) |
| Limited Health System | 66442 (56021-80992) | 5.3 (4.52-6.28) | 163477 (139364-195351) | 6.75 (5.82-7.86) | 0.79 (0.76-0.82) |
| Middle East & North Africa - WB | 14629 (12222-17913) | 6.83 (5.8-8.13) | 35473 (29788-42791) | 8.66 (7.4-10.2) | 0.8 (0.74-0.87) |
| Minimal Health System | 10017 (8661-11824) | 7.77 (6.89-8.96) | 22176 (19399-26198) | 7.75 (6.89-8.89) | -0.04 (-0.06--0.01) |
| North Africa and Middle East | 19271 (16143-23521) | 6.89 (5.85-8.17) | 47329 (40042-56557) | 8.88 (7.59-10.4) | 0.87 (0.82-0.92) |
| North America | 31018 (26261-37188) | 10.29 (8.72-12.46) | 83319 (71997-96243) | 15.54 (13.69-17.83) | 1.22 (1.04-1.4) |
| Northern Africa | 6424 (5390-7833) | 6.19 (5.25-7.39) | 14001 (11795-16772) | 7.65 (6.52-9) | 0.7 (0.65-0.74) |
| Oceania | 434 (371-519) | 10.44 (9-11.98) | 1095 (958-1268) | 11.95 (10.55-13.41) | 0.52 (0.49-0.56) |
| Region of the Americas | 64009 (54445-76987) | 9.58 (8.18-11.4) | 174796 (151189-201100) | 14.43 (12.52-16.56) | 1.29 (1.11-1.47) |
| South-East Asia Region | 60894 (51424-74432) | 6.62 (5.62-7.79) | 158277 (135226-186748) | 8.47 (7.31-9.93) | 0.77 (0.75-0.78) |
| South Asia | 31498 (25612-39463) | 3.97 (3.32-4.79) | 89376 (75121-107802) | 5.52 (4.62-6.55) | 1.12 (1.07-1.17) |
| South Asia - WB | 34179 (28085-42611) | 4.21 (3.53-5.07) | 97178 (81861-116929) | 5.86 (4.94-6.93) | 1.14 (1.09-1.18) |
| Southeast Asia | 45689 (38853-54304) | 13.78 (11.89-15.96) | 116215 (100778-133542) | 17.68 (15.46-20.34) | 0.81 (0.74-0.88) |
| Southern Africa | 5937 (4986-7231) | 7.72 (6.7-9.09) | 11675 (9998-14072) | 7.86 (6.87-9.14) | -0.04 (-0.08-0) |
| Southern Latin America | 5452 (4706-6318) | 11.71 (10.14-13.49) | 14804 (13022-16591) | 18.36 (16.14-20.59) | 1.36 (1.21-1.51) |
| Southern Sub-Saharan Africa | 3588 (2986-4370) | 8.23 (7.05-9.75) | 5481 (4640-6621) | 7.7 (6.57-9.07) | -0.35 (-0.42--0.29) |
| Sub-Saharan Africa - WB | 39814 (34309-47340) | 8.47 (7.41-9.86) | 81726 (71104-97171) | 8.21 (7.22-9.44) | -0.21 (-0.24--0.18) |
| Tropical Latin America | 13151 (11049-16005) | 10.35 (8.79-12.18) | 38037 (31985-44488) | 15.36 (13-17.84) | 1.26 (1.12-1.4) |
| Western Africa | 18599 (16105-21822) | 10.17 (8.9-11.74) | 34907 (30443-40934) | 8.61 (7.55-9.9) | -0.71 (-0.77--0.66) |
| Western Europe | 51579 (43533-60081) | 10.92 (9.25-12.88) | 134147 (116364-154181) | 17.28 (14.98-19.78) | 1.56 (1.46-1.66) |
| Western Pacific Region | 168628 (141115-205866) | 11.97 (10.17-14.42) | 360283 (300433-425769) | 14.5 (12.24-16.96) | 0.59 (0.53-0.65) |
| Western Sub-Saharan Africa | 20412 (17681-23927) | 10.08 (8.84-11.63) | 39183 (34219-45872) | 8.6 (7.56-9.87) | -0.68 (-0.73--0.63) |
| World Bank High Income | 117193 (99057-138168) | 10.28 (8.73-12.24) | 310508 (268295-359470) | 15.58 (13.49-17.84) | 1.3 (1.17-1.42) |
| World Bank Low Income | 20124 (17293-24042) | 7.3 (6.4-8.53) | 43963 (38135-52460) | 7.83 (6.89-8.99) | 0.21 (0.19-0.24) |
| World Bank Lower Middle Income | 100104 (84234-121629) | 6.42 (5.45-7.58) | 242774 (207356-285150) | 8.2 (7.05-9.53) | 0.81 (0.78-0.83) |
| World Bank Upper Middle Income | 205681 (172819-248800) | 11.33 (9.64-13.54) | 444280 (373682-520289) | 14.54 (12.38-16.99) | 0.84 (0.81-0.87) |
| Country |  |  |  |  |  |
| Afghanistan | 479 (402-589) | 4.95 (4.23-6.01) | 1366 (1135-1703) | 5.3 (4.54-6.29) | 0.26 (0.21-0.31) |
| Albania | 205 (168-248) | 8.07 (6.59-9.69) | 405 (329-484) | 10.75 (8.9-12.66) | 1.17 (1.05-1.29) |
| Algeria | 1355 (1120-1656) | 6.69 (5.61-8) | 3378 (2844-4003) | 8.41 (7.09-9.9) | 0.76 (0.73-0.79) |
| American Samoa | 4 (4-5) | 13.53 (11.77-15.64) | 11 (10-12) | 23.82 (21.25-26.78) | 2.29 (2.17-2.4) |
| Andorra | 6 (5-7) | 9.83 (8.14-11.74) | 20 (16-24) | 14.78 (12.45-17.51) | 1.34 (1.21-1.46) |
| Angola | 546 (463-669) | 6.65 (5.78-7.71) | 1883 (1605-2296) | 7.65 (6.73-8.77) | 0.44 (0.37-0.5) |
| Antigua and Barbuda | 8 (7-10) | 14.28 (12.42-16.64) | 18 (16-21) | 18.7 (16.45-21.39) | 0.84 (0.76-0.93) |
| Argentina | 4197 (3628-4836) | 13.15 (11.43-15.09) | 10360 (9153-11685) | 19.82 (17.42-22.34) | 1.21 (1.02-1.4) |
| Armenia | 135 (107-167) | 4.42 (3.53-5.45) | 197 (159-236) | 5.23 (4.3-6.33) | 0.64 (0.6-0.68) |
| Australia | 2030 (1675-2424) | 10.81 (8.92-12.8) | 6875 (5751-8012) | 17.31 (14.69-20.3) | 1.51 (1.35-1.67) |
| Austria | 1165 (1008-1330) | 11.88 (10.35-13.56) | 2220 (1927-2539) | 15.47 (13.47-17.83) | 0.7 (0.63-0.76) |
| Azerbaijan | 260 (205-329) | 4.21 (3.34-5.22) | 491 (391-610) | 4.65 (3.81-5.65) | 0.41 (0.37-0.45) |
| Bahamas | 34 (30-40) | 16.39 (14.36-18.8) | 78 (68-90) | 19.52 (17.16-22.47) | 0.59 (0.51-0.67) |
| Bahrain | 27 (21-33) | 7.53 (6.42-8.99) | 111 (89-139) | 8.71 (7.29-10.48) | 0.53 (0.47-0.59) |
| Bangladesh | 2862 (2362-3589) | 4.08 (3.44-4.89) | 8874 (7450-10618) | 6.07 (5.15-7.17) | 1.37 (1.34-1.4) |
| Barbados | 49 (43-57) | 18.6 (16.27-21.31) | 96 (84-110) | 23.71 (20.99-27.04) | 0.7 (0.62-0.78) |
| Belarus | 1063 (865-1276) | 8.87 (7.35-10.54) | 1928 (1619-2285) | 14.37 (12.17-16.94) | 1.84 (1.74-1.94) |
| Belgium | 1331 (1104-1604) | 9.9 (8.18-12.01) | 4420 (3750-5112) | 20.85 (17.7-24.15) | 2.46 (2.18-2.74) |
| Belize | 13 (11-16) | 8.28 (6.87-10.04) | 39 (32-47) | 10.27 (8.74-12.11) | 0.67 (0.56-0.78) |
| Benin | 541 (465-632) | 9.5 (8.43-10.82) | 1111 (962-1290) | 8.24 (7.28-9.47) | -0.57 (-0.62--0.52) |
| Bermuda | 9 (8-11) | 14.95 (12.82-17.48) | 19 (17-23) | 19.3 (16.66-22.31) | 0.84 (0.77-0.91) |
| Bhutan | 15 (12-19) | 3.69 (3.06-4.45) | 39 (33-47) | 5.8 (4.88-6.87) | 1.57 (1.53-1.61) |
| Bolivia (Plurinational State of) | 349 (296-416) | 6.72 (5.74-7.94) | 944 (812-1108) | 9.21 (7.95-10.71) | 1.08 (1.01-1.15) |
| Bosnia and Herzegovina | 325 (257-399) | 7.39 (6-8.93) | 477 (390-576) | 9.5 (7.81-11.37) | 1.03 (0.88-1.18) |
| Botswana | 77 (63-97) | 7.28 (6.21-8.68) | 162 (136-193) | 7.75 (6.66-8.99) | 0.12 (0.07-0.18) |
| Brazil | 12826 (10770-15607) | 10.35 (8.79-12.18) | 37023 (31124-43329) | 15.35 (12.99-17.83) | 1.25 (1.11-1.4) |
| Brunei Darussalam | 18 (15-21) | 10.64 (9.33-12.16) | 55 (46-64) | 14.2 (12.27-16.32) | 0.95 (0.87-1.03) |
| Bulgaria | 859 (704-1028) | 8.39 (7.02-9.97) | 1217 (1024-1428) | 12.28 (10.63-14.18) | 1.4 (1.32-1.48) |
| Burkina Faso | 967 (825-1129) | 9.51 (8.44-10.86) | 1925 (1699-2208) | 9 (8-10.15) | -0.14 (-0.24--0.04) |
| Burundi | 356 (308-426) | 7.77 (6.91-9) | 772 (661-934) | 7.11 (6.3-8.21) | -0.36 (-0.42--0.31) |
| Cabo Verde | 40 (34-47) | 9.62 (8.28-11.19) | 50 (43-59) | 10.28 (8.89-11.92) | 0.33 (0.27-0.38) |
| Cambodia | 577 (475-708) | 8.23 (6.93-9.75) | 1629 (1401-1903) | 11.41 (9.87-13.09) | 1.21 (1.16-1.27) |
| Cameroon | 996 (857-1179) | 9.36 (8.2-10.75) | 2440 (2120-2876) | 8.65 (7.59-9.9) | -0.39 (-0.43--0.35) |
| Canada | 2640 (2177-3170) | 8.76 (7.28-10.58) | 8631 (7206-10202) | 14.01 (11.74-16.4) | 1.63 (1.44-1.82) |
| Central African Republic | 141 (119-171) | 6.58 (5.75-7.68) | 264 (226-322) | 6.29 (5.57-7.28) | -0.17 (-0.19--0.16) |
| Chad | 603 (521-712) | 8.88 (7.85-10.19) | 1446 (1255-1687) | 8.06 (7.14-9.13) | -0.37 (-0.41--0.33) |
| Chile | 773 (645-934) | 6.67 (5.64-7.97) | 3337 (2917-3812) | 14.01 (12.28-15.88) | 2.33 (2.25-2.41) |
| China | 133846 (111080-163558) | 12.34 (10.47-14.97) | 264282 (216083-315405) | 14.38 (12.03-16.92) | 0.49 (0.44-0.55) |
| Colombia | 2269 (1911-2744) | 8.7 (7.38-10.26) | 8404 (7185-9722) | 15.88 (13.65-18.41) | 1.93 (1.77-2.1) |
| Comoros | 31 (27-38) | 8.23 (7.21-9.49) | 52 (45-61) | 7.96 (6.92-9.26) | -0.12 (-0.19--0.05) |
| Congo | 146 (123-176) | 7.46 (6.53-8.7) | 364 (312-434) | 8.52 (7.49-9.81) | 0.42 (0.35-0.5) |
| Cook Islands | 3 (2-3) | 17.19 (15.12-19.56) | 5 (4-5) | 20.94 (18.45-23.78) | 0.7 (0.65-0.75) |
| Costa Rica | 281 (238-333) | 12.3 (10.52-14.3) | 1063 (917-1230) | 20.08 (17.36-23.23) | 1.74 (1.67-1.81) |
| Croatia | 454 (364-557) | 8.14 (6.66-9.86) | 803 (681-948) | 11.24 (9.59-13.18) | 1.21 (1.16-1.26) |
| Cuba | 1153 (972-1384) | 11.2 (9.48-13.36) | 1961 (1645-2290) | 13.57 (11.68-16.03) | 0.58 (0.52-0.64) |
| Cyprus | 100 (85-116) | 14.36 (12.26-16.28) | 321 (270-376) | 17.31 (14.86-20.05) | 0.64 (0.52-0.76) |
| Czechia | 1024 (845-1225) | 8.54 (7.09-10.28) | 2467 (2041-2898) | 14.25 (11.82-16.59) | 1.83 (1.7-1.95) |
| Côte d'Ivoire | 1319 (1124-1533) | 10.07 (8.84-11.53) | 2335 (2040-2702) | 8.97 (7.95-10.24) | -0.51 (-0.55--0.46) |
| Democratic People's Republic of Korea | 1521 (1278-1811) | 8.11 (6.79-9.57) | 2802 (2343-3343) | 10.06 (8.6-11.81) | 0.74 (0.68-0.79) |
| Democratic Republic of the Congo | 2165 (1830-2606) | 7.04 (6.09-8.23) | 5587 (4809-6706) | 8.11 (7.14-9.38) | 0.48 (0.39-0.56) |
| Denmark | 655 (548-778) | 9.73 (8.07-11.67) | 1672 (1409-1977) | 17.38 (14.61-20.46) | 2.04 (1.89-2.19) |
| Djibouti | 26 (22-32) | 8.17 (7.09-9.52) | 85 (71-101) | 8.34 (7.22-9.67) | -0.01 (-0.09-0.06) |
| Dominica | 10 (9-12) | 15.74 (13.79-17.89) | 14 (12-16) | 18.38 (16.32-21.12) | 0.43 (0.34-0.52) |
| Dominican Republic | 536 (454-641) | 8.91 (7.6-10.58) | 1299 (1121-1521) | 12.26 (10.64-14.28) | 1.04 (0.93-1.15) |
| Ecuador | 568 (484-672) | 7.25 (6.29-8.45) | 1855 (1591-2161) | 10.89 (9.38-12.67) | 1.22 (1.16-1.28) |
| Egypt | 2763 (2338-3357) | 5.7 (4.84-6.82) | 5977 (4957-7282) | 6.95 (5.88-8.19) | 0.65 (0.58-0.72) |
| El Salvador | 279 (230-338) | 6.33 (5.24-7.53) | 536 (448-641) | 8.44 (7.05-10.1) | 0.87 (0.71-1.03) |
| Equatorial Guinea | 24 (20-29) | 6.84 (5.94-7.95) | 103 (86-124) | 9.59 (8.33-10.98) | 1.28 (1.21-1.35) |
| Eritrea | 205 (173-251) | 7.51 (6.62-8.72) | 374 (322-453) | 7.13 (6.32-8.32) | -0.26 (-0.31--0.21) |
| Estonia | 218 (182-255) | 11.61 (9.67-13.5) | 355 (296-418) | 17.05 (14.27-19.99) | 1.33 (1.22-1.45) |
| Eswatini | 41 (33-52) | 6.54 (5.53-7.83) | 63 (52-77) | 6.53 (5.66-7.67) | -0.04 (-0.07--0.01) |
| Ethiopia | 2685 (2248-3285) | 6.63 (5.7-7.79) | 5806 (4877-7089) | 6.88 (5.91-8.1) | 0.06 (0.01-0.1) |
| Fiji | 84 (72-98) | 16.77 (14.81-19.08) | 171 (153-193) | 22.34 (20.13-24.87) | 1.15 (1.07-1.23) |
| Finland | 541 (449-653) | 8.92 (7.45-10.88) | 1123 (936-1346) | 11.99 (10.07-14.35) | 0.73 (0.53-0.94) |
| France | 8027 (6711-9380) | 10.88 (9.08-12.8) | 24514 (20867-28241) | 19.63 (16.71-22.8) | 2.11 (1.9-2.32) |
| Gabon | 70 (60-83) | 8.37 (7.39-9.73) | 142 (122-170) | 9.72 (8.51-11.19) | 0.37 (0.33-0.42) |
| Gambia | 90 (77-107) | 8.89 (7.79-10.34) | 175 (150-208) | 8.39 (7.32-9.66) | -0.28 (-0.3--0.25) |
| Georgia | 339 (270-417) | 5.77 (4.63-7.11) | 320 (267-378) | 6.67 (5.61-7.81) | 0.54 (0.42-0.65) |
| Germany | 13176 (11212-15065) | 12.94 (11.19-14.96) | 27423 (23924-31890) | 19.1 (16.58-21.81) | 1.13 (1.05-1.22) |
| Ghana | 1924 (1688-2199) | 12.67 (11.33-14.25) | 4062 (3642-4632) | 12.61 (11.35-14.23) | -0.27 (-0.36--0.18) |
| Greece | 1263 (1032-1539) | 9.93 (8.16-12.05) | 2437 (2098-2829) | 12.75 (10.99-14.89) | 0.86 (0.78-0.95) |
| Greenland | 3 (2-4) | 6.73 (5.68-8.02) | 6 (5-7) | 10.06 (8.77-11.45) | 1.41 (1.17-1.65) |
| Grenada | 14 (13-16) | 18.11 (15.96-20.45) | 25 (22-29) | 23.66 (21.23-26.72) | 0.77 (0.62-0.92) |
| Guam | 12 (10-14) | 12.33 (10.54-14.38) | 37 (33-42) | 20.59 (18.22-23.35) | 2.1 (1.97-2.23) |
| Guatemala | 356 (293-440) | 5.17 (4.22-6.32) | 975 (803-1185) | 7.17 (5.95-8.49) | 1.08 (0.93-1.22) |
| Guinea | 685 (597-796) | 10.01 (8.97-11.39) | 1216 (1067-1388) | 9.19 (8.21-10.36) | -0.31 (-0.35--0.27) |
| Guinea-Bissau | 100 (85-118) | 9.36 (8.23-10.65) | 150 (129-176) | 7.9 (7.01-9.01) | -0.64 (-0.67--0.62) |
| Guyana | 74 (65-88) | 11.54 (10.13-13.23) | 132 (118-149) | 18.72 (16.91-21.08) | 1.43 (1.32-1.54) |
| Haiti | 483 (420-570) | 8.58 (7.6-10.02) | 1058 (917-1256) | 9.88 (8.65-11.42) | 0.52 (0.45-0.58) |
| Honduras | 208 (165-264) | 5.59 (4.46-6.92) | 625 (505-779) | 7.38 (6.15-8.92) | 0.93 (0.77-1.08) |
| Hungary | 1182 (972-1412) | 9.04 (7.52-10.75) | 2718 (2251-3220) | 16.07 (13.49-18.62) | 1.99 (1.79-2.18) |
| Iceland | 23 (18-27) | 8.44 (6.88-10.23) | 58 (48-68) | 12.28 (10.17-14.7) | 1.22 (0.98-1.45) |
| India | 24723 (19847-31069) | 3.92 (3.25-4.75) | 70294 (58906-84882) | 5.48 (4.57-6.53) | 1.15 (1.09-1.2) |
| Indonesia | 13750 (11339-17043) | 9.93 (8.32-11.84) | 30333 (25295-35514) | 11.86 (10.07-13.83) | 0.53 (0.46-0.6) |
| Iran (Islamic Republic of) | 3782 (3133-4665) | 8.04 (6.8-9.56) | 7973 (6660-9520) | 9.92 (8.43-11.69) | 0.72 (0.63-0.81) |
| Iraq | 1007 (839-1233) | 6.41 (5.38-7.66) | 2837 (2401-3448) | 8.44 (7.18-10) | 0.97 (0.93-1) |
| Ireland | 328 (270-394) | 8.71 (7.13-10.51) | 863 (719-1018) | 12.83 (10.77-15.09) | 1.35 (1.24-1.47) |
| Israel | 539 (459-637) | 11.12 (9.43-13.11) | 1621 (1398-1873) | 14.63 (12.54-16.96) | 0.81 (0.66-0.96) |
| Italy | 7131 (5800-8761) | 11.38 (9.16-14.17) | 16006 (13727-18348) | 13.65 (11.96-15.56) | 0.91 (0.66-1.17) |
| Jamaica | 252 (216-301) | 12.22 (10.34-14.47) | 571 (497-657) | 19.08 (16.69-22.16) | 1.45 (1.36-1.55) |
| Japan | 12801 (10468-15706) | 9.19 (7.56-11.26) | 34482 (29055-40708) | 12.57 (10.58-14.77) | 0.88 (0.71-1.06) |
| Jordan | 274 (231-331) | 10.97 (9.32-12.66) | 1131 (963-1362) | 11.63 (10-13.61) | 0.08 (0.02-0.15) |
| Kazakhstan | 706 (573-876) | 4.88 (3.98-6.01) | 1172 (976-1410) | 6.37 (5.36-7.56) | 0.96 (0.93-0.99) |
| Kenya | 1589 (1305-1987) | 7.93 (6.75-9.49) | 3358 (2812-4105) | 8.34 (7.13-9.78) | -0.05 (-0.11-0.02) |
| Kiribati | 10 (9-11) | 18.6 (16.85-20.97) | 20 (18-22) | 22.77 (20.5-25.15) | 0.66 (0.64-0.68) |
| Kuwait | 99 (81-124) | 7.95 (6.68-9.55) | 391 (316-481) | 10.5 (8.83-12.49) | 1.06 (0.98-1.14) |
| Kyrgyzstan | 151 (120-189) | 4.13 (3.29-5.13) | 269 (218-331) | 4.7 (3.89-5.7) | 0.42 (0.41-0.44) |
| Lao People's Democratic Republic | 245 (203-301) | 8.17 (6.88-9.73) | 597 (509-711) | 10.24 (8.82-11.87) | 0.79 (0.74-0.83) |
| Latvia | 316 (262-376) | 9.93 (8.18-11.76) | 375 (310-446) | 12.93 (10.72-15.35) | 0.73 (0.67-0.79) |
| Lebanon | 207 (173-248) | 7.72 (6.54-9.12) | 551 (469-653) | 9.6 (8.17-11.39) | 0.77 (0.74-0.81) |
| Lesotho | 79 (65-99) | 6.06 (5.12-7.37) | 97 (82-119) | 6.19 (5.35-7.25) | 0.1 (0.08-0.13) |
| Liberia | 280 (239-328) | 9.94 (8.81-11.37) | 365 (316-438) | 7.94 (6.92-9.18) | -0.87 (-0.92--0.83) |
| Libya | 297 (250-354) | 7.29 (6.19-8.56) | 457 (381-557) | 7.72 (6.63-9.07) | 0.29 (0.24-0.33) |
| Lithuania | 371 (304-443) | 8.9 (7.29-10.61) | 526 (443-614) | 12.31 (10.32-14.53) | 0.99 (0.93-1.05) |
| Luxembourg | 50 (43-58) | 10.67 (9.15-12.41) | 190 (164-217) | 19.73 (17.1-22.63) | 1.98 (1.83-2.12) |
| Madagascar | 969 (848-1133) | 10.15 (9.07-11.61) | 2248 (1954-2632) | 10.03 (8.96-11.41) | -0.12 (-0.18--0.07) |
| Malawi | 560 (472-677) | 6.76 (5.86-7.93) | 1165 (983-1416) | 7.63 (6.66-8.82) | 0.37 (0.35-0.4) |
| Malaysia | 2318 (2001-2715) | 17.67 (15.54-20.12) | 6137 (5375-7152) | 19.99 (17.6-22.99) | 0.49 (0.45-0.52) |
| Maldives | 17 (14-20) | 11.41 (9.66-13.53) | 63 (52-76) | 14.49 (12.36-16.83) | 0.8 (0.69-0.91) |
| Mali | 889 (760-1034) | 8.99 (7.98-10.29) | 1909 (1660-2229) | 7.83 (6.86-8.94) | -0.47 (-0.53--0.42) |
| Malta | 42 (35-49) | 10.6 (9.04-12.56) | 145 (125-167) | 19.75 (17.2-22.79) | 2.04 (1.88-2.19) |
| Marshall Islands | 3 (3-4) | 11.51 (10.09-13.05) | 6 (6-7) | 16.16 (14.31-18.23) | 1.18 (1.14-1.22) |
| Mauritania | 200 (170-235) | 9.45 (8.23-10.94) | 372 (317-435) | 9.07 (7.79-10.43) | -0.29 (-0.38--0.21) |
| Mauritius | 95 (77-117) | 10.32 (8.5-12.39) | 231 (197-270) | 14.69 (12.6-17.18) | 1.27 (1.13-1.41) |
| Mexico | 4431 (3608-5520) | 6.21 (5.07-7.58) | 11145 (9272-13350) | 8.72 (7.33-10.41) | 1.06 (0.9-1.22) |
| Micronesia (Federated States of) | 8 (7-9) | 11.15 (9.78-12.67) | 13 (11-15) | 16.94 (15.02-19.08) | 1.45 (1.41-1.48) |
| Monaco | 11 (9-12) | 18.59 (16.05-21.37) | 20 (17-23) | 24.18 (21.13-27.55) | 0.86 (0.8-0.91) |
| Mongolia | 66 (53-83) | 4.26 (3.5-5.26) | 134 (108-164) | 4.72 (3.84-5.64) | 0.38 (0.33-0.43) |
| Montenegro | 53 (42-64) | 8.4 (6.82-10.28) | 88 (72-106) | 10.37 (8.54-12.47) | 0.94 (0.84-1.03) |
| Morocco | 1321 (1087-1634) | 5.95 (4.96-7.2) | 2695 (2255-3203) | 7.62 (6.45-8.94) | 0.84 (0.81-0.87) |
| Mozambique | 810 (686-983) | 7.32 (6.39-8.51) | 1935 (1642-2351) | 8.11 (7.18-9.43) | 0.32 (0.3-0.34) |
| Myanmar | 2718 (2261-3277) | 9.09 (7.68-10.7) | 6079 (5214-7048) | 11.86 (10.26-13.65) | 0.97 (0.91-1.04) |
| Namibia | 84 (70-104) | 7.53 (6.45-8.9) | 162 (137-197) | 8.09 (6.98-9.63) | 0.1 (0.04-0.17) |
| Nauru | 1 (1-1) | 12.44 (11.07-13.99) | 1 (1-1) | 15.64 (13.98-17.55) | 0.75 (0.7-0.8) |
| Nepal | 487 (393-609) | 3.57 (2.99-4.35) | 1348 (1121-1617) | 5.21 (4.41-6.15) | 1.34 (1.3-1.37) |
| Netherlands | 2061 (1742-2400) | 11.21 (9.42-13.16) | 7172 (6186-8258) | 22.47 (19.35-25.84) | 2.26 (1.88-2.63) |
| New Zealand | 389 (319-472) | 10.64 (8.71-13) | 968 (821-1132) | 13.2 (11.3-15.45) | 0.66 (0.61-0.71) |
| Nicaragua | 213 (177-260) | 7.98 (6.68-9.51) | 603 (508-715) | 10.54 (8.9-12.38) | 0.94 (0.91-0.97) |
| Niger | 804 (695-947) | 9.18 (8.14-10.48) | 1748 (1512-2059) | 7.49 (6.62-8.6) | -0.78 (-0.86--0.71) |
| Nigeria | 9419 (8014-11184) | 10.28 (8.78-12.04) | 17177 (14612-20668) | 8.12 (6.97-9.54) | -0.97 (-1.04--0.91) |
| Niue | 0 (0-0) | 14.03 (12.27-15.99) | 0 (0-0) | 17.58 (15.54-20.02) | 0.75 (0.73-0.78) |
| North Macedonia | 133 (106-163) | 6.86 (5.52-8.37) | 263 (211-320) | 9.07 (7.45-10.85) | 1.06 (0.95-1.18) |
| Northern Mariana Islands | 4 (3-4) | 13.42 (11.43-15.52) | 6 (5-7) | 13.65 (11.53-15.99) | 0 (-0.06-0.07) |
| Norway | 595 (495-711) | 11.6 (9.65-14.06) | 1322 (1105-1572) | 15.26 (12.84-17.91) | 0.98 (0.9-1.06) |
| Oman | 123 (104-148) | 7.61 (6.46-8.85) | 355 (286-428) | 9.6 (8.23-11.32) | 0.75 (0.7-0.8) |
| Pakistan | 3410 (2812-4244) | 4.2 (3.5-5.08) | 8821 (7364-10873) | 5.18 (4.38-6.15) | 0.69 (0.66-0.71) |
| Palau | 2 (1-2) | 15.23 (13.37-17.29) | 3 (3-4) | 17.84 (15.79-20.13) | 0.55 (0.53-0.56) |
| Palestine | 101 (82-127) | 5.66 (4.66-7.01) | 277 (224-348) | 6.57 (5.44-8.08) | 0.47 (0.43-0.52) |
| Panama | 197 (169-236) | 10.43 (8.89-12.23) | 735 (628-851) | 16.85 (14.39-19.54) | 1.65 (1.62-1.67) |
| Papua New Guinea | 223 (188-272) | 8.51 (7.25-9.91) | 646 (553-765) | 9.65 (8.39-10.83) | 0.41 (0.38-0.44) |
| Paraguay | 325 (276-393) | 10.51 (8.92-12.35) | 1013 (863-1196) | 15.68 (13.46-18.3) | 1.38 (1.28-1.48) |
| Peru | 1407 (1204-1673) | 8.29 (7.15-9.71) | 3780 (3159-4507) | 10.83 (9.06-12.84) | 0.87 (0.81-0.93) |
| Philippines | 4312 (3517-5355) | 10.07 (8.29-11.99) | 13534 (11540-15737) | 14.13 (12.16-16.28) | 1.16 (1.01-1.31) |
| Poland | 3254 (2668-3902) | 7.94 (6.57-9.49) | 6820 (5699-8086) | 11.8 (9.88-13.92) | 1.2 (1.04-1.37) |
| Portugal | 1218 (1006-1440) | 9.98 (8.28-11.86) | 3316 (2875-3795) | 17.03 (14.96-19.81) | 1.65 (1.45-1.85) |
| Puerto Rico | 453 (388-533) | 12.7 (10.88-14.97) | 838 (712-973) | 17.15 (14.94-20.08) | 0.97 (0.8-1.13) |
| Qatar | 22 (17-28) | 6.74 (5.63-8.19) | 196 (149-251) | 8.79 (7.43-10.53) | 0.99 (0.95-1.04) |
| Republic of Korea | 4660 (4000-5458) | 14.61 (12.95-16.41) | 10238 (8672-12111) | 13.1 (11.05-15.39) | -0.92 (-1.11--0.73) |
| Republic of Moldova | 324 (257-400) | 7.21 (5.81-8.82) | 519 (430-620) | 10.47 (8.73-12.41) | 1.43 (1.33-1.54) |
| Romania | 1883 (1486-2314) | 7.22 (5.78-8.8) | 3312 (2746-3965) | 11.23 (9.44-13.22) | 1.57 (1.43-1.71) |
| Russian Federation | 13201 (10776-15885) | 7.92 (6.56-9.46) | 31257 (26316-36597) | 16.38 (13.96-19.14) | 2.75 (2.6-2.9) |
| Rwanda | 463 (397-557) | 8.12 (7.2-9.43) | 867 (755-1043) | 8.24 (7.2-9.49) | -0.05 (-0.15-0.05) |
| Saint Kitts and Nevis | 4 (4-5) | 10.59 (9.03-12.46) | 7 (6-9) | 12.03 (10.31-14.15) | 0.3 (0.2-0.41) |
| Saint Lucia | 20 (17-23) | 17.69 (15.48-19.98) | 57 (49-64) | 26.83 (23.69-30.15) | 1.24 (1.12-1.37) |
| Saint Vincent and the Grenadines | 10 (8-12) | 10.04 (8.71-11.96) | 17 (14-19) | 13.33 (11.56-15.35) | 0.85 (0.74-0.96) |
| Samoa | 14 (12-17) | 12.88 (11.3-14.65) | 28 (25-32) | 17.52 (15.64-19.63) | 1.02 (0.97-1.07) |
| San Marino | 5 (5-6) | 16.86 (14.58-19.3) | 15 (13-17) | 22 (19.05-25.14) | 0.87 (0.78-0.95) |
| Sao Tome and Principe | 13 (11-16) | 9.7 (8.41-11.26) | 19 (16-23) | 9.98 (8.7-11.57) | 0.11 (0.04-0.18) |
| Saudi Arabia | 806 (652-1028) | 6.71 (5.63-8.14) | 2892 (2315-3521) | 9.62 (8.17-11.28) | 1.31 (1.24-1.37) |
| Senegal | 725 (621-854) | 8.81 (7.73-10.1) | 1294 (1122-1507) | 8.71 (7.72-9.99) | -0.18 (-0.21--0.14) |
| Serbia | 787 (637-957) | 7.35 (6.06-8.83) | 1434 (1194-1708) | 10.54 (8.79-12.45) | 1.4 (1.31-1.48) |
| Seychelles | 9 (8-11) | 14.75 (12.68-17.14) | 18 (16-21) | 16.1 (14.29-18.59) | 0.26 (0.19-0.33) |
| Sierra Leone | 464 (396-540) | 9.54 (8.39-10.89) | 776 (680-899) | 8.78 (7.77-10.04) | -0.35 (-0.45--0.26) |
| Singapore | 198 (175-225) | 7.35 (6.53-8.32) | 1375 (1151-1598) | 17.46 (14.86-20.29) | 2.8 (2.54-3.06) |
| Slovakia | 486 (399-590) | 8.56 (7.09-10.36) | 1124 (933-1339) | 13.82 (11.48-16.31) | 1.44 (1.37-1.51) |
| Slovenia | 184 (151-222) | 8.02 (6.61-9.61) | 416 (341-494) | 11.5 (9.51-13.63) | 1.16 (1.11-1.22) |
| Solomon Islands | 20 (17-24) | 9.12 (8.05-10.46) | 55 (48-63) | 11.94 (10.53-13.41) | 0.9 (0.88-0.93) |
| Somalia | 445 (375-542) | 7.35 (6.49-8.56) | 1056 (898-1290) | 6.25 (5.54-7.3) | -0.53 (-0.55--0.51) |
| South Africa | 2781 (2321-3386) | 8.9 (7.62-10.55) | 4267 (3608-5143) | 8.08 (6.9-9.48) | -0.46 (-0.54--0.39) |
| South Sudan | 364 (312-440) | 7.61 (6.64-8.92) | 541 (462-662) | 6.86 (6.01-8.06) | -0.4 (-0.44--0.36) |
| Spain | 4666 (3896-5474) | 9.79 (8.24-11.68) | 13476 (11862-15344) | 15.82 (13.88-18.06) | 1.37 (1.04-1.7) |
| Sri Lanka | 2185 (1883-2584) | 16.78 (14.57-19.63) | 6374 (5456-7347) | 25.15 (21.83-28.94) | 1.55 (1.46-1.64) |
| Sudan | 954 (802-1171) | 5.4 (4.59-6.5) | 2272 (1882-2796) | 6.47 (5.44-7.69) | 0.6 (0.56-0.64) |
| Suriname | 28 (23-35) | 8.28 (6.98-9.98) | 60 (51-71) | 10.05 (8.59-11.89) | 0.66 (0.58-0.75) |
| Sweden | 1124 (928-1354) | 9.67 (7.92-11.89) | 2617 (2178-3132) | 14.76 (12.42-17.35) | 1.28 (1.03-1.54) |
| Switzerland | 918 (784-1068) | 10.02 (8.48-11.81) | 3960 (3458-4429) | 22.31 (19.53-25.01) | 2.98 (2.83-3.13) |
| Syrian Arab Republic | 613 (495-772) | 5.69 (4.66-6.97) | 955 (787-1173) | 6.95 (5.83-8.38) | 0.67 (0.54-0.8) |
| Taiwan (Province of China) | 1832 (1513-2193) | 10.89 (9.1-12.86) | 9084 (7776-10549) | 23.89 (20.59-27.54) | 2.87 (2.56-3.19) |
| Tajikistan | 165 (130-209) | 4.15 (3.32-5.16) | 350 (280-444) | 4.31 (3.52-5.27) | 0.11 (0.08-0.14) |
| Thailand | 12569 (11175-14455) | 30.5 (27.06-34.45) | 31956 (27770-36611) | 33.55 (29.76-37.89) | 0.14 (0.05-0.23) |
| Timor-Leste | 47 (38-58) | 9.15 (7.58-10.92) | 116 (99-138) | 11 (9.38-12.8) | 0.75 (0.65-0.85) |
| Togo | 352 (300-416) | 9.32 (8.13-10.7) | 613 (531-719) | 8.26 (7.24-9.45) | -0.48 (-0.52--0.45) |
| Tokelau | 0 (0-0) | 13.03 (11.3-14.89) | 0 (0-0) | 16.65 (14.64-19) | 0.8 (0.78-0.82) |
| Tonga | 9 (7-10) | 12.01 (10.32-13.86) | 13 (12-15) | 15.02 (13.15-16.91) | 0.74 (0.72-0.77) |
| Trinidad and Tobago | 109 (92-133) | 10.65 (9.06-12.7) | 223 (191-258) | 13.42 (11.56-15.61) | 0.82 (0.71-0.93) |
| Tunisia | 489 (408-592) | 7.09 (5.94-8.4) | 1123 (941-1347) | 9.08 (7.71-10.81) | 0.85 (0.8-0.9) |
| Turkey | 3806 (3238-4534) | 8.36 (7.21-9.7) | 10025 (8482-11820) | 11.18 (9.53-13.15) | 0.96 (0.93-0.98) |
| Turkmenistan | 113 (88-143) | 4.06 (3.18-5.07) | 222 (181-270) | 4.8 (3.97-5.79) | 0.57 (0.56-0.59) |
| Tuvalu | 1 (1-1) | 11.98 (10.52-13.57) | 2 (1-2) | 15.56 (13.74-17.38) | 0.85 (0.8-0.9) |
| Uganda | 1100 (912-1354) | 7.5 (6.46-8.89) | 2782 (2375-3389) | 8.11 (7.09-9.48) | 0.19 (0.14-0.24) |
| Ukraine | 5280 (4328-6317) | 8.5 (7.07-10.24) | 7384 (6203-8722) | 12.53 (10.68-14.77) | 1.5 (1.35-1.64) |
| United Arab Emirates | 107 (86-134) | 7.36 (6.32-8.87) | 689 (524-892) | 8.56 (7.25-10.28) | 0.61 (0.55-0.67) |
| United Kingdom | 6561 (5398-7877) | 9.77 (8.1-12) | 19100 (16128-22438) | 17.28 (14.72-20.09) | 2.2 (2.12-2.28) |
| United Republic of Tanzania | 1655 (1401-2018) | 8.15 (7.04-9.52) | 4029 (3421-4860) | 8.8 (7.7-10.17) | 0.16 (0.11-0.22) |
| United States of America | 28369 (24089-34089) | 10.44 (8.83-12.65) | 74668 (64723-86104) | 15.7 (13.87-18.02) | 1.18 (1-1.37) |
| United States Virgin Islands | 16 (13-18) | 16.44 (14.12-19) | 29 (25-33) | 20.76 (18.28-23.85) | 0.75 (0.65-0.85) |
| Uruguay | 483 (412-555) | 13.32 (11.48-15.39) | 1105 (960-1261) | 22.29 (19.39-25.45) | 1.75 (1.62-1.87) |
| Uzbekistan | 681 (547-846) | 4.06 (3.3-5) | 1562 (1286-1897) | 5.18 (4.32-6.15) | 0.91 (0.87-0.96) |
| Vanuatu | 10 (8-12) | 9.83 (8.54-11.44) | 28 (24-32) | 12.55 (11.17-14.24) | 0.85 (0.83-0.87) |
| Venezuela (Bolivarian Republic of) | 1040 (839-1295) | 6.95 (5.71-8.45) | 2339 (1935-2809) | 8.26 (6.91-9.95) | 0.47 (0.37-0.58) |
| Viet Nam | 6782 (5778-8097) | 13.06 (11.13-15.3) | 18986 (16239-21926) | 18.89 (16.25-21.76) | 1.32 (1.24-1.41) |
| Yemen | 627 (514-782) | 5.29 (4.48-6.36) | 1636 (1371-2015) | 6.12 (5.28-7.32) | 0.58 (0.49-0.67) |
| Zambia | 433 (368-534) | 6.99 (6.1-8.17) | 1211 (1038-1461) | 8.65 (7.64-9.95) | 0.71 (0.6-0.82) |
| Zimbabwe | 525 (425-666) | 5.6 (4.61-6.89) | 730 (588-912) | 5.22 (4.37-6.43) | -0.38 (-0.45--0.3) |

**Supplementary Table 2** Data related to Prevalence

|  | Number of prevalence cases (95% UI) in 1990 | The age-standardized prevalence rate/100000 (95% UI) in 1990 | Number of prevalence cases (95% UI) in 2021 | The age-standardized prevalence rate/100000 (95% UI) in 2021 | EAPC (95% CI) |
| --- | --- | --- | --- | --- | --- |
| Global | 140559 (121658-161473) | 2.93 (2.56-3.35) | 421667 (362728-482472) | 5.31 (4.58-6.1) | 2.03 (1.9-2.16) |
| Sex |  |  |  |  |  |
| Female | 67064 (58374-76752) | 2.73 (2.4-3.11) | 208603 (180463-237916) | 4.97 (4.28-5.69) | 2.12 (1.94-2.29) |
| Male | 73495 (63637-84994) | 3.12 (2.72-3.55) | 213065 (183106-245856) | 5.66 (4.86-6.5) | 1.96 (1.87-2.04) |
| Age |  |  |  |  |  |
| <5 years | 13398 (10880-16290) | 2.16 (1.76-2.63) | 20001 (15665-25202) | 3.04 (2.38-3.83) | 1.06 (0.96-1.16) |
| 5-9 years | 13022 (8930-18765) | 2.23 (1.53-3.22) | 23855 (15447-35544) | 3.47 (2.25-5.17) | 1.49 (1.36-1.62) |
| 10-14 years | 9938 (7021-13803) | 1.86 (1.31-2.58) | 20612 (14197-29252) | 3.09 (2.13-4.39) | 1.72 (1.68-1.76) |
| 15-19 years | 13326 (9343-18765) | 2.57 (1.8-3.61) | 29857 (19472-44032) | 4.79 (3.12-7.06) | 1.94 (1.85-2.03) |
| 20-24 years | 13670 (9849-18660) | 2.78 (2-3.79) | 30132 (20554-43290) | 5.05 (3.44-7.25) | 1.82 (1.71-1.93) |
| 25-29 years | 13140 (9473-18164) | 2.97 (2.14-4.1) | 30312 (21073-43250) | 5.15 (3.58-7.35) | 1.88 (1.82-1.94) |
| 30-34 years | 9932 (6576-14498) | 2.58 (1.71-3.76) | 23799 (15316-35706) | 3.94 (2.53-5.91) | 1.64 (1.54-1.73) |
| 35-39 years | 6762 (4647-9369) | 1.92 (1.32-2.66) | 16234 (10723-23391) | 2.89 (1.91-4.17) | 1.33 (1.25-1.41) |
| 40-44 years | 3084 (2074-4426) | 1.08 (0.72-1.54) | 7633 (5096-11091) | 1.53 (1.02-2.22) | 1.01 (0.94-1.08) |
| 45-49 years | 2839 (2028-3906) | 1.22 (0.87-1.68) | 8531 (6084-11769) | 1.8 (1.28-2.49) | 1.13 (1.04-1.22) |
| 50-54 years | 2776 (1948-3831) | 1.31 (0.92-1.8) | 9228 (6671-12239) | 2.07 (1.5-2.75) | 1.47 (1.3-1.65) |
| 55-59 years | 2929 (2091-3926) | 1.58 (1.13-2.12) | 10823 (7793-14080) | 2.73 (1.97-3.56) | 1.94 (1.7-2.18) |
| 60-64 years | 4145 (3182-5313) | 2.58 (1.98-3.31) | 15687 (12125-19992) | 4.9 (3.79-6.25) | 2.21 (1.96-2.46) |
| 65-69 years | 6820 (5092-9052) | 5.52 (4.12-7.32) | 29380 (21993-37702) | 10.65 (7.97-13.67) | 2.43 (2.18-2.68) |
| 70-74 years | 6302 (4425-8586) | 7.44 (5.23-10.14) | 34844 (25074-46444) | 16.93 (12.18-22.56) | 2.7 (2.45-2.94) |
| 75-79 years | 5823 (3991-8029) | 9.46 (6.48-13.04) | 27030 (19465-36150) | 20.5 (14.76-27.41) | 2.76 (2.44-3.08) |
| 80-84 years | 5550 (3832-7611) | 15.69 (10.83-21.52) | 28212 (19963-38168) | 32.21 (22.79-43.58) | 2.61 (2.32-2.9) |
| 85-89 years | 4488 (3076-6403) | 29.7 (20.36-42.37) | 28764 (20088-39413) | 62.91 (43.94-86.2) | 2.75 (2.48-3.02) |
| 90-94 years | 1988 (1300-2893) | 46.38 (30.34-67.52) | 18649 (12847-26992) | 104.25 (71.81-150.88) | 2.88 (2.6-3.15) |
| 95+ years | 627 (345-1108) | 61.55 (33.92-108.88) | 8082 (4657-14125) | 148.29 (85.45-259.17) | 2.87 (2.59-3.16) |
| SDI region |  |  |  |  |  |
| High-middle SDI | 23324 (20558-26404) | 2.3 (2.05-2.58) | 63095 (56229-70028) | 4.37 (3.91-4.92) | 2.15 (2.03-2.26) |
| High SDI | 40871 (34827-47329) | 4.23 (3.66-4.85) | 159612 (133414-186949) | 9.81 (8.49-11.17) | 2.93 (2.68-3.18) |
| Low-middle SDI | 22140 (18868-26035) | 2.17 (1.88-2.49) | 60687 (50741-73193) | 3.32 (2.81-3.92) | 1.35 (1.28-1.41) |
| Low SDI | 13452 (10068-17863) | 2.8 (2.18-3.6) | 47420 (34796-63413) | 4.2 (3.2-5.4) | 1.4 (1.37-1.44) |
| Middle SDI | 40639 (35222-47080) | 2.55 (2.25-2.88) | 90484 (79396-103372) | 3.8 (3.33-4.35) | 1.31 (1.23-1.39) |
| GBD region |  |  |  |  |  |
| Advanced Health System | 51794 (44727-59469) | 3.68 (3.2-4.19) | 196813 (166977-227996) | 9.12 (7.95-10.39) | 3.18 (2.95-3.41) |
| Africa | 23080 (17341-30612) | 3.49 (2.67-4.47) | 71473 (51927-96241) | 4.93 (3.69-6.43) | 1.12 (1.08-1.17) |
| African Region | 21032 (15607-28199) | 3.93 (2.97-5.11) | 66157 (47412-89767) | 5.44 (4.01-7.15) | 1.06 (1.01-1.11) |
| America | 32618 (27869-38176) | 4.68 (4-5.42) | 84724 (73201-98997) | 7.51 (6.48-8.84) | 1.6 (1.38-1.82) |
| Andean Latin America | 1088 (894-1317) | 2.91 (2.47-3.41) | 3010 (2558-3556) | 4.65 (3.98-5.49) | 1.49 (1.28-1.7) |
| Asia | 56840 (49890-63996) | 2.1 (1.86-2.34) | 153511 (135680-171577) | 3.41 (3-3.81) | 1.49 (1.4-1.58) |
| Australasia | 568 (497-642) | 2.6 (2.29-2.94) | 4228 (3631-4907) | 9.41 (8.09-10.72) | 4.52 (3.94-5.1) |
| Basic Health System | 53225 (46343-61381) | 2.48 (2.21-2.8) | 111226 (98985-125609) | 3.56 (3.15-4.04) | 1.12 (1.02-1.22) |
| Caribbean | 1040 (890-1218) | 2.97 (2.58-3.4) | 2267 (1949-2585) | 4.74 (4.07-5.44) | 1.56 (1.28-1.83) |
| Central Africa | 1973 (1404-2729) | 2.83 (2.06-3.79) | 7954 (5492-11076) | 4.49 (3.18-6.1) | 1.55 (1.49-1.61) |
| Central Asia | 721 (611-849) | 1.01 (0.87-1.18) | 848 (737-987) | 0.94 (0.82-1.09) | -0.25 (-0.4--0.09) |
| Central Europe | 2498 (2180-2873) | 2.04 (1.78-2.37) | 7060 (6231-8051) | 6 (5.17-7) | 3.55 (3.11-3.99) |
| Central Latin America | 4479 (3757-5346) | 2.54 (2.2-2.97) | 11701 (10073-13554) | 4.74 (4.1-5.47) | 2.18 (1.66-2.7) |
| Central Sub-Saharan Africa | 1472 (1036-2042) | 2.58 (1.88-3.46) | 6112 (4149-8570) | 4.22 (2.97-5.77) | 1.7 (1.62-1.79) |
| Commonwealth High Income | 3483 (2983-3988) | 2.8 (2.4-3.2) | 18586 (15713-21671) | 8.82 (7.56-9.99) | 4.27 (3.91-4.63) |
| Commonwealth Low Income | 8532 (6394-11153) | 4.19 (3.28-5.26) | 24686 (18189-32580) | 6.16 (4.75-7.87) | 1.29 (1.2-1.38) |
| Commonwealth Middle Income | 21946 (18561-25987) | 2.17 (1.87-2.51) | 65825 (54030-79231) | 3.31 (2.77-3.89) | 1.38 (1.27-1.48) |
| East Asia | 17910 (15591-20528) | 1.6 (1.4-1.82) | 36462 (31765-41151) | 2.15 (1.91-2.43) | 0.71 (0.62-0.8) |
| East Asia & Pacific - WB | 38611 (33786-43350) | 2.36 (2.11-2.64) | 98293 (87046-110179) | 3.71 (3.29-4.16) | 1.29 (1.11-1.46) |
| Eastern Africa | 8770 (6295-11811) | 4.69 (3.46-6.18) | 25888 (18193-35351) | 6.06 (4.45-8.03) | 0.86 (0.81-0.91) |
| Eastern Europe | 3668 (3168-4273) | 1.63 (1.4-1.92) | 9636 (8187-11357) | 4.98 (4.13-6.04) | 3.7 (3.41-4) |
| Eastern Mediterranean Region | 6044 (5235-7068) | 1.81 (1.58-2.05) | 16837 (14561-19685) | 2.42 (2.11-2.77) | 0.98 (0.93-1.03) |
| Eastern Sub-Saharan Africa | 10106 (7124-13808) | 4.95 (3.58-6.65) | 30134 (20831-41884) | 6.37 (4.57-8.55) | 0.85 (0.79-0.9) |
| Europe | 27751 (24298-31685) | 3.21 (2.83-3.65) | 111265 (95198-129747) | 9.39 (8.2-10.67) | 3.93 (3.68-4.18) |
| Europe & Central Asia - WB | 28305 (24808-32302) | 3.12 (2.75-3.55) | 111845 (95760-130286) | 8.79 (7.66-10.01) | 3.81 (3.56-4.06) |
| European Region | 28417 (24915-32426) | 3.11 (2.74-3.54) | 112695 (96491-131246) | 8.76 (7.63-9.97) | 3.81 (3.56-4.06) |
| High-income Asia Pacific | 5141 (4416-5879) | 3.01 (2.6-3.42) | 24445 (19365-30707) | 6.82 (5.82-7.83) | 2.08 (1.58-2.57) |
| High-income North America | 17403 (14523-20919) | 5.49 (4.62-6.53) | 48667 (40229-58468) | 9.93 (8.28-11.92) | 1.99 (1.87-2.11) |
| Latin America & Caribbean - WB | 15346 (12934-18182) | 3.5 (3.04-4.05) | 36366 (31695-41873) | 5.58 (4.87-6.43) | 1.56 (1.24-1.87) |
| Limited Health System | 31222 (25835-37688) | 2.27 (1.92-2.67) | 97975 (78851-120894) | 3.53 (2.92-4.25) | 1.44 (1.35-1.53) |
| Middle East & North Africa - WB | 4175 (3610-4882) | 1.7 (1.5-1.93) | 11578 (10047-13331) | 2.68 (2.36-3.03) | 1.58 (1.51-1.65) |
| Minimal Health System | 4185 (2985-5700) | 3.04 (2.21-4.06) | 15286 (10694-20976) | 4.32 (3.09-5.81) | 1.18 (1.15-1.2) |
| North Africa and Middle East | 6673 (5718-7825) | 2.15 (1.88-2.42) | 16885 (14548-19343) | 2.92 (2.56-3.32) | 1.03 (0.93-1.12) |
| North America | 17404 (14524-20920) | 5.49 (4.62-6.53) | 48670 (40232-58471) | 9.93 (8.28-11.92) | 1.99 (1.87-2.11) |
| Northern Africa | 1920 (1618-2280) | 1.64 (1.42-1.88) | 4592 (3932-5365) | 2.29 (2.01-2.64) | 1.04 (0.99-1.09) |
| Oceania | 125 (105-146) | 2.79 (2.45-3.16) | 391 (328-463) | 3.77 (3.23-4.3) | 1.09 (1.04-1.14) |
| Region of the Americas | 32618 (27869-38176) | 4.68 (4-5.42) | 84724 (73201-98997) | 7.51 (6.48-8.84) | 1.6 (1.38-1.82) |
| South-East Asia Region | 24852 (21548-28521) | 2.49 (2.19-2.8) | 66144 (57695-75153) | 3.57 (3.13-4.03) | 1.22 (1.11-1.34) |
| South Asia | 14741 (12774-16847) | 1.76 (1.54-2.01) | 45564 (39259-52440) | 2.69 (2.33-3.06) | 1.42 (1.28-1.57) |
| South Asia - WB | 15351 (13301-17530) | 1.78 (1.56-2.03) | 47393 (40921-54449) | 2.72 (2.36-3.08) | 1.44 (1.3-1.57) |
| Southeast Asia | 15403 (13299-17836) | 4.14 (3.62-4.64) | 34227 (30148-38383) | 5.52 (4.84-6.24) | 1.01 (0.96-1.05) |
| Southern Africa | 4558 (3279-6238) | 4.41 (3.24-5.93) | 11063 (7764-15366) | 5.65 (4.12-7.67) | 1.03 (0.88-1.19) |
| Southern Latin America | 2064 (1767-2392) | 4.32 (3.72-4.99) | 4524 (3884-5300) | 5.97 (5.16-6.86) | 0.73 (0.45-1.02) |
| Southern Sub-Saharan Africa | 2967 (2192-3967) | 5.01 (3.78-6.59) | 5504 (4060-7401) | 6.63 (4.99-8.79) | 1.27 (1.04-1.5) |
| Sub-Saharan Africa - WB | 21212 (15692-28515) | 3.95 (2.97-5.15) | 67111 (47952-91204) | 5.39 (3.97-7.1) | 1.02 (0.97-1.06) |
| Tropical Latin America | 6713 (5514-8134) | 4.22 (3.56-5.02) | 14947 (12820-17750) | 6.7 (5.72-7.95) | 1.64 (1.4-1.88) |
| Western Africa | 5859 (4472-7515) | 3.37 (2.61-4.33) | 21975 (16009-29314) | 5.03 (3.72-6.59) | 1.14 (1.04-1.25) |
| Western Europe | 19355 (16556-22683) | 4.08 (3.57-4.68) | 90491 (75588-108638) | 12.46 (10.78-14.19) | 4.25 (3.93-4.58) |
| Western Pacific Region | 26916 (23675-30319) | 1.98 (1.76-2.22) | 69203 (60653-78731) | 3.1 (2.74-3.5) | 1.11 (0.86-1.36) |
| Western Sub-Saharan Africa | 6423 (4882-8257) | 3.33 (2.58-4.28) | 24564 (17960-32787) | 4.99 (3.69-6.51) | 1.15 (1.05-1.25) |
| World Bank High Income | 46170 (39417-53474) | 4.08 (3.54-4.67) | 183135 (153718-214120) | 9.92 (8.66-11.28) | 3.14 (2.89-3.4) |
| World Bank Low Income | 9201 (6679-12468) | 2.89 (2.15-3.83) | 31427 (22262-43464) | 4.27 (3.13-5.69) | 1.39 (1.35-1.43) |
| World Bank Lower Middle Income | 36869 (31605-43441) | 2.12 (1.84-2.44) | 104701 (87742-125933) | 3.24 (2.73-3.81) | 1.33 (1.25-1.41) |
| World Bank Upper Middle Income | 48185 (41973-55634) | 2.54 (2.25-2.88) | 102034 (90822-115325) | 3.95 (3.48-4.51) | 1.4 (1.28-1.53) |
| Country |  |  |  |  |  |
| Afghanistan | 100 (85-120) | 1.1 (0.95-1.26) | 458 (375-560) | 1.52 (1.3-1.77) | 1.06 (1.03-1.09) |
| Albania | 92 (77-112) | 2.8 (2.4-3.27) | 148 (127-174) | 5.51 (4.64-6.52) | 2.03 (1.9-2.17) |
| Algeria | 434 (366-517) | 1.76 (1.54-2.01) | 1127 (960-1317) | 2.67 (2.29-3.1) | 1.43 (1.35-1.5) |
| American Samoa | 1 (1-1) | 3.12 (2.72-3.57) | 3 (3-4) | 7.65 (6.46-8.88) | 3.66 (3.41-3.91) |
| Andorra | 2 (1-2) | 3.08 (2.68-3.56) | 6 (5-7) | 5.26 (4.57-6.09) | 1.85 (1.65-2.06) |
| Angola | 224 (158-306) | 2.14 (1.58-2.84) | 1255 (859-1782) | 3.72 (2.65-5.1) | 1.9 (1.85-1.96) |
| Antigua and Barbuda | 3 (3-3) | 4.73 (4.07-5.5) | 6 (5-6) | 6.56 (5.62-7.62) | 1.29 (0.89-1.69) |
| Argentina | 1590 (1351-1854) | 4.93 (4.2-5.73) | 3131 (2649-3683) | 6.23 (5.35-7.2) | 0.28 (-0.04-0.61) |
| Armenia | 25 (21-29) | 0.75 (0.64-0.88) | 30 (26-35) | 0.95 (0.82-1.12) | 1.09 (0.94-1.24) |
| Australia | 480 (418-546) | 2.63 (2.3-2.99) | 3868 (3309-4510) | 10.18 (8.77-11.65) | 4.71 (4.01-5.42) |
| Austria | 167 (147-189) | 1.63 (1.46-1.81) | 611 (516-702) | 4.28 (3.76-4.79) | 3.46 (3.09-3.83) |
| Azerbaijan | 27 (23-33) | 0.41 (0.35-0.5) | 47 (39-57) | 0.46 (0.39-0.55) | 0.52 (0.46-0.57) |
| Bahamas | 15 (12-18) | 5.68 (4.85-6.75) | 23 (19-27) | 6.25 (5.32-7.33) | 0.26 (0.17-0.35) |
| Bahrain | 11 (9-13) | 2.23 (1.93-2.53) | 46 (40-53) | 3.54 (3.11-4.05) | 2.2 (1.82-2.58) |
| Bangladesh | 1438 (1196-1674) | 2 (1.7-2.34) | 4571 (3839-5314) | 3.01 (2.6-3.48) | 1.47 (1.39-1.55) |
| Barbados | 21 (18-25) | 7.87 (6.76-9.12) | 34 (29-39) | 10 (8.59-11.57) | 0.81 (0.75-0.86) |
| Belarus | 317 (268-383) | 3.09 (2.58-3.78) | 446 (385-512) | 5.3 (4.39-6.35) | 1.8 (1.52-2.08) |
| Belgium | 329 (285-383) | 2.73 (2.41-3.12) | 2376 (2039-2880) | 13.94 (11.95-16.06) | 6.27 (5.42-7.11) |
| Belize | 3 (3-4) | 1.75 (1.52-2.01) | 10 (8-11) | 2.37 (2.06-2.71) | 1.09 (0.81-1.36) |
| Benin | 145 (109-190) | 3 (2.25-3.92) | 695 (497-936) | 5.04 (3.66-6.68) | 1.58 (1.46-1.7) |
| Bermuda | 2 (2-2) | 3.68 (3.21-4.21) | 5 (5-6) | 7.18 (6.1-8.32) | 2.32 (1.88-2.77) |
| Bhutan | 9 (7-10) | 1.98 (1.69-2.27) | 19 (16-21) | 2.57 (2.2-2.93) | 0.85 (0.72-0.97) |
| Bolivia (Plurinational State of) | 169 (138-209) | 2.6 (2.19-3.1) | 487 (402-586) | 4.16 (3.49-4.95) | 1.63 (1.43-1.84) |
| Bosnia and Herzegovina | 57 (49-66) | 1.32 (1.14-1.54) | 63 (56-72) | 1.67 (1.45-1.92) | 0.91 (0.79-1.03) |
| Botswana | 71 (50-97) | 4.63 (3.33-6.17) | 189 (132-257) | 7.46 (5.35-10.08) | 1.58 (1.52-1.64) |
| Brazil | 6409 (5251-7798) | 4.15 (3.49-4.94) | 14325 (12250-17011) | 6.61 (5.63-7.87) | 1.65 (1.4-1.9) |
| Brunei Darussalam | 5 (4-6) | 2.49 (2.14-2.94) | 14 (12-16) | 3.63 (3.11-4.18) | 1.21 (1.13-1.29) |
| Bulgaria | 136 (119-157) | 1.6 (1.38-1.86) | 235 (205-268) | 3.96 (3.34-4.7) | 3.18 (2.92-3.44) |
| Burkina Faso | 251 (189-327) | 2.71 (2.04-3.56) | 970 (693-1301) | 4.27 (3.09-5.65) | 1.43 (1.28-1.57) |
| Burundi | 240 (161-345) | 3.94 (2.7-5.64) | 832 (538-1217) | 5.61 (3.75-7.89) | 1.38 (1.29-1.46) |
| Cabo Verde | 19 (15-26) | 4.66 (3.53-6.07) | 61 (44-81) | 10.6 (7.88-13.69) | 2.53 (2.23-2.83) |
| Cambodia | 113 (97-130) | 1.63 (1.42-1.85) | 324 (281-371) | 2.17 (1.92-2.46) | 1.02 (0.97-1.07) |
| Cameroon | 308 (224-413) | 2.93 (2.17-3.9) | 1518 (1082-2073) | 4.57 (3.36-6.07) | 1.21 (1.09-1.34) |
| Canada | 1838 (1503-2200) | 6.06 (5-7.2) | 6963 (5730-8401) | 12.72 (10.62-14.82) | 2.65 (2.45-2.85) |
| Central African Republic | 52 (37-71) | 1.79 (1.34-2.39) | 147 (102-210) | 2.51 (1.82-3.42) | 1.14 (1.07-1.22) |
| Chad | 172 (128-224) | 2.85 (2.12-3.74) | 732 (534-987) | 4.15 (3.05-5.46) | 1.19 (1.14-1.24) |
| Chile | 265 (227-304) | 2.09 (1.82-2.38) | 971 (848-1118) | 4.63 (4.05-5.25) | 2.85 (2.48-3.22) |
| China | 17216 (14977-19733) | 1.58 (1.39-1.81) | 30795 (26531-35308) | 1.87 (1.65-2.15) | 0.09 (-0.07-0.25) |
| Colombia | 1167 (956-1415) | 3.42 (2.87-4.05) | 4161 (3522-4832) | 8.52 (7.27-9.93) | 3.14 (2.39-3.89) |
| Comoros | 30 (20-43) | 5.84 (3.99-8.15) | 59 (39-84) | 7.27 (4.98-10.18) | 0.7 (0.66-0.74) |
| Congo | 67 (48-93) | 2.53 (1.87-3.4) | 238 (163-336) | 4.24 (3.05-5.74) | 1.85 (1.8-1.91) |
| Cook Islands | 1 (1-1) | 6.19 (5.32-7.29) | 2 (2-2) | 11.74 (9.8-14.49) | 2.18 (2.11-2.26) |
| Costa Rica | 183 (152-220) | 5.93 (5.04-6.92) | 476 (412-545) | 9.72 (8.38-11.11) | 1.92 (1.69-2.16) |
| Croatia | 69 (60-80) | 1.44 (1.24-1.66) | 152 (134-171) | 3.51 (3-4.1) | 3.08 (2.77-3.38) |
| Cuba | 247 (213-283) | 2.3 (2.01-2.62) | 472 (416-529) | 4.01 (3.52-4.59) | 2.03 (1.63-2.43) |
| Cyprus | 33 (28-39) | 4.38 (3.79-5.05) | 106 (89-124) | 6.86 (5.9-7.95) | 1.49 (1.35-1.64) |
| Czechia | 302 (258-351) | 3.14 (2.68-3.68) | 1199 (1030-1390) | 12.81 (10.75-15.28) | 4.9 (4.33-5.48) |
| Côte d'Ivoire | 430 (318-559) | 3.38 (2.53-4.35) | 1534 (1116-2057) | 5.33 (3.92-7.03) | 1.48 (1.43-1.54) |
| Democratic People's Republic of Korea | 285 (246-328) | 1.52 (1.32-1.75) | 456 (401-519) | 1.86 (1.62-2.14) | 0.55 (0.44-0.65) |
| Democratic Republic of the Congo | 1088 (763-1522) | 2.74 (1.99-3.71) | 4302 (2886-6080) | 4.48 (3.13-6.12) | 1.69 (1.59-1.79) |
| Denmark | 91 (80-103) | 1.47 (1.3-1.65) | 654 (538-802) | 7.62 (6.56-8.83) | 6.42 (5.66-7.18) |
| Djibouti | 28 (18-41) | 5.75 (3.97-8.22) | 92 (59-129) | 6.81 (4.58-9.44) | 0.59 (0.52-0.67) |
| Dominica | 3 (3-4) | 4.47 (3.85-5.15) | 3 (3-3) | 4.37 (3.83-4.98) | -0.24 (-0.31--0.17) |
| Dominican Republic | 180 (150-216) | 2.5 (2.15-2.92) | 438 (371-513) | 4.04 (3.44-4.72) | 1.27 (1.08-1.46) |
| Ecuador | 176 (146-212) | 1.74 (1.49-2.03) | 782 (664-937) | 4.4 (3.76-5.23) | 2.68 (2.28-3.08) |
| Egypt | 600 (506-704) | 1.21 (1.06-1.39) | 1485 (1274-1753) | 1.52 (1.33-1.74) | 0.46 (0.36-0.56) |
| El Salvador | 63 (54-75) | 1.22 (1.07-1.42) | 106 (92-121) | 1.64 (1.43-1.87) | 0.89 (0.8-0.98) |
| Equatorial Guinea | 10 (7-13) | 2.21 (1.66-2.94) | 83 (55-117) | 4.89 (3.46-6.59) | 2.99 (2.75-3.23) |
| Eritrea | 152 (101-220) | 3.92 (2.67-5.61) | 393 (256-559) | 5.26 (3.58-7.28) | 0.95 (0.89-1.01) |
| Estonia | 58 (49-69) | 3.8 (3.17-4.56) | 106 (91-126) | 8.72 (7.05-10.83) | 2.75 (2.47-3.02) |
| Eswatini | 37 (26-51) | 3.95 (2.83-5.29) | 72 (51-100) | 5.53 (4.02-7.4) | 0.98 (0.85-1.11) |
| Ethiopia | 959 (718-1323) | 2.02 (1.59-2.65) | 3635 (2571-5183) | 3.34 (2.54-4.45) | 2 (1.86-2.14) |
| Fiji | 19 (16-22) | 3.29 (2.86-3.72) | 40 (34-46) | 5.14 (4.44-5.8) | 1.93 (1.72-2.14) |
| Finland | 57 (50-67) | 0.99 (0.86-1.16) | 230 (197-271) | 2.87 (2.51-3.28) | 3.91 (3.42-4.41) |
| France | 9300 (7592-11553) | 12.19 (10.16-14.6) | 37118 (29281-46587) | 29.54 (24.76-35.05) | 3.38 (3.16-3.6) |
| Gabon | 33 (24-46) | 3.15 (2.32-4.21) | 85 (59-119) | 4.5 (3.26-6.08) | 1.16 (1.11-1.2) |
| Gambia | 36 (26-48) | 3.49 (2.58-4.63) | 154 (111-207) | 5.91 (4.31-7.87) | 1.67 (1.54-1.8) |
| Georgia | 69 (59-80) | 1.27 (1.09-1.5) | 60 (52-68) | 1.62 (1.38-1.89) | 0.99 (0.56-1.42) |
| Germany | 2181 (1930-2487) | 2.25 (2.01-2.55) | 9808 (8135-11766) | 7.69 (6.73-8.82) | 4.96 (4.26-5.67) |
| Ghana | 910 (715-1151) | 6.17 (4.96-7.69) | 2872 (2099-3786) | 8.37 (6.24-10.92) | 0.93 (0.87-1) |
| Greece | 343 (293-401) | 2.81 (2.44-3.24) | 591 (496-702) | 3.76 (3.26-4.3) | 0.79 (0.64-0.93) |
| Greenland | 1 (1-1) | 1.53 (1.28-1.83) | 2 (1-2) | 3.22 (2.61-3.83) | 2.67 (2.32-3.02) |
| Grenada | 6 (5-7) | 6.37 (5.45-7.35) | 8 (7-9) | 8.05 (6.97-9.15) | 0.49 (0.27-0.72) |
| Guam | 3 (2-3) | 2.58 (2.26-2.92) | 9 (8-10) | 5.5 (4.75-6.41) | 3.35 (3.03-3.68) |
| Guatemala | 254 (201-324) | 2.49 (2.08-3.04) | 501 (414-609) | 3.21 (2.71-3.81) | 0.51 (0.16-0.85) |
| Guinea | 181 (136-233) | 3.09 (2.3-4.05) | 672 (481-895) | 4.87 (3.54-6.43) | 1.37 (1.3-1.45) |
| Guinea-Bissau | 25 (19-32) | 2.37 (1.8-3.08) | 93 (66-122) | 4.18 (3.02-5.45) | 1.82 (1.71-1.94) |
| Guyana | 14 (12-16) | 1.77 (1.55-2.05) | 46 (38-55) | 6.17 (5.2-7.37) | 3.1 (2.46-3.75) |
| Haiti | 153 (127-182) | 2.73 (2.32-3.15) | 480 (397-573) | 4.04 (3.38-4.68) | 1.29 (1.14-1.43) |
| Honduras | 79 (67-95) | 1.77 (1.55-2.03) | 276 (230-333) | 2.79 (2.37-3.25) | 1.61 (1.55-1.67) |
| Hungary | 236 (206-272) | 2.34 (2-2.73) | 692 (604-797) | 7.44 (6.33-8.89) | 4.19 (3.72-4.67) |
| Iceland | 3 (2-3) | 1.02 (0.87-1.18) | 15 (13-17) | 3.59 (3.1-4.08) | 4.66 (4.04-5.28) |
| India | 11371 (9829-13016) | 1.7 (1.47-1.93) | 35565 (30322-41168) | 2.71 (2.33-3.1) | 1.55 (1.39-1.71) |
| Indonesia | 3302 (2825-3868) | 2.21 (1.92-2.52) | 6936 (5905-8196) | 2.78 (2.4-3.22) | 0.46 (0.38-0.54) |
| Iran (Islamic Republic of) | 1030 (878-1210) | 1.89 (1.65-2.18) | 2217 (1897-2609) | 2.78 (2.41-3.25) | 1.36 (1.22-1.5) |
| Iraq | 234 (202-271) | 1.41 (1.24-1.58) | 749 (639-876) | 1.95 (1.71-2.23) | 1.12 (1.07-1.16) |
| Ireland | 42 (36-48) | 1.11 (0.96-1.29) | 316 (268-370) | 5.24 (4.53-6.09) | 5.84 (5.28-6.41) |
| Israel | 84 (74-95) | 1.71 (1.52-1.93) | 750 (637-895) | 6.75 (5.8-7.84) | 5.26 (4.71-5.82) |
| Italy | 3016 (2497-3627) | 4.92 (4.21-5.77) | 10336 (8135-12926) | 11.82 (10.06-13.85) | 3.46 (3.26-3.65) |
| Jamaica | 159 (131-189) | 6.42 (5.46-7.49) | 239 (203-277) | 8.46 (7.17-9.81) | 0.94 (0.81-1.07) |
| Japan | 3048 (2590-3578) | 2.28 (1.96-2.63) | 19000 (14270-24996) | 6.26 (5.21-7.33) | 2.52 (1.88-3.16) |
| Jordan | 235 (193-291) | 5.88 (5.01-6.94) | 835 (689-1001) | 7.03 (5.99-8.26) | 0.7 (0.58-0.83) |
| Kazakhstan | 217 (181-259) | 1.32 (1.12-1.57) | 204 (178-237) | 1.14 (1-1.34) | -0.91 (-1.36--0.46) |
| Kenya | 2937 (2221-3747) | 11.67 (9-15.24) | 7236 (5302-9469) | 12.83 (9.68-16.75) | -0.1 (-0.35-0.14) |
| Kiribati | 2 (1-2) | 3.35 (2.95-3.83) | 4 (3-4) | 4.19 (3.66-4.74) | 0.72 (0.68-0.76) |
| Kuwait | 25 (22-30) | 1.58 (1.38-1.8) | 124 (106-142) | 3.32 (2.87-3.8) | 2.9 (1.99-3.81) |
| Kyrgyzstan | 31 (27-37) | 0.7 (0.6-0.81) | 55 (47-66) | 0.85 (0.73-1) | 0.87 (0.7-1.04) |
| Lao People's Democratic Republic | 46 (39-52) | 1.51 (1.32-1.7) | 156 (131-184) | 2.36 (2.03-2.69) | 1.7 (1.59-1.81) |
| Latvia | 53 (46-61) | 1.92 (1.67-2.26) | 75 (66-86) | 3.81 (3.23-4.53) | 2.4 (2.02-2.77) |
| Lebanon | 111 (95-129) | 3.9 (3.4-4.45) | 315 (273-362) | 5.7 (4.96-6.6) | 1.13 (1.01-1.26) |
| Lesotho | 62 (44-84) | 3.68 (2.64-4.9) | 98 (69-136) | 4.7 (3.39-6.34) | 0.55 (0.44-0.66) |
| Liberia | 84 (62-110) | 3.39 (2.5-4.44) | 326 (228-435) | 5.73 (4.1-7.49) | 1.91 (1.8-2.01) |
| Libya | 92 (78-110) | 2.07 (1.8-2.39) | 137 (117-160) | 2.29 (1.98-2.67) | 0.11 (0-0.22) |
| Lithuania | 79 (67-93) | 2.1 (1.78-2.5) | 157 (135-185) | 6.16 (5.11-7.52) | 3.54 (3.19-3.88) |
| Luxembourg | 13 (11-15) | 3.17 (2.74-3.67) | 142 (119-173) | 19.02 (16.36-22.21) | 6.56 (5.53-7.6) |
| Madagascar | 696 (461-996) | 5.28 (3.62-7.33) | 2512 (1622-3580) | 7.52 (5.06-10.66) | 0.96 (0.88-1.03) |
| Malawi | 438 (292-636) | 4.09 (2.8-5.82) | 1320 (859-1901) | 5.77 (3.91-8.17) | 1.26 (1.15-1.36) |
| Malaysia | 922 (777-1079) | 5.75 (4.93-6.58) | 2368 (1992-2743) | 7.74 (6.63-8.93) | 0.84 (0.71-0.96) |
| Maldives | 3 (3-4) | 2.06 (1.82-2.34) | 16 (14-19) | 3.33 (2.91-3.74) | 1.68 (1.59-1.77) |
| Mali | 209 (158-273) | 2.57 (1.93-3.4) | 935 (668-1254) | 4.06 (2.95-5.46) | 1.35 (1.16-1.53) |
| Malta | 7 (6-7) | 1.75 (1.55-1.98) | 60 (51-71) | 10.34 (8.81-12.22) | 6.57 (5.9-7.24) |
| Marshall Islands | 1 (1-1) | 2.7 (2.38-3.07) | 2 (1-2) | 3.85 (3.33-4.38) | 1.24 (1.17-1.3) |
| Mauritania | 79 (58-103) | 3.76 (2.79-4.89) | 323 (233-435) | 7.06 (5.19-9.39) | 2 (1.84-2.15) |
| Mauritius | 12 (10-14) | 1.22 (1.05-1.4) | 49 (43-55) | 3.58 (3.13-4.07) | 4.29 (3.35-5.25) |
| Mexico | 2172 (1814-2613) | 2.29 (1.96-2.7) | 4821 (4041-5733) | 3.86 (3.26-4.55) | 1.98 (1.41-2.55) |
| Micronesia (Federated States of) | 2 (2-2) | 2.46 (2.13-2.8) | 3 (3-4) | 3.97 (3.48-4.52) | 1.68 (1.63-1.72) |
| Monaco | 4 (3-5) | 7.36 (6.35-8.58) | 8 (6-10) | 10.51 (9.01-12.35) | 1.2 (1.03-1.37) |
| Mongolia | 18 (15-22) | 0.87 (0.75-1.02) | 30 (25-35) | 0.98 (0.85-1.15) | 0.6 (0.51-0.7) |
| Montenegro | 7 (6-9) | 1.22 (1.05-1.42) | 11 (9-13) | 1.6 (1.37-1.85) | 0.99 (0.87-1.11) |
| Morocco | 531 (439-642) | 1.99 (1.7-2.33) | 1080 (909-1272) | 3.06 (2.59-3.61) | 1.48 (1.43-1.52) |
| Mozambique | 565 (372-801) | 4.23 (2.87-5.96) | 1750 (1155-2545) | 5.14 (3.5-7.19) | 0.6 (0.55-0.64) |
| Myanmar | 539 (461-624) | 1.71 (1.49-1.92) | 1189 (1027-1357) | 2.28 (1.99-2.58) | 1 (0.95-1.05) |
| Namibia | 72 (50-100) | 4.53 (3.28-6.09) | 187 (128-260) | 7.03 (4.98-9.59) | 1.48 (1.39-1.56) |
| Nauru | 0 (0-0) | 2.32 (2.02-2.61) | 0 (0-0) | 3.11 (2.7-3.56) | 1.09 (0.94-1.24) |
| Nepal | 243 (206-282) | 1.69 (1.44-1.93) | 752 (632-887) | 2.6 (2.22-2.99) | 1.43 (1.36-1.5) |
| Netherlands | 525 (458-603) | 3.02 (2.66-3.44) | 5385 (4394-6589) | 20.64 (17.55-23.86) | 6.94 (5.93-7.96) |
| New Zealand | 88 (75-101) | 2.44 (2.08-2.8) | 360 (296-429) | 5.4 (4.52-6.35) | 3.02 (2.47-3.57) |
| Nicaragua | 145 (118-180) | 3.62 (3.05-4.23) | 326 (270-391) | 4.96 (4.2-5.89) | 1.11 (1.05-1.16) |
| Niger | 220 (162-288) | 2.83 (2.09-3.75) | 1119 (808-1481) | 4.53 (3.31-5.98) | 1.44 (1.27-1.61) |
| Nigeria | 2780 (2094-3693) | 3.1 (2.37-4.07) | 10296 (7305-14217) | 4.45 (3.29-5.98) | 0.87 (0.71-1.04) |
| Niue | 0 (0-0) | 3.48 (3.04-3.96) | 0 (0-0) | 4.78 (4.12-5.48) | 1.12 (1.07-1.17) |
| North Macedonia | 22 (19-26) | 1.14 (0.98-1.32) | 41 (36-48) | 1.87 (1.61-2.18) | 1.85 (1.61-2.08) |
| Northern Mariana Islands | 1 (1-1) | 2.69 (2.37-3.05) | 2 (1-2) | 4.18 (3.62-4.79) | 1.79 (1.68-1.89) |
| Norway | 95 (78-112) | 1.74 (1.48-2) | 595 (443-772) | 6.29 (4.99-7.76) | 4.92 (4.48-5.35) |
| Oman | 22 (20-26) | 1.2 (1.06-1.35) | 78 (66-93) | 1.74 (1.53-1.98) | 0.98 (0.82-1.13) |
| Pakistan | 1680 (1460-1925) | 2 (1.71-2.3) | 4658 (3970-5473) | 2.32 (1.99-2.65) | 0.5 (0.37-0.63) |
| Palau | 0 (0-0) | 3.16 (2.79-3.57) | 1 (1-1) | 4.3 (3.74-4.91) | 1.01 (0.96-1.05) |
| Palestine | 18 (16-21) | 0.93 (0.82-1.06) | 57 (49-67) | 1.17 (1.03-1.33) | 0.77 (0.66-0.87) |
| Panama | 107 (90-128) | 4.69 (4.03-5.41) | 352 (304-404) | 8.15 (7.04-9.36) | 1.71 (1.57-1.84) |
| Papua New Guinea | 71 (59-84) | 2.69 (2.31-3.08) | 270 (222-322) | 3.57 (3.06-4.12) | 0.93 (0.9-0.97) |
| Paraguay | 305 (247-377) | 7.01 (5.78-8.38) | 621 (509-739) | 8.71 (7.28-10.27) | 0.95 (0.88-1.02) |
| Peru | 743 (605-903) | 3.52 (2.97-4.13) | 1741 (1481-2055) | 4.92 (4.22-5.78) | 1.13 (0.92-1.34) |
| Philippines | 954 (819-1105) | 1.94 (1.68-2.22) | 2644 (2269-3125) | 2.61 (2.27-3.02) | 0.88 (0.84-0.93) |
| Poland | 979 (849-1129) | 2.64 (2.28-3.06) | 3343 (2800-4048) | 8.01 (6.76-9.71) | 3.34 (2.65-4.03) |
| Portugal | 230 (203-259) | 2.05 (1.81-2.3) | 1831 (1483-2247) | 10.15 (8.72-11.82) | 5.91 (5.3-6.51) |
| Puerto Rico | 125 (108-144) | 3.48 (3.02-4.01) | 300 (259-342) | 7.77 (6.72-8.94) | 3.02 (2.36-3.69) |
| Qatar | 9 (7-10) | 1.99 (1.75-2.25) | 105 (85-127) | 3.77 (3.28-4.31) | 2.57 (2.34-2.8) |
| Republic of Korea | 2046 (1730-2379) | 6 (5.06-7.02) | 5115 (4439-5896) | 8.21 (7.13-9.27) | 0.77 (0.64-0.91) |
| Republic of Moldova | 82 (68-98) | 1.84 (1.54-2.23) | 166 (137-198) | 5.63 (4.51-6.91) | 3.99 (3.74-4.25) |
| Romania | 259 (221-298) | 1.08 (0.92-1.24) | 519 (454-592) | 2.64 (2.25-3.13) | 3.06 (2.91-3.22) |
| Russian Federation | 2165 (1852-2519) | 1.46 (1.24-1.72) | 7494 (6347-8916) | 5.5 (4.53-6.75) | 4.25 (3.95-4.56) |
| Rwanda | 353 (235-508) | 4.46 (3.04-6.37) | 943 (610-1397) | 6.35 (4.32-9.14) | 1.55 (1.42-1.69) |
| Saint Kitts and Nevis | 1 (1-1) | 1.88 (1.65-2.16) | 2 (1-2) | 2.75 (2.4-3.15) | 1.28 (0.93-1.63) |
| Saint Lucia | 7 (6-8) | 5.28 (4.61-6.05) | 17 (14-19) | 9.16 (7.93-10.51) | 1.82 (1.33-2.31) |
| Saint Vincent and the Grenadines | 2 (2-2) | 1.83 (1.61-2.1) | 4 (4-5) | 3.84 (3.3-4.46) | 1.99 (1.43-2.54) |
| Samoa | 5 (4-5) | 3.43 (2.97-3.91) | 9 (8-10) | 4.83 (4.2-5.53) | 1.18 (1.13-1.22) |
| San Marino | 2 (1-2) | 5.73 (4.99-6.58) | 5 (4-6) | 9.14 (7.91-10.57) | 1.81 (1.63-2) |
| Sao Tome and Principe | 5 (4-6) | 3.8 (2.75-4.98) | 15 (11-20) | 6.27 (4.59-8.22) | 1.87 (1.7-2.03) |
| Saudi Arabia | 203 (172-236) | 1.43 (1.25-1.61) | 689 (591-797) | 2.25 (1.97-2.59) | 1.83 (1.67-1.98) |
| Senegal | 319 (237-411) | 4.16 (3.1-5.49) | 1311 (951-1732) | 7.85 (5.78-10.26) | 2.08 (1.9-2.25) |
| Serbia | 150 (131-175) | 1.56 (1.34-1.8) | 224 (197-258) | 2.28 (1.96-2.66) | 1.32 (1.28-1.36) |
| Seychelles | 2 (1-2) | 2.35 (2.07-2.68) | 3 (3-3) | 2.82 (2.48-3.21) | 0.6 (0.52-0.68) |
| Sierra Leone | 127 (95-164) | 2.93 (2.18-3.83) | 476 (338-642) | 5.03 (3.66-6.66) | 1.68 (1.6-1.76) |
| Singapore | 43 (37-49) | 1.64 (1.43-1.86) | 317 (279-359) | 5.13 (4.51-5.81) | 2.63 (2.04-3.23) |
| Slovakia | 119 (102-139) | 2.31 (1.96-2.7) | 225 (199-254) | 4.24 (3.67-4.95) | 2.13 (1.92-2.34) |
| Slovenia | 29 (26-33) | 1.4 (1.24-1.61) | 104 (88-123) | 3.89 (3.37-4.51) | 3.53 (3.28-3.77) |
| Solomon Islands | 4 (4-5) | 1.93 (1.67-2.2) | 14 (12-17) | 2.73 (2.36-3.09) | 1.2 (1.17-1.23) |
| Somalia | 348 (229-501) | 4.03 (2.73-5.81) | 1217 (810-1790) | 4.91 (3.37-7.06) | 0.69 (0.67-0.7) |
| South Africa | 2545 (1865-3437) | 6.08 (4.57-8.05) | 4666 (3465-6243) | 8.13 (6.11-10.83) | 1.36 (1.1-1.62) |
| South Sudan | 313 (204-460) | 4.78 (3.22-6.77) | 495 (325-709) | 4.74 (3.23-6.67) | -0.03 (-0.05--0.01) |
| Spain | 1143 (968-1335) | 2.47 (2.12-2.84) | 8645 (7485-9978) | 10.94 (9.61-12.27) | 5.12 (4.45-5.8) |
| Sri Lanka | 506 (439-579) | 3.47 (3.06-3.89) | 1355 (1188-1523) | 5.89 (5.15-6.63) | 2.33 (2.11-2.55) |
| Sudan | 265 (224-316) | 1.41 (1.22-1.62) | 865 (710-1054) | 1.97 (1.68-2.31) | 1.06 (1-1.12) |
| Suriname | 8 (7-9) | 2.05 (1.76-2.36) | 16 (13-18) | 2.77 (2.38-3.24) | 1.01 (0.91-1.1) |
| Sweden | 515 (426-623) | 4.67 (3.98-5.4) | 1614 (1223-2140) | 8.82 (7.17-10.88) | 1.95 (1.84-2.07) |
| Switzerland | 260 (221-302) | 3.28 (2.83-3.8) | 2460 (1962-3088) | 17.92 (15.21-21.02) | 6.84 (6.25-7.44) |
| Syrian Arab Republic | 100 (86-116) | 0.84 (0.74-0.95) | 144 (124-169) | 1.07 (0.94-1.23) | 0.69 (0.54-0.85) |
| Taiwan (Province of China) | 409 (357-466) | 2.31 (2.05-2.6) | 5211 (4323-6357) | 16.17 (13.72-18.93) | 7.95 (7.18-8.72) |
| Tajikistan | 21 (18-26) | 0.45 (0.37-0.54) | 54 (45-65) | 0.56 (0.48-0.67) | 0.99 (0.9-1.09) |
| Thailand | 7142 (6007-8367) | 15.05 (12.99-17.26) | 15244 (13043-17729) | 19.39 (16.63-22.15) | 1.1 (0.99-1.21) |
| Timor-Leste | 13 (10-15) | 2.37 (2.04-2.71) | 41 (34-49) | 3.4 (2.8-3.99) | 1.35 (1.14-1.57) |
| Togo | 123 (91-163) | 3.24 (2.39-4.2) | 461 (330-614) | 5.3 (3.87-6.98) | 1.51 (1.43-1.59) |
| Tokelau | 0 (0-0) | 3.39 (2.93-3.87) | 0 (0-0) | 5.07 (4.35-5.85) | 1.33 (1.3-1.35) |
| Tonga | 4 (3-5) | 4.76 (4.05-5.49) | 6 (5-7) | 6.35 (5.36-7.41) | 0.95 (0.9-1) |
| Trinidad and Tobago | 51 (43-61) | 4.09 (3.5-4.81) | 82 (70-94) | 6 (5.08-7.07) | 1.24 (1.14-1.34) |
| Tunisia | 183 (154-217) | 2.21 (1.91-2.55) | 441 (379-511) | 3.85 (3.28-4.51) | 1.99 (1.88-2.11) |
| Turkey | 2248 (1916-2632) | 4.3 (3.71-4.95) | 4872 (4196-5591) | 5.86 (5.04-6.75) | 1.02 (0.84-1.21) |
| Turkmenistan | 23 (20-27) | 0.63 (0.55-0.73) | 42 (37-49) | 0.87 (0.76-1) | 0.94 (0.71-1.17) |
| Tuvalu | 0 (0-0) | 2.55 (2.21-2.9) | 0 (0-1) | 4.02 (3.45-4.6) | 1.47 (1.44-1.51) |
| Uganda | 1286 (871-1814) | 6.59 (4.56-9.15) | 4081 (2696-5745) | 8.21 (5.57-11.26) | 0.97 (0.85-1.1) |
| Ukraine | 915 (778-1071) | 1.7 (1.44-2.01) | 1192 (1012-1405) | 2.77 (2.29-3.38) | 2 (1.77-2.24) |
| United Arab Emirates | 48 (40-57) | 2.8 (2.43-3.19) | 341 (277-428) | 3.76 (3.25-4.32) | 1.02 (0.95-1.09) |
| United Kingdom | 897 (775-1027) | 1.38 (1.19-1.6) | 6750 (5575-8103) | 6.84 (5.87-7.91) | 6.43 (5.81-7.06) |
| United Republic of Tanzania | 1389 (898-2019) | 4.96 (3.3-6.96) | 4310 (2807-6196) | 6.6 (4.54-9.12) | 1.18 (1.1-1.25) |
| United States of America | 15564 (12837-18830) | 5.43 (4.56-6.47) | 41702 (34139-50695) | 9.59 (7.88-11.68) | 1.89 (1.78-2) |
| United States Virgin Islands | 6 (5-6) | 5.5 (4.68-6.44) | 7 (6-8) | 8.02 (6.87-9.48) | 1.34 (1.16-1.52) |
| Uruguay | 210 (179-243) | 6.31 (5.42-7.31) | 422 (343-506) | 9.58 (8.09-11.2) | 1.57 (1.44-1.7) |
| Uzbekistan | 289 (240-351) | 1.21 (1.02-1.44) | 325 (273-382) | 1 (0.85-1.18) | -0.54 (-0.94--0.15) |
| Vanuatu | 3 (2-3) | 2.38 (2.07-2.68) | 8 (7-10) | 3.23 (2.81-3.69) | 1.09 (1.03-1.14) |
| Venezuela (Bolivarian Republic of) | 308 (254-369) | 1.6 (1.37-1.86) | 682 (590-781) | 2.67 (2.28-3.08) | 1.62 (1.39-1.84) |
| Viet Nam | 1827 (1559-2117) | 3.08 (2.69-3.5) | 3853 (3369-4358) | 4 (3.52-4.5) | 0.73 (0.66-0.79) |
| Yemen | 171 (142-205) | 1.37 (1.18-1.59) | 706 (581-852) | 2.11 (1.8-2.45) | 1.49 (1.42-1.55) |
| Zambia | 365 (238-531) | 4.16 (2.78-5.83) | 1235 (795-1815) | 5.59 (3.79-7.83) | 1.1 (0.97-1.22) |
| Zimbabwe | 180 (145-226) | 1.58 (1.29-1.96) | 291 (218-385) | 1.71 (1.32-2.18) | -0.02 (-0.19-0.14) |

**Supplementary Table 3** Data related to Deaths

|  | Number of deaths cases (95% UI) in 1990 | The age-standardized deaths rate/100000 (95% UI) in 1990 | Number of deaths cases (95% UI) in 2021 | The age-standardized deaths rate/100000 (95% UI) in 2021 | EAPC (95% CI) |
| --- | --- | --- | --- | --- | --- |
| Global | 36883 (31646-40522) | 0.97 (0.84-1.05) | 77844 (69010-86338) | 0.96 (0.85-1.07) | 0.06 (-0.1-0.22) |
| Sex |  |  |  |  |  |
| Female | 17411 (14247-19925) | 0.85 (0.71-0.96) | 37750 (31155-43488) | 0.83 (0.69-0.96) | 0.02 (-0.18-0.23) |
| Male | 19473 (15931-22700) | 1.09 (0.93-1.27) | 40094 (35274-45551) | 1.1 (0.97-1.24) | 0.08 (-0.02-0.19) |
| Age |  |  |  |  |  |
| <5 years | 269701 (136344-364955) | 43.5 (21.99-58.87) | 128161 (84444-164724) | 19.47 (12.83-25.03) | -2.47 (-2.56--2.37) |
| 5-9 years | 31905 (18915-40966) | 5.47 (3.24-7.02) | 26162 (18174-32172) | 3.81 (2.65-4.68) | -1 (-1.12--0.88) |
| 10-14 years | 34040 (22181-42354) | 6.35 (4.14-7.91) | 38272 (27113-46275) | 5.74 (4.07-6.94) | -0.21 (-0.28--0.13) |
| 15-19 years | 54508 (38213-65396) | 10.49 (7.36-12.59) | 63348 (46554-77126) | 10.15 (7.46-12.36) | -0.24 (-0.35--0.14) |
| 20-24 years | 69456 (49944-79949) | 14.11 (10.15-16.25) | 87330 (66476-101310) | 14.62 (11.13-16.97) | -0.14 (-0.32-0.03) |
| 25-29 years | 80322 (59782-93163) | 18.15 (13.51-21.05) | 110328 (84685-128576) | 18.75 (14.39-21.85) | 0.05 (-0.13-0.23) |
| 30-34 years | 77992 (60739-93674) | 20.24 (15.76-24.3) | 122801 (100567-143777) | 20.32 (16.64-23.79) | 0.13 (0.03-0.23) |
| 35-39 years | 80137 (61219-93140) | 22.75 (17.38-26.44) | 130101 (103571-152468) | 23.2 (18.47-27.18) | 0.01 (-0.1-0.12) |
| 40-44 years | 76486 (59783-91716) | 26.7 (20.87-32.01) | 133919 (108336-163082) | 26.77 (21.66-32.6) | -0.26 (-0.43--0.09) |
| 45-49 years | 68247 (53681-81593) | 29.39 (23.12-35.14) | 126786 (104310-153395) | 26.78 (22.03-32.4) | -0.45 (-0.61--0.29) |
| 50-54 years | 68536 (56255-82649) | 32.24 (26.46-38.88) | 126926 (109874-152195) | 28.53 (24.7-34.21) | -0.48 (-0.61--0.35) |
| 55-59 years | 73551 (60723-88247) | 39.71 (32.79-47.65) | 142591 (122257-171759) | 36.03 (30.89-43.4) | -0.29 (-0.43--0.15) |
| 60-64 years | 72681 (59940-85068) | 45.25 (37.32-52.97) | 139313 (119982-161244) | 43.53 (37.49-50.38) | -0.3 (-0.44--0.16) |
| 65-69 years | 70851 (61011-82957) | 57.32 (49.36-67.11) | 143056 (125256-166071) | 51.86 (45.41-60.21) | -0.27 (-0.42--0.13) |
| 70-74 years | 61002 (51850-71200) | 72.05 (61.24-84.1) | 138354 (122828-157976) | 67.21 (59.67-76.75) | -0.24 (-0.37--0.12) |
| 75-79 years | 57273 (50697-65707) | 93.04 (82.36-106.74) | 117385 (104233-134167) | 89.01 (79.03-101.73) | -0.22 (-0.43--0.02) |
| 80-84 years | 43293 (37765-49423) | 122.38 (106.75-139.71) | 107120 (90654-122300) | 122.31 (103.51-139.64) | 0.11 (-0.14-0.37) |
| 85-89 years | 27459 (23549-31167) | 181.71 (155.84-206.26) | 92973 (75310-103495) | 203.34 (164.71-226.36) | 0.7 (0.42-0.98) |
| 90-94 years | 12281 (9827-13847) | 286.59 (229.33-323.13) | 67377 (51374-75665) | 376.63 (287.17-422.96) | 1.24 (0.96-1.52) |
| 95+ years | 4141 (3161-4752) | 406.75 (310.51-466.74) | 34110 (23994-39352) | 625.84 (440.24-722.01) | 1.59 (1.39-1.8) |
| SDI region |  |  |  |  |  |
| High-middle SDI | 5656 (4984-6183) | 0.63 (0.56-0.69) | 12498 (11240-13379) | 0.7 (0.63-0.75) | 0.43 (0.25-0.61) |
| High SDI | 11760 (10768-12355) | 1.1 (1-1.15) | 31485 (26952-34018) | 1.34 (1.18-1.44) | 0.8 (0.55-1.05) |
| Low-middle SDI | 6900 (5268-8220) | 0.98 (0.76-1.19) | 12138 (9926-14664) | 0.83 (0.68-1.01) | -0.51 (-0.55--0.47) |
| Low SDI | 4163 (2595-5308) | 1.55 (1-2.02) | 7782 (5220-10127) | 1.3 (0.89-1.68) | -0.59 (-0.67--0.5) |
| Middle SDI | 8371 (6769-9820) | 0.74 (0.61-0.9) | 13865 (12122-17626) | 0.56 (0.49-0.71) | -1.04 (-1.14--0.95) |
| GBD region |  |  |  |  |  |
| Advanced Health System | 14807 (13539-15796) | 0.97 (0.87-1.03) | 40930 (35366-44056) | 1.34 (1.19-1.42) | 1.24 (0.98-1.49) |
| Africa | 5341 (3307-6847) | 1.5 (0.97-1.96) | 8612 (5643-11389) | 1.1 (0.74-1.46) | -1.13 (-1.19--1.07) |
| African Region | 4480 (2653-5911) | 1.67 (1.02-2.26) | 7622 (4854-10337) | 1.23 (0.79-1.67) | -1.15 (-1.23--1.07) |
| America | 8409 (7744-8786) | 1.39 (1.27-1.46) | 16714 (15040-17723) | 1.26 (1.14-1.34) | -0.26 (-0.42--0.1) |
| Andean Latin America | 149 (109-189) | 0.53 (0.39-0.66) | 247 (197-300) | 0.4 (0.32-0.49) | -0.91 (-1.1--0.72) |
| Asia | 15945 (12145-18658) | 0.78 (0.6-0.91) | 29624 (25551-36803) | 0.66 (0.56-0.81) | -0.67 (-0.83--0.52) |
| Australasia | 112 (104-119) | 0.5 (0.46-0.53) | 623 (536-682) | 1.09 (0.96-1.18) | 2.93 (2.47-3.4) |
| Basic Health System | 10862 (8602-12373) | 0.69 (0.56-0.8) | 16188 (14031-20353) | 0.48 (0.42-0.61) | -1.33 (-1.45--1.21) |
| Caribbean | 218 (184-270) | 0.74 (0.64-0.91) | 500 (413-602) | 0.98 (0.8-1.18) | 1.11 (0.97-1.25) |
| Central Africa | 602 (325-838) | 1.85 (1.04-2.66) | 1274 (724-1855) | 1.67 (0.95-2.44) | -0.37 (-0.43--0.32) |
| Central Asia | 115 (102-131) | 0.2 (0.18-0.22) | 149 (130-168) | 0.18 (0.15-0.2) | -0.6 (-0.79--0.4) |
| Central Europe | 505 (481-543) | 0.36 (0.34-0.38) | 1274 (1163-1389) | 0.63 (0.58-0.69) | 1.91 (1.54-2.28) |
| Central Latin America | 426 (412-445) | 0.37 (0.36-0.39) | 1337 (1178-1505) | 0.54 (0.48-0.61) | 1.39 (0.82-1.97) |
| Central Sub-Saharan Africa | 464 (244-659) | 1.79 (1.01-2.58) | 1018 (558-1507) | 1.68 (0.94-2.54) | -0.24 (-0.3--0.18) |
| Commonwealth High Income | 823 (758-876) | 0.56 (0.51-0.6) | 3710 (3244-3938) | 1.27 (1.13-1.34) | 3.24 (2.77-3.7) |
| Commonwealth Low Income | 1587 (1053-1978) | 1.53 (1.03-1.92) | 2843 (1995-3778) | 1.17 (0.81-1.53) | -0.93 (-1.03--0.83) |
| Commonwealth Middle Income | 7156 (5114-8596) | 1.04 (0.75-1.24) | 13495 (10164-15856) | 0.85 (0.64-1) | -0.61 (-0.68--0.54) |
| East Asia | 3365 (1843-4424) | 0.4 (0.22-0.52) | 2813 (2368-3539) | 0.17 (0.14-0.21) | -3.43 (-3.77--3.08) |
| East Asia & Pacific - WB | 9210 (7195-10699) | 0.73 (0.59-0.85) | 16422 (13503-21115) | 0.58 (0.48-0.74) | -1.04 (-1.28--0.8) |
| Eastern Africa | 1630 (979-2138) | 1.77 (1.11-2.36) | 2580 (1716-3570) | 1.23 (0.8-1.69) | -1.34 (-1.39--1.28) |
| Eastern Europe | 729 (694-763) | 0.28 (0.27-0.3) | 2564 (2381-2740) | 0.91 (0.85-0.98) | 3.66 (3.17-4.14) |
| Eastern Mediterranean Region | 1972 (1548-2388) | 0.83 (0.64-1.12) | 3149 (2519-3950) | 0.64 (0.51-0.81) | -0.84 (-0.9--0.78) |
| Eastern Sub-Saharan Africa | 1868 (1077-2504) | 1.94 (1.15-2.64) | 3049 (1987-4273) | 1.38 (0.88-1.93) | -1.24 (-1.29--1.18) |
| Europe | 7136 (6468-7854) | 0.75 (0.68-0.82) | 22765 (19977-24472) | 1.36 (1.22-1.45) | 2.31 (1.98-2.64) |
| Europe & Central Asia - WB | 7225 (6552-7944) | 0.74 (0.67-0.81) | 22866 (20083-24572) | 1.33 (1.19-1.41) | 2.28 (1.94-2.61) |
| European Region | 7248 (6575-7970) | 0.74 (0.67-0.81) | 23003 (20204-24712) | 1.32 (1.18-1.4) | 2.28 (1.94-2.61) |
| High-income Asia Pacific | 1448 (1185-1646) | 0.82 (0.67-0.93) | 5584 (4268-6416) | 0.85 (0.68-0.97) | -0.48 (-1.06-0.1) |
| High-income North America | 5938 (5349-6339) | 1.67 (1.51-1.78) | 10589 (9281-11349) | 1.61 (1.44-1.72) | -0.09 (-0.2-0.01) |
| Latin America & Caribbean - WB | 2489 (2354-2619) | 0.84 (0.79-0.88) | 6174 (5746-6554) | 0.89 (0.83-0.95) | 0.32 (0.02-0.61) |
| Limited Health System | 9932 (7046-11762) | 1.11 (0.8-1.32) | 18195 (13820-21182) | 0.89 (0.67-1.03) | -0.75 (-0.81--0.69) |
| Middle East & North Africa - WB | 1192 (945-1478) | 0.68 (0.52-0.94) | 1716 (1407-2261) | 0.52 (0.43-0.69) | -0.72 (-0.78--0.66) |
| Minimal Health System | 1249 (720-1705) | 1.79 (1.07-2.52) | 2454 (1418-3517) | 1.63 (0.95-2.33) | -0.3 (-0.34--0.27) |
| North Africa and Middle East | 1989 (1566-2462) | 0.92 (0.7-1.25) | 2589 (2131-3420) | 0.58 (0.48-0.79) | -1.38 (-1.44--1.33) |
| North America | 5938 (5349-6339) | 1.67 (1.51-1.78) | 10590 (9281-11350) | 1.61 (1.44-1.72) | -0.09 (-0.2-0.01) |
| Northern Africa | 761 (576-984) | 0.84 (0.63-1.14) | 842 (659-1181) | 0.56 (0.44-0.79) | -1.2 (-1.27--1.13) |
| Oceania | 65 (45-102) | 1.9 (1.27-3.22) | 195 (142-271) | 2.14 (1.57-3.04) | 0.66 (0.55-0.76) |
| Region of the Americas | 8409 (7744-8786) | 1.39 (1.27-1.46) | 16714 (15040-17723) | 1.26 (1.14-1.34) | -0.26 (-0.42--0.1) |
| South-East Asia Region | 8615 (6970-11048) | 1.14 (0.89-1.46) | 15925 (13309-20677) | 0.93 (0.77-1.2) | -0.66 (-0.71--0.61) |
| South Asia | 5977 (4370-7297) | 0.96 (0.7-1.17) | 11823 (9270-14110) | 0.82 (0.64-0.97) | -0.42 (-0.5--0.35) |
| South Asia - WB | 6198 (4568-7555) | 0.96 (0.72-1.18) | 12206 (9575-14557) | 0.82 (0.64-0.97) | -0.42 (-0.49--0.34) |
| Southeast Asia | 4381 (3521-6260) | 1.56 (1.2-2.35) | 7494 (5825-10961) | 1.24 (0.95-1.82) | -0.81 (-0.87--0.75) |
| Southern Africa | 753 (484-952) | 1.33 (0.87-1.71) | 1346 (960-1700) | 1.18 (0.84-1.48) | -0.39 (-0.5--0.29) |
| Southern Latin America | 806 (732-865) | 1.84 (1.66-1.98) | 1421 (1299-1522) | 1.61 (1.48-1.73) | -0.18 (-0.4-0.03) |
| Southern Sub-Saharan Africa | 389 (277-479) | 1.09 (0.79-1.36) | 633 (531-812) | 0.97 (0.8-1.25) | -0.37 (-0.55--0.19) |
| Sub-Saharan Africa - WB | 4594 (2735-6092) | 1.7 (1.04-2.29) | 7789 (4929-10576) | 1.26 (0.81-1.73) | -1.12 (-1.2--1.04) |
| Tropical Latin America | 897 (865-933) | 0.8 (0.77-0.83) | 2686 (2512-2815) | 1.07 (1-1.12) | 0.94 (0.6-1.29) |
| Western Africa | 1595 (902-2301) | 1.78 (1.01-2.64) | 2570 (1431-3625) | 1.2 (0.68-1.69) | -1.57 (-1.72--1.42) |
| Western Europe | 5283 (4677-5945) | 0.92 (0.81-1.03) | 18320 (15574-19954) | 1.62 (1.41-1.75) | 2.38 (2.03-2.74) |
| Western Pacific Region | 6046 (4183-7224) | 0.58 (0.43-0.68) | 10366 (8518-12581) | 0.43 (0.35-0.52) | -1.46 (-1.79--1.13) |
| Western Sub-Saharan Africa | 1758 (994-2511) | 1.78 (1.01-2.61) | 2933 (1638-4135) | 1.23 (0.7-1.72) | -1.49 (-1.63--1.34) |
| World Bank High Income | 13518 (12256-14440) | 1.08 (0.98-1.16) | 37818 (32347-40858) | 1.41 (1.25-1.51) | 1.08 (0.83-1.32) |
| World Bank Low Income | 2641 (1592-3471) | 1.44 (0.91-1.91) | 4592 (2924-6259) | 1.14 (0.71-1.55) | -0.89 (-0.95--0.84) |
| World Bank Lower Middle Income | 11964 (9354-14555) | 1.01 (0.79-1.28) | 21320 (17436-26301) | 0.84 (0.7-1.05) | -0.6 (-0.65--0.55) |
| World Bank Upper Middle Income | 8727 (6544-10035) | 0.57 (0.43-0.66) | 14037 (12601-16167) | 0.45 (0.4-0.52) | -1.01 (-1.16--0.86) |
| Country |  |  |  |  |  |
| Afghanistan | 68 (45-102) | 0.91 (0.57-1.52) | 124 (92-167) | 0.85 (0.6-1.24) | -0.34 (-0.57--0.1) |
| Albania | 13 (8-21) | 0.55 (0.33-0.93) | 16 (11-24) | 0.4 (0.28-0.6) | -0.78 (-1.07--0.48) |
| Algeria | 94 (68-135) | 0.61 (0.42-0.99) | 164 (120-252) | 0.49 (0.36-0.75) | -0.44 (-0.55--0.32) |
| American Samoa | 0 (0-1) | 1.46 (0.88-2.77) | 1 (1-2) | 3 (1.43-4.39) | 3.83 (3.36-4.31) |
| Andorra | 0 (0-1) | 0.74 (0.41-1.43) | 1 (1-2) | 0.72 (0.47-1) | 0.21 (-0.05-0.48) |
| Angola | 83 (43-118) | 1.78 (1-2.62) | 190 (103-286) | 1.4 (0.8-2.12) | -1.02 (-1.11--0.93) |
| Antigua and Barbuda | 0 (0-0) | 0.79 (0.7-0.86) | 1 (1-1) | 1.17 (1.1-1.26) | 1.76 (1.29-2.24) |
| Argentina | 664 (593-719) | 2.19 (1.95-2.37) | 1078 (978-1161) | 1.9 (1.73-2.05) | -0.22 (-0.42--0.03) |
| Armenia | 3 (2-3) | 0.1 (0.08-0.11) | 4 (3-5) | 0.1 (0.08-0.12) | 0.27 (0.1-0.45) |
| Australia | 93 (86-99) | 0.49 (0.45-0.52) | 556 (479-612) | 1.15 (1-1.25) | 3.17 (2.54-3.8) |
| Austria | 44 (41-47) | 0.36 (0.33-0.39) | 170 (145-187) | 0.79 (0.69-0.87) | 3.33 (2.89-3.78) |
| Azerbaijan | 3 (2-4) | 0.05 (0.03-0.07) | 3 (2-4) | 0.03 (0.02-0.04) | -1.52 (-1.83--1.21) |
| Bahamas | 3 (3-3) | 1.47 (1.36-1.61) | 7 (6-9) | 1.68 (1.37-2.09) | 0.65 (0.48-0.81) |
| Bahrain | 2 (1-2) | 0.8 (0.62-1) | 5 (3-6) | 0.62 (0.41-0.8) | -0.75 (-0.99--0.5) |
| Bangladesh | 701 (522-902) | 1.34 (0.96-1.76) | 1347 (960-1798) | 1.03 (0.75-1.38) | -0.83 (-1.03--0.63) |
| Barbados | 4 (4-5) | 1.61 (1.5-1.74) | 8 (6-9) | 1.63 (1.32-1.99) | 0.2 (0.1-0.3) |
| Belarus | 73 (62-90) | 0.61 (0.51-0.76) | 94 (75-114) | 0.7 (0.56-0.85) | 0.27 (-0.02-0.55) |
| Belgium | 119 (106-129) | 0.78 (0.7-0.85) | 743 (608-826) | 2.59 (2.18-2.86) | 4.31 (3.28-5.36) |
| Belize | 0 (0-0) | 0.23 (0.17-0.28) | 1 (1-1) | 0.38 (0.34-0.43) | 1.79 (1.22-2.36) |
| Benin | 33 (20-48) | 1.41 (0.82-2.06) | 71 (40-108) | 1.1 (0.64-1.69) | -0.84 (-0.93--0.75) |
| Bermuda | 0 (0-0) | 0.56 (0.48-0.67) | 1 (1-1) | 0.79 (0.67-0.95) | 1.55 (1.11-2) |
| Bhutan | 3 (2-4) | 1.02 (0.66-1.47) | 4 (3-6) | 0.71 (0.51-0.95) | -1.32 (-1.47--1.18) |
| Bolivia (Plurinational State of) | 38 (24-54) | 0.79 (0.52-1.09) | 57 (37-81) | 0.58 (0.38-0.82) | -0.91 (-1--0.83) |
| Bosnia and Herzegovina | 8 (6-12) | 0.19 (0.14-0.28) | 7 (5-11) | 0.13 (0.08-0.19) | -1.16 (-1.33--0.99) |
| Botswana | 10 (5-17) | 1.38 (0.71-2.19) | 15 (8-21) | 0.79 (0.44-1.12) | -1.67 (-1.88--1.46) |
| Brazil | 868 (834-901) | 0.79 (0.76-0.83) | 2623 (2455-2753) | 1.07 (1-1.12) | 0.96 (0.61-1.32) |
| Brunei Darussalam | 2 (1-3) | 1.98 (1.45-2.6) | 4 (3-5) | 1.57 (1.21-1.96) | -0.27 (-0.44--0.11) |
| Bulgaria | 32 (28-36) | 0.3 (0.26-0.34) | 94 (75-118) | 0.78 (0.62-0.97) | 3.53 (3.12-3.94) |
| Burkina Faso | 98 (55-152) | 2.15 (1.22-3.36) | 194 (109-298) | 1.87 (1.06-2.94) | -0.29 (-0.35--0.24) |
| Burundi | 75 (42-111) | 2.42 (1.34-3.7) | 111 (66-167) | 1.71 (1.01-2.56) | -1.29 (-1.41--1.16) |
| Cabo Verde | 4 (2-6) | 1.51 (0.82-2.45) | 4 (2-7) | 0.95 (0.53-1.46) | -1.77 (-1.92--1.62) |
| Cambodia | 62 (41-103) | 1.17 (0.69-2.14) | 115 (65-214) | 0.92 (0.52-1.77) | -0.85 (-0.9--0.8) |
| Cameroon | 89 (46-131) | 1.8 (0.92-2.66) | 211 (106-334) | 1.37 (0.7-2.21) | -0.99 (-1.11--0.88) |
| Canada | 382 (336-430) | 1.21 (1.06-1.36) | 925 (810-1006) | 1.22 (1.08-1.32) | -0.05 (-0.24-0.13) |
| Central African Republic | 28 (14-43) | 2.18 (1.12-3.34) | 48 (24-78) | 1.92 (1.01-3.25) | -0.47 (-0.54--0.41) |
| Chad | 56 (33-88) | 1.79 (0.99-2.84) | 123 (70-182) | 1.65 (0.97-2.42) | -0.42 (-0.56--0.29) |
| Chile | 54 (52-57) | 0.55 (0.52-0.58) | 177 (162-190) | 0.7 (0.64-0.75) | 1.38 (0.77-2) |
| China | 3232 (1721-4286) | 0.4 (0.21-0.52) | 1778 (1387-2434) | 0.11 (0.08-0.15) | -5.35 (-5.84--4.85) |
| Colombia | 133 (127-139) | 0.58 (0.55-0.6) | 502 (415-597) | 0.93 (0.77-1.11) | 1.68 (1.02-2.35) |
| Comoros | 5 (2-7) | 1.91 (0.91-2.86) | 7 (4-10) | 1.29 (0.75-1.9) | -1.54 (-1.8--1.28) |
| Congo | 20 (9-29) | 1.71 (0.88-2.51) | 38 (21-56) | 1.31 (0.75-1.93) | -1.05 (-1.14--0.96) |
| Cook Islands | 0 (0-0) | 1.95 (1.34-3.27) | 0 (0-0) | 1.28 (0.91-1.92) | -1.29 (-1.4--1.18) |
| Costa Rica | 19 (17-20) | 0.94 (0.87-1) | 66 (58-73) | 1.21 (1.06-1.35) | 0.97 (0.71-1.23) |
| Croatia | 11 (10-12) | 0.19 (0.18-0.22) | 29 (24-33) | 0.34 (0.29-0.4) | 2.23 (1.84-2.63) |
| Cuba | 32 (29-34) | 0.31 (0.29-0.33) | 86 (74-97) | 0.48 (0.41-0.54) | 1.81 (1.51-2.1) |
| Cyprus | 19 (15-26) | 3.61 (2.64-4.85) | 28 (20-37) | 1.62 (1.08-2.14) | -2.53 (-2.88--2.18) |
| Czechia | 54 (48-60) | 0.42 (0.37-0.46) | 154 (130-178) | 0.78 (0.66-0.89) | 2.65 (2.31-2.98) |
| Côte d'Ivoire | 80 (40-119) | 1.59 (0.79-2.4) | 179 (90-275) | 1.29 (0.68-1.99) | -0.65 (-0.75--0.56) |
| Democratic People's Republic of Korea | 74 (50-115) | 0.46 (0.32-0.75) | 100 (55-198) | 0.36 (0.2-0.71) | -0.83 (-0.89--0.76) |
| Democratic Republic of the Congo | 321 (167-469) | 1.79 (0.99-2.66) | 726 (377-1121) | 1.81 (0.92-2.83) | 0.08 (0-0.15) |
| Denmark | 28 (26-30) | 0.34 (0.31-0.36) | 151 (128-166) | 1.14 (0.99-1.25) | 4.69 (4.05-5.32) |
| Djibouti | 3 (2-5) | 1.66 (0.94-2.52) | 9 (5-14) | 1.22 (0.71-1.91) | -1.09 (-1.17--1.01) |
| Dominica | 1 (1-1) | 1.43 (1.11-2.02) | 1 (1-1) | 1.24 (0.95-1.55) | -0.47 (-0.51--0.43) |
| Dominican Republic | 26 (19-40) | 0.5 (0.33-0.84) | 53 (36-69) | 0.5 (0.35-0.65) | 0.35 (0.16-0.55) |
| Ecuador | 19 (18-20) | 0.27 (0.25-0.29) | 76 (62-93) | 0.46 (0.38-0.56) | 1.87 (1.11-2.63) |
| Egypt | 467 (294-680) | 1.07 (0.78-1.41) | 361 (278-464) | 0.54 (0.42-0.76) | -2.15 (-2.29--2.01) |
| El Salvador | 7 (6-9) | 0.18 (0.14-0.22) | 8 (6-11) | 0.13 (0.09-0.17) | -0.81 (-0.99--0.63) |
| Equatorial Guinea | 4 (2-6) | 1.95 (1.04-3) | 6 (3-9) | 0.87 (0.44-1.44) | -3.19 (-3.57--2.82) |
| Eritrea | 34 (18-50) | 2.17 (1.19-3.29) | 55 (31-82) | 1.56 (0.87-2.43) | -1 (-1.16--0.83) |
| Estonia | 13 (11-15) | 0.73 (0.59-0.84) | 25 (22-28) | 1.12 (0.97-1.27) | 1.35 (1.1-1.59) |
| Eswatini | 5 (3-9) | 1.31 (0.68-2.09) | 8 (4-14) | 1.05 (0.45-1.68) | -0.45 (-0.65--0.25) |
| Ethiopia | 485 (260-705) | 1.89 (1.04-2.75) | 658 (410-997) | 1.15 (0.69-1.73) | -1.97 (-2.14--1.8) |
| Fiji | 9 (6-14) | 2 (1.38-3.31) | 16 (11-21) | 2.37 (1.58-3.12) | 1.15 (0.85-1.45) |
| Finland | 10 (9-12) | 0.15 (0.13-0.17) | 46 (40-51) | 0.33 (0.29-0.36) | 3.44 (2.79-4.09) |
| France | 1979 (1717-2273) | 2.27 (1.97-2.6) | 4012 (3407-4498) | 2.18 (1.88-2.43) | 0.18 (0.06-0.29) |
| Gabon | 8 (4-13) | 1.41 (0.71-2.21) | 11 (6-17) | 1.03 (0.56-1.62) | -1.12 (-1.21--1.04) |
| Gambia | 7 (4-11) | 1.65 (0.84-2.66) | 18 (10-28) | 1.48 (0.83-2.28) | -0.57 (-0.72--0.42) |
| Georgia | 10 (8-12) | 0.18 (0.15-0.22) | 15 (13-18) | 0.28 (0.24-0.34) | 2.04 (1.59-2.5) |
| Germany | 798 (705-895) | 0.63 (0.56-0.7) | 2829 (2377-3121) | 1.26 (1.08-1.37) | 2.75 (2.12-3.39) |
| Ghana | 134 (73-194) | 1.85 (0.99-2.73) | 208 (121-299) | 1.12 (0.64-1.63) | -1.91 (-2.05--1.76) |
| Greece | 176 (142-227) | 1.26 (1.03-1.61) | 343 (292-379) | 1.12 (0.99-1.23) | -0.48 (-0.54--0.42) |
| Greenland | 0 (0-0) | 0.86 (0.59-1.09) | 0 (0-1) | 0.86 (0.39-1.31) | 0.43 (0.09-0.78) |
| Grenada | 2 (1-2) | 2.12 (1.84-2.37) | 3 (2-3) | 2.57 (2.22-2.88) | 0.54 (0.4-0.68) |
| Guam | 1 (0-1) | 0.93 (0.54-1.87) | 1 (1-2) | 0.8 (0.42-1.07) | 1.48 (0.86-2.1) |
| Guatemala | 23 (20-27) | 0.44 (0.42-0.5) | 47 (40-54) | 0.38 (0.33-0.44) | -0.37 (-0.58--0.15) |
| Guinea | 64 (37-98) | 1.76 (1.02-2.76) | 106 (55-158) | 1.54 (0.82-2.36) | -0.42 (-0.49--0.36) |
| Guinea-Bissau | 10 (5-16) | 2.23 (1.15-3.31) | 17 (9-24) | 1.8 (1.04-2.61) | -0.66 (-0.7--0.62) |
| Guyana | 2 (2-2) | 0.43 (0.38-0.48) | 13 (10-17) | 1.95 (1.49-2.5) | 4.78 (3.57-5.99) |
| Haiti | 86 (61-127) | 1.99 (1.44-2.92) | 188 (123-260) | 2.05 (1.37-2.87) | 0.25 (0.19-0.32) |
| Honduras | 16 (13-24) | 0.62 (0.43-0.97) | 47 (35-62) | 0.72 (0.52-0.92) | 0.55 (0.35-0.75) |
| Hungary | 77 (70-84) | 0.57 (0.52-0.63) | 209 (178-244) | 1.15 (0.97-1.35) | 2.49 (2.01-2.97) |
| Iceland | 0 (0-1) | 0.16 (0.14-0.17) | 4 (3-4) | 0.6 (0.51-0.66) | 5.24 (4.39-6.11) |
| India | 4550 (3303-5661) | 0.9 (0.65-1.12) | 8990 (6987-10917) | 0.77 (0.59-0.93) | -0.36 (-0.47--0.25) |
| Indonesia | 1218 (810-2038) | 1.05 (0.68-1.86) | 2001 (1208-3637) | 0.87 (0.5-1.6) | -0.7 (-0.83--0.56) |
| Iran (Islamic Republic of) | 155 (124-199) | 0.47 (0.35-0.71) | 248 (200-322) | 0.33 (0.27-0.44) | -0.55 (-0.82--0.28) |
| Iraq | 57 (42-80) | 0.52 (0.36-0.81) | 100 (72-140) | 0.42 (0.31-0.59) | -1.05 (-1.23--0.88) |
| Ireland | 11 (10-12) | 0.28 (0.26-0.3) | 56 (47-64) | 0.67 (0.58-0.76) | 3.8 (2.96-4.64) |
| Israel | 18 (17-20) | 0.4 (0.37-0.43) | 117 (100-127) | 0.86 (0.76-0.94) | 3.22 (2.6-3.85) |
| Italy | 915 (759-1117) | 1.07 (0.88-1.3) | 2526 (2081-2804) | 1.41 (1.21-1.54) | 1.36 (1.06-1.66) |
| Jamaica | 24 (20-26) | 1.23 (1.04-1.36) | 46 (36-59) | 1.45 (1.13-1.88) | 0.76 (0.46-1.06) |
| Japan | 905 (817-954) | 0.6 (0.53-0.64) | 4790 (3616-5494) | 0.84 (0.68-0.94) | 0.38 (-0.39-1.16) |
| Jordan | 35 (27-44) | 1.71 (1.33-2.24) | 65 (49-88) | 0.85 (0.64-1.2) | -2.56 (-2.86--2.26) |
| Kazakhstan | 46 (41-55) | 0.32 (0.28-0.38) | 59 (47-72) | 0.33 (0.27-0.41) | -0.64 (-0.96--0.32) |
| Kenya | 117 (75-170) | 1.1 (0.71-1.63) | 251 (164-347) | 0.95 (0.6-1.34) | -0.31 (-0.38--0.24) |
| Kiribati | 1 (1-1) | 2.16 (1.44-3.76) | 2 (1-3) | 2.13 (1.43-3.72) | -0.05 (-0.09-0) |
| Kuwait | 3 (2-3) | 0.27 (0.24-0.3) | 8 (7-9) | 0.25 (0.21-0.29) | 0.82 (-0.46-2.12) |
| Kyrgyzstan | 4 (4-5) | 0.11 (0.1-0.12) | 7 (6-8) | 0.12 (0.1-0.15) | 0.64 (0.18-1.1) |
| Lao People's Democratic Republic | 37 (22-64) | 1.53 (0.85-2.78) | 65 (35-116) | 1.16 (0.63-2.09) | -0.83 (-0.89--0.77) |
| Latvia | 12 (10-13) | 0.38 (0.33-0.42) | 24 (21-27) | 0.83 (0.71-0.93) | 2.67 (2.43-2.91) |
| Lebanon | 30 (21-41) | 1.41 (1-1.92) | 35 (28-48) | 0.56 (0.44-0.76) | -3.42 (-3.61--3.23) |
| Lesotho | 12 (6-19) | 1.25 (0.63-2.04) | 17 (8-28) | 1.29 (0.58-2.11) | 0.66 (0.4-0.92) |
| Liberia | 23 (13-33) | 1.73 (0.93-2.51) | 42 (21-68) | 1.54 (0.79-2.48) | -0.68 (-0.85--0.5) |
| Libya | 19 (13-26) | 0.5 (0.34-0.76) | 28 (19-47) | 0.56 (0.38-0.9) | 0.75 (0.41-1.09) |
| Lithuania | 14 (11-16) | 0.34 (0.27-0.4) | 31 (27-35) | 0.72 (0.63-0.81) | 3 (2.66-3.34) |
| Luxembourg | 5 (4-5) | 0.93 (0.86-0.98) | 32 (28-36) | 2.69 (2.34-3) | 4.23 (3.28-5.18) |
| Madagascar | 204 (121-269) | 3.05 (1.83-4.13) | 412 (255-609) | 2.53 (1.52-3.79) | -0.64 (-0.69--0.6) |
| Malawi | 79 (44-111) | 1.52 (0.87-2.15) | 135 (78-206) | 1.38 (0.81-2.06) | -0.39 (-0.46--0.32) |
| Malaysia | 222 (172-265) | 1.97 (1.52-2.36) | 427 (320-515) | 1.53 (1.13-1.84) | -0.73 (-0.86--0.6) |
| Maldives | 1 (1-1) | 0.74 (0.55-0.97) | 2 (1-2) | 0.39 (0.3-0.51) | -2.06 (-2.16--1.96) |
| Mali | 75 (40-114) | 1.71 (0.9-2.65) | 141 (79-223) | 1.27 (0.7-2.05) | -1.03 (-1.09--0.97) |
| Malta | 2 (2-2) | 0.47 (0.43-0.51) | 16 (13-18) | 1.46 (1.26-1.66) | 4.22 (3.27-5.18) |
| Marshall Islands | 0 (0-1) | 2.45 (1.59-4.28) | 1 (1-1) | 2.74 (1.88-3.84) | 0.49 (0.36-0.61) |
| Mauritania | 18 (10-25) | 1.64 (0.91-2.35) | 28 (17-42) | 1.19 (0.71-1.79) | -1.19 (-1.24--1.13) |
| Mauritius | 2 (2-2) | 0.23 (0.22-0.25) | 14 (13-15) | 0.88 (0.81-0.93) | 4.54 (3.35-5.74) |
| Mexico | 166 (158-175) | 0.26 (0.25-0.27) | 483 (428-536) | 0.39 (0.35-0.43) | 1.64 (0.79-2.5) |
| Micronesia (Federated States of) | 1 (1-2) | 2.54 (1.63-4.25) | 2 (1-3) | 2.69 (1.85-3.81) | 0.38 (0.28-0.47) |
| Monaco | 2 (1-3) | 2.61 (1.82-3.46) | 2 (2-3) | 2.21 (1.51-3) | -0.49 (-0.7--0.27) |
| Mongolia | 5 (3-6) | 0.32 (0.22-0.45) | 6 (4-8) | 0.22 (0.16-0.29) | -1.56 (-1.68--1.43) |
| Montenegro | 1 (1-1) | 0.13 (0.1-0.16) | 1 (1-1) | 0.11 (0.08-0.14) | -0.52 (-0.67--0.37) |
| Morocco | 130 (91-187) | 0.69 (0.46-1.12) | 187 (134-294) | 0.58 (0.42-0.91) | -0.44 (-0.51--0.37) |
| Mozambique | 134 (74-195) | 1.99 (1.07-2.92) | 267 (148-423) | 1.94 (1.04-3.04) | 0.13 (0.05-0.22) |
| Myanmar | 426 (275-705) | 1.57 (0.97-2.78) | 513 (300-939) | 1.05 (0.61-1.94) | -1.66 (-1.82--1.5) |
| Namibia | 10 (5-15) | 1.22 (0.61-1.84) | 17 (9-27) | 1 (0.54-1.55) | -0.81 (-0.91--0.7) |
| Nauru | 0 (0-0) | 1.81 (1.17-3) | 0 (0-0) | 2.21 (1.5-3.28) | 0.83 (0.5-1.16) |
| Nepal | 105 (76-140) | 1.02 (0.75-1.36) | 208 (148-273) | 0.92 (0.67-1.21) | -0.22 (-0.31--0.12) |
| Netherlands | 153 (138-164) | 0.77 (0.69-0.82) | 1290 (1093-1425) | 3.29 (2.83-3.62) | 4.49 (3.3-5.68) |
| New Zealand | 19 (18-21) | 0.52 (0.47-0.55) | 67 (58-73) | 0.77 (0.68-0.84) | 1.72 (0.51-2.95) |
| Nicaragua | 15 (13-19) | 0.6 (0.49-0.81) | 24 (18-30) | 0.45 (0.34-0.57) | -0.6 (-0.75--0.46) |
| Niger | 64 (32-97) | 1.89 (0.95-2.97) | 156 (74-246) | 1.6 (0.81-2.54) | -0.65 (-0.75--0.56) |
| Nigeria | 870 (496-1294) | 1.78 (0.98-2.71) | 1177 (621-1728) | 1.02 (0.57-1.48) | -2.33 (-2.58--2.09) |
| Niue | 0 (0-0) | 1.94 (1.19-3.47) | 0 (0-0) | 2.11 (1.63-2.97) | -0.18 (-0.36-0.01) |
| North Macedonia | 4 (3-6) | 0.21 (0.15-0.31) | 6 (4-8) | 0.19 (0.12-0.26) | 0.3 (-0.05-0.66) |
| Northern Mariana Islands | 0 (0-0) | 0.7 (0.47-1.16) | 0 (0-1) | 1.07 (0.59-1.4) | 2.21 (1.78-2.64) |
| Norway | 34 (31-35) | 0.47 (0.43-0.5) | 149 (127-163) | 1.27 (1.1-1.38) | 3.44 (2.63-4.25) |
| Oman | 3 (2-4) | 0.3 (0.19-0.43) | 3 (2-4) | 0.14 (0.1-0.18) | -2.05 (-2.2--1.9) |
| Pakistan | 618 (466-790) | 0.98 (0.71-1.27) | 1274 (943-1687) | 0.92 (0.69-1.21) | -0.28 (-0.46--0.11) |
| Palau | 0 (0-0) | 1.46 (0.91-2.52) | 0 (0-0) | 1.55 (1.13-2.15) | 0.56 (0.36-0.76) |
| Palestine | 3 (2-4) | 0.23 (0.17-0.31) | 4 (3-5) | 0.15 (0.12-0.2) | -1.31 (-1.57--1.05) |
| Panama | 14 (13-16) | 0.84 (0.76-0.92) | 45 (36-54) | 1.02 (0.81-1.22) | 0.87 (0.75-0.98) |
| Papua New Guinea | 42 (27-67) | 1.9 (1.15-3.29) | 146 (100-214) | 2.18 (1.49-3.25) | 0.62 (0.56-0.69) |
| Paraguay | 30 (24-40) | 1.06 (0.85-1.43) | 62 (44-83) | 1.02 (0.71-1.37) | 0.26 (0.12-0.39) |
| Peru | 92 (65-117) | 0.57 (0.4-0.75) | 114 (83-151) | 0.33 (0.24-0.43) | -1.97 (-2.1--1.83) |
| Philippines | 374 (272-439) | 1.01 (0.78-1.35) | 810 (564-977) | 0.9 (0.67-1.09) | -0.14 (-0.24--0.05) |
| Poland | 167 (161-173) | 0.4 (0.38-0.41) | 529 (483-579) | 0.81 (0.75-0.89) | 1.98 (1.32-2.65) |
| Portugal | 59 (55-62) | 0.48 (0.45-0.51) | 497 (423-548) | 1.69 (1.47-1.84) | 4.85 (4.21-5.48) |
| Puerto Rico | 16 (15-17) | 0.45 (0.43-0.48) | 47 (39-55) | 0.8 (0.66-0.93) | 1.94 (1.34-2.54) |
| Qatar | 1 (1-1) | 0.58 (0.43-0.81) | 3 (2-5) | 0.28 (0.18-0.4) | -2.07 (-2.57--1.57) |
| Republic of Korea | 530 (291-711) | 2.57 (1.38-3.48) | 745 (547-1173) | 0.85 (0.63-1.33) | -4.16 (-4.44--3.88) |
| Republic of Moldova | 10 (9-11) | 0.23 (0.2-0.25) | 27 (24-31) | 0.54 (0.48-0.61) | 3.45 (3.15-3.76) |
| Romania | 57 (53-62) | 0.22 (0.2-0.24) | 113 (99-127) | 0.35 (0.3-0.39) | 1.79 (1.6-1.98) |
| Russian Federation | 388 (380-396) | 0.23 (0.23-0.24) | 2056 (1908-2212) | 1.06 (0.98-1.14) | 4.62 (3.92-5.31) |
| Rwanda | 100 (54-139) | 2.58 (1.43-3.75) | 105 (63-160) | 1.39 (0.81-2.15) | -2.5 (-2.68--2.31) |
| Saint Kitts and Nevis | 0 (0-0) | 0.47 (0.41-0.56) | 0 (0-0) | 0.53 (0.45-0.62) | 0.74 (0.48-1.01) |
| Saint Lucia | 1 (1-2) | 1.63 (1.51-1.77) | 5 (4-6) | 2.15 (1.78-2.54) | 0.83 (0.47-1.18) |
| Saint Vincent and the Grenadines | 0 (0-0) | 0.33 (0.3-0.35) | 1 (1-1) | 0.8 (0.71-0.91) | 2.41 (1.53-3.29) |
| Samoa | 2 (1-3) | 2.2 (1.44-3.97) | 3 (2-5) | 2.3 (1.71-3.29) | 0.34 (0.23-0.46) |
| San Marino | 1 (0-1) | 1.51 (1.14-2.1) | 1 (1-1) | 0.85 (0.53-1.21) | -0.98 (-1.33--0.63) |
| Sao Tome and Principe | 1 (0-1) | 1.18 (0.59-1.81) | 1 (1-2) | 1.07 (0.64-1.56) | -0.36 (-0.51--0.21) |
| Saudi Arabia | 43 (30-62) | 0.53 (0.33-0.88) | 99 (55-141) | 0.43 (0.29-0.55) | -0.28 (-0.55--0.01) |
| Senegal | 64 (35-97) | 1.68 (0.91-2.62) | 126 (75-187) | 1.43 (0.84-2.15) | -0.49 (-0.57--0.42) |
| Serbia | 47 (36-65) | 0.48 (0.36-0.65) | 51 (33-67) | 0.32 (0.21-0.42) | -0.99 (-1.11--0.87) |
| Seychelles | 1 (0-1) | 0.84 (0.55-1.43) | 1 (0-1) | 0.53 (0.32-0.94) | -1.07 (-1.17--0.97) |
| Sierra Leone | 40 (22-61) | 1.64 (0.9-2.61) | 68 (36-106) | 1.42 (0.79-2.22) | -0.48 (-0.58--0.37) |
| Singapore | 11 (10-11) | 0.54 (0.5-0.57) | 44 (38-48) | 0.54 (0.47-0.59) | -0.82 (-1.72-0.08) |
| Slovakia | 20 (15-28) | 0.34 (0.26-0.49) | 28 (18-36) | 0.32 (0.21-0.42) | 0.23 (0.02-0.45) |
| Slovenia | 7 (6-7) | 0.29 (0.27-0.31) | 18 (16-21) | 0.42 (0.36-0.48) | 1.26 (0.89-1.63) |
| Solomon Islands | 2 (1-3) | 1.54 (0.96-2.58) | 7 (5-10) | 1.84 (1.34-2.67) | 0.85 (0.74-0.96) |
| Somalia | 91 (43-146) | 2.58 (1.31-4.22) | 180 (81-296) | 1.99 (0.89-3.34) | -0.82 (-0.89--0.75) |
| South Africa | 325 (232-395) | 1.19 (0.86-1.46) | 508 (429-676) | 1.01 (0.86-1.33) | -0.61 (-0.8--0.42) |
| South Sudan | 55 (28-82) | 1.7 (0.93-2.59) | 69 (36-106) | 1.37 (0.75-2.17) | -0.93 (-1.24--0.63) |
| Spain | 381 (341-409) | 0.74 (0.66-0.8) | 2159 (1768-2417) | 1.75 (1.48-1.94) | 3.5 (2.56-4.45) |
| Sri Lanka | 152 (120-207) | 1.31 (1.03-1.81) | 257 (169-357) | 1.06 (0.71-1.47) | -0.15 (-0.4-0.11) |
| Sudan | 118 (81-172) | 0.81 (0.56-1.28) | 151 (104-219) | 0.58 (0.4-0.88) | -1.11 (-1.17--1.05) |
| Suriname | 1 (1-2) | 0.43 (0.34-0.64) | 2 (2-3) | 0.4 (0.28-0.53) | 0.18 (0.02-0.35) |
| Sweden | 135 (119-150) | 0.84 (0.74-0.94) | 225 (189-252) | 0.85 (0.73-0.95) | 0.58 (0.35-0.82) |
| Switzerland | 113 (100-126) | 1.05 (0.93-1.17) | 871 (686-986) | 3.68 (2.98-4.11) | 5.8 (4.88-6.72) |
| Syrian Arab Republic | 21 (14-29) | 0.27 (0.18-0.37) | 22 (15-29) | 0.19 (0.13-0.25) | -1.38 (-1.56--1.19) |
| Taiwan (Province of China) | 60 (57-63) | 0.43 (0.41-0.45) | 935 (798-1032) | 2.22 (1.93-2.44) | 7.9 (6.78-9.04) |
| Tajikistan | 1 (1-2) | 0.03 (0.02-0.04) | 2 (2-3) | 0.03 (0.02-0.04) | -0.56 (-0.88--0.24) |
| Thailand | 1381 (1094-1790) | 3.76 (2.95-4.91) | 2489 (1845-3367) | 2.54 (1.91-3.43) | -1.43 (-1.52--1.34) |
| Timor-Leste | 5 (3-9) | 1.33 (0.74-2.44) | 15 (8-28) | 1.51 (0.79-2.87) | 0.78 (0.62-0.95) |
| Togo | 27 (15-40) | 1.73 (0.94-2.63) | 64 (33-99) | 1.48 (0.76-2.29) | -0.56 (-0.63--0.49) |
| Tokelau | 0 (0-0) | 2.5 (1.56-4.39) | 0 (0-0) | 2.49 (1.88-3.41) | -0.46 (-0.65--0.27) |
| Tonga | 1 (1-1) | 1.46 (0.9-2.83) | 1 (1-2) | 1.47 (1.03-2.22) | 0.32 (0.22-0.42) |
| Trinidad and Tobago | 9 (9-10) | 1 (0.94-1.07) | 20 (15-25) | 1.13 (0.86-1.44) | 0.38 (0.21-0.54) |
| Tunisia | 34 (25-53) | 0.6 (0.43-1.03) | 74 (45-126) | 0.6 (0.37-1.03) | 0.31 (0.12-0.51) |
| Turkey | 634 (462-834) | 1.68 (1.24-2.19) | 736 (573-1023) | 0.85 (0.66-1.18) | -2.2 (-2.34--2.06) |
| Turkmenistan | 5 (4-7) | 0.17 (0.13-0.23) | 12 (8-17) | 0.26 (0.18-0.36) | 1.26 (1.02-1.5) |
| Tuvalu | 0 (0-0) | 2.97 (1.88-5.28) | 0 (0-0) | 2.57 (1.92-3.81) | -0.28 (-0.38--0.18) |
| Uganda | 154 (86-244) | 1.79 (1.03-2.9) | 243 (142-369) | 1.16 (0.66-1.76) | -1.71 (-1.83--1.58) |
| Ukraine | 219 (193-250) | 0.35 (0.31-0.41) | 307 (222-401) | 0.53 (0.38-0.69) | 1.09 (0.88-1.3) |
| United Arab Emirates | 7 (5-11) | 1.06 (0.71-1.53) | 29 (20-44) | 0.78 (0.56-1.01) | 0.78 (0.23-1.33) |
| United Kingdom | 277 (262-285) | 0.31 (0.29-0.32) | 2034 (1783-2162) | 1.41 (1.26-1.49) | 6.14 (5.13-7.17) |
| United Republic of Tanzania | 261 (154-360) | 1.98 (1.2-2.76) | 426 (276-637) | 1.35 (0.86-2.03) | -1.4 (-1.46--1.34) |
| United States of America | 5555 (5000-5925) | 1.71 (1.55-1.83) | 9664 (8447-10371) | 1.65 (1.48-1.77) | -0.08 (-0.2-0.04) |
| United States Virgin Islands | 1 (1-2) | 1.39 (1.06-2.05) | 1 (1-1) | 0.9 (0.64-1.17) | -0.88 (-1.14--0.61) |
| Uruguay | 87 (80-92) | 2.31 (2.13-2.45) | 166 (150-179) | 2.81 (2.56-3.03) | 0.78 (0.64-0.91) |
| Uzbekistan | 38 (29-46) | 0.21 (0.17-0.25) | 41 (33-51) | 0.14 (0.11-0.17) | -1.25 (-1.61--0.88) |
| Vanuatu | 1 (1-2) | 1.91 (1.16-3.45) | 4 (3-6) | 2.22 (1.63-3.37) | 0.49 (0.43-0.56) |
| Venezuela (Bolivarian Republic of) | 33 (30-36) | 0.27 (0.24-0.29) | 115 (86-148) | 0.4 (0.31-0.52) | 1.19 (0.83-1.54) |
| Viet Nam | 495 (290-925) | 1.15 (0.64-2.25) | 775 (426-1487) | 0.83 (0.45-1.61) | -1.13 (-1.17--1.1) |
| Yemen | 66 (46-92) | 0.82 (0.53-1.41) | 139 (97-199) | 0.81 (0.56-1.26) | -0.19 (-0.28--0.1) |
| Zambia | 68 (38-92) | 1.8 (1.05-2.46) | 120 (71-170) | 1.4 (0.84-2.01) | -0.97 (-1.08--0.87) |
| Zimbabwe | 27 (20-38) | 0.52 (0.4-0.71) | 68 (47-98) | 0.69 (0.49-0.95) | 1.48 (1.19-1.78) |

**Supplementary Table 4** Data related to DALYs

|  | Number of DALYs cases (95% UI) in 1990 | The age-standardized DALYs rate/100000 (95% UI) in 1990 | Number of DALYs cases (95% UI) in 2021 | The age-standardized DALYs rate/100000 (95% UI) in 2021 | EAPC (95% CI) |
| --- | --- | --- | --- | --- | --- |
| Global | 1333863 (1056184-1508950) | 28.32 (23.28-31.61) | 2076413 (1827084-2308504) | 25.56 (22.34-28.37) | -0.34 (-0.45--0.24) |
| Sex |  |  |  |  |  |
| Female | 580185 (410614-690233) | 23.88 (17.64-27.86) | 876094 (705775-1006976) | 20.65 (16.38-23.65) | -0.47 (-0.6--0.34) |
| Male | 753677 (574654-866034) | 32.84 (26.04-37.81) | 1200319 (1007093-1393512) | 30.57 (25.65-35.35) | -0.25 (-0.33--0.17) |
| Age |  |  |  |  |  |
| <5 years | 269701 (136344-364955) | 43.5 (21.99-58.87) | 128161 (84444-164724) | 19.47 (12.83-25.03) | -2.47 (-2.56--2.37) |
| 5-9 years | 31905 (18915-40966) | 5.47 (3.24-7.02) | 26162 (18174-32172) | 3.81 (2.65-4.68) | -1 (-1.12--0.88) |
| 10-14 years | 34040 (22181-42354) | 6.35 (4.14-7.91) | 38272 (27113-46275) | 5.74 (4.07-6.94) | -0.21 (-0.28--0.13) |
| 15-19 years | 54508 (38213-65396) | 10.49 (7.36-12.59) | 63348 (46554-77126) | 10.15 (7.46-12.36) | -0.24 (-0.35--0.14) |
| 20-24 years | 69456 (49944-79949) | 14.11 (10.15-16.25) | 87330 (66476-101310) | 14.62 (11.13-16.97) | -0.14 (-0.32-0.03) |
| 25-29 years | 80322 (59782-93163) | 18.15 (13.51-21.05) | 110328 (84685-128576) | 18.75 (14.39-21.85) | 0.05 (-0.13-0.23) |
| 30-34 years | 77992 (60739-93674) | 20.24 (15.76-24.3) | 122801 (100567-143777) | 20.32 (16.64-23.79) | 0.13 (0.03-0.23) |
| 35-39 years | 80137 (61219-93140) | 22.75 (17.38-26.44) | 130101 (103571-152468) | 23.2 (18.47-27.18) | 0.01 (-0.1-0.12) |
| 40-44 years | 76486 (59783-91716) | 26.7 (20.87-32.01) | 133919 (108336-163082) | 26.77 (21.66-32.6) | -0.26 (-0.43--0.09) |
| 45-49 years | 68247 (53681-81593) | 29.39 (23.12-35.14) | 126786 (104310-153395) | 26.78 (22.03-32.4) | -0.45 (-0.61--0.29) |
| 50-54 years | 68536 (56255-82649) | 32.24 (26.46-38.88) | 126926 (109874-152195) | 28.53 (24.7-34.21) | -0.48 (-0.61--0.35) |
| 55-59 years | 73551 (60723-88247) | 39.71 (32.79-47.65) | 142591 (122257-171759) | 36.03 (30.89-43.4) | -0.29 (-0.43--0.15) |
| 60-64 years | 72681 (59940-85068) | 45.25 (37.32-52.97) | 139313 (119982-161244) | 43.53 (37.49-50.38) | -0.3 (-0.44--0.16) |
| 65-69 years | 70851 (61011-82957) | 57.32 (49.36-67.11) | 143056 (125256-166071) | 51.86 (45.41-60.21) | -0.27 (-0.42--0.13) |
| 70-74 years | 61002 (51850-71200) | 72.05 (61.24-84.1) | 138354 (122828-157976) | 67.21 (59.67-76.75) | -0.24 (-0.37--0.12) |
| 75-79 years | 57273 (50697-65707) | 93.04 (82.36-106.74) | 117385 (104233-134167) | 89.01 (79.03-101.73) | -0.22 (-0.43--0.02) |
| 80-84 years | 43293 (37765-49423) | 122.38 (106.75-139.71) | 107120 (90654-122300) | 122.31 (103.51-139.64) | 0.11 (-0.14-0.37) |
| 85-89 years | 27459 (23549-31167) | 181.71 (155.84-206.26) | 92973 (75310-103495) | 203.34 (164.71-226.36) | 0.7 (0.42-0.98) |
| 90-94 years | 12281 (9827-13847) | 286.59 (229.33-323.13) | 67377 (51374-75665) | 376.63 (287.17-422.96) | 1.24 (0.96-1.52) |
| 95+ years | 4141 (3161-4752) | 406.75 (310.51-466.74) | 34110 (23994-39352) | 625.84 (440.24-722.01) | 1.59 (1.39-1.8) |
| SDI region |  |  |  |  |  |
| High-middle SDI | 199998 (162892-228994) | 20.26 (16.43-23.34) | 297630 (279573-319559) | 18.28 (17.13-19.83) | -0.41 (-0.54--0.27) |
| High SDI | 250615 (237800-260471) | 24.23 (23.03-25.18) | 536899 (489608-567759) | 28.62 (26.65-29.98) | 0.65 (0.46-0.85) |
| Low-middle SDI | 306138 (220303-369256) | 32.19 (24.76-38.46) | 443018 (360737-525612) | 26 (21.24-30.94) | -0.68 (-0.73--0.63) |
| Low SDI | 189372 (113107-240323) | 49.29 (30.93-62.96) | 339524 (220719-443733) | 40.71 (27.37-52.99) | -0.68 (-0.75--0.62) |
| Middle SDI | 386463 (298884-445527) | 25.57 (20.64-29.57) | 456988 (405398-570414) | 17.93 (15.87-22.4) | -1.29 (-1.38--1.2) |
| GBD region |  |  |  |  |  |
| Advanced Health System | 341698 (321610-362861) | 23.09 (21.67-24.67) | 750232 (688570-788092) | 30.81 (29.01-32.09) | 1.09 (0.87-1.31) |
| Africa | 255897 (148339-325261) | 49.54 (30.73-63.63) | 384791 (249066-510555) | 35.55 (23.53-46.99) | -1.19 (-1.25--1.13) |
| African Region | 203285 (116009-263702) | 53.05 (31.78-69.96) | 340552 (216070-458101) | 39.17 (25.29-52.94) | -1.14 (-1.22--1.06) |
| America | 229506 (218318-240482) | 35.36 (33.51-37.01) | 400744 (374203-420546) | 33.14 (31.13-34.92) | -0.16 (-0.3--0.01) |
| Andean Latin America | 7963 (5554-10167) | 21.64 (15.56-27.45) | 9992 (7992-12380) | 15.55 (12.46-19.21) | -1.05 (-1.25--0.85) |
| Asia | 673335 (496776-799275) | 24.65 (18.88-29.01) | 862935 (746821-1091115) | 18.32 (15.86-23.06) | -1.11 (-1.19--1.02) |
| Australasia | 2664 (2523-2808) | 11.82 (11.19-12.44) | 11315 (10254-12180) | 23.54 (21.72-25.26) | 2.52 (2.12-2.92) |
| Basic Health System | 506780 (383439-583833) | 24.4 (19.18-27.75) | 524269 (460859-654439) | 15.79 (13.86-19.74) | -1.6 (-1.71--1.5) |
| Caribbean | 9930 (8089-12704) | 29.65 (24.65-37.56) | 19027 (14888-23518) | 39.15 (30.22-48.95) | 1.13 (1.03-1.24) |
| Central Africa | 26717 (13378-37443) | 55.4 (30.51-77.57) | 54003 (30097-78511) | 48.91 (27.99-71.03) | -0.4 (-0.46--0.35) |
| Central Asia | 5956 (5102-7211) | 8.7 (7.62-10.21) | 6000 (5209-6883) | 6.39 (5.57-7.29) | -1.26 (-1.53--0.99) |
| Central Europe | 16837 (16029-18075) | 12.37 (11.77-13.26) | 33123 (30400-36042) | 19.28 (17.73-20.93) | 1.54 (1.21-1.87) |
| Central Latin America | 22715 (21740-23977) | 14.67 (14.16-15.33) | 46053 (40241-52614) | 18.43 (16.06-21.16) | 0.95 (0.42-1.48) |
| Central Sub-Saharan Africa | 20960 (9849-29779) | 53.24 (28.7-76.03) | 41708 (22801-61469) | 47.91 (26.5-70.81) | -0.35 (-0.41--0.29) |
| Commonwealth High Income | 18148 (17083-19328) | 13.2 (12.43-14.05) | 64889 (59730-67732) | 26.79 (25.13-27.86) | 2.79 (2.37-3.21) |
| Commonwealth Low Income | 69047 (44746-86211) | 46.43 (31.1-57.73) | 110918 (78026-148521) | 35.27 (25.04-47.02) | -0.92 (-0.98--0.85) |
| Commonwealth Middle Income | 298075 (205235-360743) | 32.59 (23.32-39.1) | 489510 (370702-572175) | 26.4 (19.92-31.04) | -0.7 (-0.75--0.64) |
| East Asia | 175399 (91665-237142) | 16.02 (8.43-21.6) | 70985 (59080-92254) | 4.59 (3.72-6.22) | -5.01 (-5.44--4.57) |
| East Asia & Pacific - WB | 391418 (290998-459927) | 23.69 (18.15-27.6) | 410442 (343887-544286) | 15.5 (13.01-20.59) | -1.75 (-1.9--1.59) |
| Eastern Africa | 80291 (45986-104418) | 59.12 (36.12-78.51) | 121749 (81646-168953) | 40.76 (27.28-56.1) | -1.37 (-1.43--1.31) |
| Eastern Europe | 29308 (27826-30957) | 11.96 (11.35-12.67) | 96915 (89999-103916) | 38.55 (35.78-41.49) | 3.68 (3.14-4.23) |
| Eastern Mediterranean Region | 103942 (70918-132169) | 29.19 (22.79-35.43) | 134572 (102337-162939) | 20.81 (16.44-25.62) | -1.03 (-1.06--0.99) |
| Eastern Sub-Saharan Africa | 90181 (50402-119385) | 63.38 (37.19-85.55) | 142679 (93701-199452) | 45.05 (29.51-62.88) | -1.26 (-1.32--1.2) |
| Europe | 173177 (160026-188176) | 19.42 (17.81-21.49) | 424181 (392340-446827) | 31.81 (30-33.31) | 1.88 (1.56-2.2) |
| Europe & Central Asia - WB | 178054 (164734-193568) | 19.14 (17.62-21.02) | 428873 (397188-451714) | 30.6 (28.86-32.03) | 1.78 (1.46-2.1) |
| European Region | 178609 (165258-194202) | 19.05 (17.54-20.92) | 431307 (399379-454278) | 30.42 (28.68-31.84) | 1.78 (1.46-2.1) |
| High-income Asia Pacific | 33788 (27709-38367) | 18.23 (14.91-20.78) | 73250 (59719-83213) | 15.34 (13.37-17.4) | -1.13 (-1.58--0.68) |
| High-income North America | 125855 (116765-135171) | 37.51 (34.82-40.37) | 211635 (195398-223605) | 38.51 (35.96-40.51) | 0.1 (0-0.19) |
| Latin America & Caribbean - WB | 104277 (98302-110564) | 27.77 (26.25-29.3) | 190400 (177875-203675) | 27.78 (25.81-29.88) | 0.1 (-0.19-0.39) |
| Limited Health System | 428249 (293038-513658) | 35.1 (25.02-41.57) | 690371 (523499-804907) | 27.86 (21.25-32.57) | -0.8 (-0.85--0.75) |
| Middle East & North Africa - WB | 69039 (46431-93381) | 26.44 (20.88-32.83) | 64217 (50980-80182) | 15.65 (12.64-19.75) | -1.58 (-1.62--1.54) |
| Minimal Health System | 55859 (29892-76276) | 55.07 (31.88-75.69) | 109188 (61796-156660) | 48.62 (28.31-69.77) | -0.37 (-0.4--0.34) |
| North Africa and Middle East | 106061 (72305-140664) | 32.59 (25.32-40.42) | 96363 (77215-121352) | 17.82 (14.5-22.67) | -1.89 (-1.92--1.86) |
| North America | 125854 (116764-135174) | 37.51 (34.82-40.36) | 211643 (195412-223607) | 38.51 (35.96-40.51) | 0.1 (0-0.19) |
| Northern Africa | 46604 (29099-66812) | 35.45 (26.08-46.48) | 33756 (25965-43296) | 17.94 (13.97-23.64) | -2.18 (-2.25--2.11) |
| Oceania | 3064 (2115-4549) | 61.83 (42.18-96.96) | 9093 (6504-12390) | 75.41 (54.52-104.32) | 0.91 (0.82-1.01) |
| Region of the Americas | 229506 (218318-240482) | 35.36 (33.51-37.01) | 400744 (374203-420546) | 33.14 (31.13-34.92) | -0.16 (-0.3--0.01) |
| South-East Asia Region | 355626 (279703-454535) | 35.2 (28.89-45) | 522447 (436011-678803) | 26.81 (22.31-34.71) | -0.89 (-0.95--0.84) |
| South Asia | 244851 (172253-306250) | 29.39 (21.68-36.13) | 401832 (315555-481638) | 24.21 (18.91-28.91) | -0.57 (-0.62--0.52) |
| South Asia - WB | 254008 (180488-317458) | 29.63 (22.04-36.43) | 416628 (327314-499917) | 24.43 (19.11-29.18) | -0.56 (-0.61--0.51) |
| Southeast Asia | 182933 (149420-256915) | 49.26 (39.74-69.7) | 254839 (202734-361941) | 36.97 (29.31-52.77) | -0.98 (-1.06--0.9) |
| Southern Africa | 35741 (22693-44169) | 47.63 (30.53-59.82) | 59153 (42229-74426) | 40.14 (28.83-50.54) | -0.53 (-0.66--0.4) |
| Southern Latin America | 21712 (19856-23235) | 46.57 (42.58-49.84) | 31625 (29434-33645) | 38.26 (35.73-40.69) | -0.44 (-0.62--0.26) |
| Southern Sub-Saharan Africa | 18702 (13083-22590) | 42.38 (30.12-51.77) | 27176 (23056-34834) | 35.81 (30.26-45.86) | -0.52 (-0.74--0.3) |
| Sub-Saharan Africa - WB | 209792 (119556-272487) | 54.25 (32.4-72.02) | 351694 (222125-474238) | 40.19 (25.75-54.35) | -1.12 (-1.2--1.04) |
| Tropical Latin America | 42306 (40695-44431) | 31.19 (30.07-32.63) | 84372 (80580-87806) | 33.94 (32.38-35.3) | 0.26 (-0.06-0.58) |
| Western Africa | 66544 (36582-92163) | 52.61 (29.78-76.45) | 116130 (62848-162984) | 36.49 (20.54-51.39) | -1.46 (-1.62--1.3) |
| Western Europe | 99575 (90138-111551) | 18.72 (17-20.96) | 275987 (246485-295826) | 30.6 (28.18-32.38) | 2.09 (1.71-2.47) |
| Western Pacific Region | 258831 (166110-323146) | 19.08 (12.53-23.6) | 225244 (196463-280526) | 10.32 (9-12.87) | -2.58 (-2.83--2.34) |
| Western Sub-Saharan Africa | 73105 (40161-100967) | 52.48 (29.71-75.41) | 132443 (72027-184106) | 37.21 (20.96-52.34) | -1.37 (-1.52--1.22) |
| World Bank High Income | 287223 (269469-305131) | 24 (22.51-25.53) | 637823 (580320-673851) | 30.11 (28.04-31.53) | 0.91 (0.71-1.11) |
| World Bank Low Income | 126242 (71013-165917) | 47.32 (28.77-62.46) | 209017 (130826-285958) | 37.25 (23.72-50.81) | -0.89 (-0.94--0.83) |
| World Bank Lower Middle Income | 519118 (388934-626260) | 33.26 (26.07-40.23) | 788833 (635672-948517) | 26.49 (21.54-32.07) | -0.74 (-0.78--0.69) |
| World Bank Upper Middle Income | 4e+05 (292182-473247) | 21.11 (15.51-24.88) | 438281 (402125-502876) | 14.93 (13.65-17.16) | -1.38 (-1.51--1.24) |
| Country |  |  |  |  |  |
| Afghanistan | 3063 (2123-4347) | 29.71 (20.47-43.22) | 6737 (4671-9174) | 27.02 (19.73-37.16) | -0.35 (-0.55--0.14) |
| Albania | 516 (350-773) | 18.08 (11.79-28.23) | 421 (294-622) | 12.99 (9.31-17.87) | -0.81 (-1.14--0.48) |
| Algeria | 5155 (3682-7469) | 21.84 (15.83-30.69) | 5754 (4345-8665) | 14.33 (10.79-21.69) | -1.25 (-1.3--1.2) |
| American Samoa | 14 (8-26) | 40.58 (23.85-77.77) | 42 (21-62) | 87.7 (43.41-129.04) | 4.12 (3.57-4.67) |
| Andorra | 9 (5-17) | 16.79 (9.81-30.3) | 21 (14-29) | 14.4 (9.32-20.11) | -0.15 (-0.38-0.09) |
| Angola | 3832 (1733-5555) | 53.22 (28.27-77.1) | 8215 (4381-12387) | 40.39 (22.2-60.32) | -1.08 (-1.17--0.99) |
| Antigua and Barbuda | 15 (13-16) | 26.73 (24.04-29.46) | 35 (33-38) | 34.47 (32.21-36.86) | 1.41 (0.91-1.92) |
| Argentina | 17859 (16077-19301) | 55.87 (50.32-60.36) | 23979 (22240-25576) | 44.73 (41.66-47.66) | -0.54 (-0.7--0.37) |
| Armenia | 126 (99-155) | 3.85 (3.06-4.68) | 110 (91-130) | 3.15 (2.63-3.67) | -0.37 (-0.59--0.16) |
| Australia | 2184 (2061-2315) | 11.6 (10.95-12.27) | 10053 (9064-10856) | 24.81 (22.85-26.69) | 2.79 (2.23-3.35) |
| Austria | 829 (777-876) | 7.25 (6.81-7.65) | 2629 (2329-2848) | 14.71 (13.38-15.79) | 3.1 (2.64-3.56) |
| Azerbaijan | 139 (92-201) | 1.96 (1.3-2.81) | 112 (72-170) | 1.09 (0.7-1.63) | -1.88 (-2.2--1.55) |
| Bahamas | 124 (113-135) | 54.69 (50.18-59.95) | 252 (201-321) | 58.99 (47.21-74.84) | 0.43 (0.28-0.58) |
| Bahrain | 79 (62-107) | 22.36 (18.24-28.63) | 173 (125-214) | 15.21 (10.74-18.95) | -1.09 (-1.28--0.9) |
| Bangladesh | 26975 (19422-35350) | 37.96 (28.33-48.77) | 42084 (30154-54476) | 28.13 (20.22-36.65) | -0.91 (-1.06--0.77) |
| Barbados | 140 (130-152) | 53.64 (49.56-58.18) | 201 (161-251) | 50.15 (39.88-63.47) | -0.06 (-0.18-0.06) |
| Belarus | 2812 (2285-3662) | 24.65 (19.79-32.63) | 3308 (2602-4006) | 27.95 (22.14-33.58) | 0.18 (-0.14-0.51) |
| Belgium | 2151 (1984-2290) | 15.34 (14.26-16.24) | 10994 (9410-12015) | 47.64 (42.19-51.52) | 4.14 (3.15-5.14) |
| Belize | 15 (12-18) | 9.26 (6.79-11.05) | 52 (46-58) | 13.23 (11.66-14.87) | 1.37 (0.86-1.89) |
| Benin | 1386 (781-2010) | 41.43 (24.41-59.82) | 3186 (1768-4815) | 33.45 (19.17-50.89) | -0.74 (-0.84--0.63) |
| Bermuda | 12 (10-14) | 17.87 (15.19-21.72) | 24 (20-29) | 23.89 (20.13-28.61) | 1.33 (0.91-1.76) |
| Bhutan | 126 (80-189) | 30.3 (20.42-43.52) | 141 (94-198) | 20.32 (13.87-28.43) | -1.45 (-1.64--1.25) |
| Bolivia (Plurinational State of) | 2193 (1315-3264) | 33.4 (21.12-47.86) | 2534 (1748-3479) | 22.64 (15.47-31.11) | -1.2 (-1.3--1.1) |
| Bosnia and Herzegovina | 274 (192-386) | 6.06 (4.22-8.47) | 187 (125-267) | 3.72 (2.6-5.18) | -1.33 (-1.5--1.16) |
| Botswana | 501 (265-831) | 50.75 (26.81-82.83) | 690 (400-1002) | 30.22 (17.24-42.65) | -1.54 (-1.76--1.32) |
| Brazil | 40916 (39340-42960) | 30.98 (29.83-32.36) | 82085 (78513-85469) | 33.88 (32.42-35.27) | 0.27 (-0.06-0.6) |
| Brunei Darussalam | 65 (47-89) | 43.24 (31.8-57.76) | 127 (101-173) | 34.11 (27.88-43.99) | -0.51 (-0.63--0.4) |
| Bulgaria | 1048 (918-1181) | 10.6 (9.31-11.86) | 2501 (1987-3135) | 25.39 (20.16-31.72) | 3.23 (2.83-3.63) |
| Burkina Faso | 3778 (2075-5814) | 59.81 (33.1-93.17) | 7767 (4168-11558) | 51.41 (29.22-79.37) | -0.35 (-0.43--0.27) |
| Burundi | 3695 (1940-5341) | 82.86 (45.33-123.21) | 5258 (3181-7763) | 55.19 (33.12-83.08) | -1.42 (-1.55--1.29) |
| Cabo Verde | 141 (82-222) | 48.55 (27.32-76.92) | 151 (90-232) | 28.94 (17.13-44.33) | -1.95 (-2.06--1.84) |
| Cambodia | 2708 (1870-4154) | 37.25 (24.63-61.97) | 4387 (2604-7812) | 28.7 (16.73-52.23) | -0.94 (-0.99--0.89) |
| Cameroon | 3654 (1814-5259) | 52.05 (26.78-76.77) | 9488 (4761-14394) | 41.95 (21.33-66.21) | -0.76 (-0.89--0.62) |
| Canada | 8064 (7136-9146) | 25.7 (22.7-29.16) | 16761 (15271-18022) | 27.05 (24.98-29.05) | 0.11 (-0.07-0.29) |
| Central African Republic | 1287 (574-1952) | 65.27 (32.7-100.15) | 2117 (1010-3458) | 56.94 (28.96-92.62) | -0.52 (-0.59--0.46) |
| Chad | 2206 (1217-3365) | 51.78 (29.49-81.6) | 5709 (3190-8407) | 49.09 (28.35-72.83) | -0.29 (-0.43--0.16) |
| Chile | 1679 (1610-1753) | 15 (14.38-15.67) | 4358 (4079-4636) | 18.27 (17.14-19.42) | 1.17 (0.61-1.73) |
| China | 169715 (85985-231840) | 16.05 (8.16-21.89) | 49925 (38779-69119) | 3.46 (2.65-5.01) | -6.26 (-6.8--5.71) |
| Colombia | 6480 (6125-6857) | 22.13 (21.1-23.28) | 15352 (12765-18273) | 29.75 (24.73-35.59) | 1.16 (0.54-1.79) |
| Comoros | 242 (108-363) | 65.8 (29.34-99.19) | 283 (170-399) | 43.07 (25.55-61.53) | -1.76 (-2.11--1.4) |
| Congo | 816 (373-1187) | 50.02 (24.15-74.04) | 1498 (800-2253) | 37.98 (20.84-56.09) | -1.09 (-1.21--0.98) |
| Cook Islands | 10 (7-17) | 62.79 (42.23-104.06) | 8 (6-12) | 43.81 (30.96-62.74) | -1.14 (-1.27--1.01) |
| Costa Rica | 722 (679-759) | 29.71 (27.92-31.36) | 1953 (1736-2173) | 37.3 (33.15-41.53) | 0.85 (0.62-1.09) |
| Croatia | 339 (304-377) | 6.07 (5.46-6.71) | 660 (558-767) | 9.56 (8.19-11.06) | 1.88 (1.48-2.29) |
| Cuba | 1174 (1088-1300) | 10.92 (10.13-12.02) | 2352 (2019-2661) | 15.29 (13.24-17.28) | 1.38 (1.09-1.67) |
| Cyprus | 388 (304-509) | 58.53 (45.65-76.07) | 497 (369-624) | 27.09 (20.09-33.43) | -2.41 (-2.65--2.17) |
| Czechia | 1645 (1474-1815) | 13.75 (12.45-15.07) | 3797 (3247-4361) | 23.4 (20.26-26.68) | 2.33 (2.01-2.66) |
| Côte d'Ivoire | 3773 (1864-5568) | 46.95 (23.62-70.55) | 8227 (4064-12474) | 39.94 (20.29-61.11) | -0.47 (-0.58--0.35) |
| Democratic People's Republic of Korea | 3578 (2436-5366) | 17.43 (12.04-26.43) | 3289 (1813-6274) | 12.3 (6.8-23.73) | -1.16 (-1.24--1.08) |
| Democratic Republic of the Congo | 14561 (6829-21614) | 52.95 (28.23-77.77) | 29241 (15285-45424) | 51.36 (26.83-79.07) | -0.04 (-0.11-0.04) |
| Denmark | 565 (530-596) | 7.69 (7.22-8.15) | 2437 (2157-2650) | 21.57 (19.42-23.28) | 4.08 (3.41-4.77) |
| Djibouti | 165 (92-241) | 54.44 (30.8-80.17) | 400 (240-628) | 38.76 (23.37-59.38) | -1.22 (-1.34--1.09) |
| Dominica | 28 (22-38) | 43.9 (34.67-60.68) | 29 (22-36) | 38.82 (29.19-48.97) | -0.37 (-0.5--0.24) |
| Dominican Republic | 1411 (1079-2024) | 20.59 (15.03-31.03) | 2168 (1471-2842) | 19.78 (13.51-25.77) | 0.3 (0.15-0.45) |
| Ecuador | 988 (925-1062) | 10.68 (10.07-11.36) | 2898 (2384-3469) | 16.65 (13.75-19.96) | 1.48 (0.7-2.27) |
| Egypt | 30898 (15738-49247) | 47.68 (30.06-69.33) | 17297 (12292-22068) | 18.44 (13.99-23.3) | -3.17 (-3.31--3.03) |
| El Salvador | 366 (266-466) | 7.13 (5.45-8.89) | 303 (208-396) | 4.82 (3.3-6.32) | -1.07 (-1.25--0.88) |
| Equatorial Guinea | 176 (81-264) | 58.04 (29.61-89.62) | 250 (113-428) | 25.66 (12.15-42.35) | -3.29 (-3.67--2.91) |
| Eritrea | 1767 (917-2606) | 72.39 (38.74-107.96) | 2573 (1495-3885) | 50.57 (28.89-75.82) | -1.09 (-1.26--0.92) |
| Estonia | 528 (423-613) | 30.9 (24.98-35.84) | 694 (603-789) | 38.49 (33.61-43.85) | 0.59 (0.31-0.87) |
| Eswatini | 271 (144-440) | 47.29 (24.87-77.33) | 415 (186-682) | 40.9 (17.84-67.49) | -0.23 (-0.45-0) |
| Ethiopia | 23584 (12072-34968) | 62.99 (33.55-91.7) | 29649 (19118-44325) | 36.85 (23.21-55.95) | -2.12 (-2.27--1.97) |
| Fiji | 381 (259-626) | 63.84 (43.99-104.5) | 631 (426-845) | 73.96 (49.83-97.26) | 1.15 (0.82-1.47) |
| Finland | 230 (205-260) | 3.46 (3.09-3.92) | 793 (706-862) | 7.38 (6.77-7.97) | 3.29 (2.61-3.97) |
| France | 34184 (30258-39116) | 42.59 (37.87-48.84) | 58693 (51496-64730) | 41.45 (37.19-45.18) | 0.22 (0.12-0.32) |
| Gabon | 288 (136-431) | 40.01 (19.57-61.03) | 387 (203-631) | 28.59 (15.26-45.47) | -1.18 (-1.27--1.09) |
| Gambia | 305 (153-469) | 48.43 (25.23-77.13) | 777 (455-1194) | 45.55 (25.83-70.23) | -0.46 (-0.66--0.26) |
| Georgia | 420 (341-502) | 7.7 (6.26-9.1) | 428 (357-507) | 9.57 (8.05-11.29) | 1.09 (0.52-1.67) |
| Germany | 15579 (14120-17349) | 13.44 (12.27-14.91) | 44210 (39067-47982) | 24.5 (22.3-26.31) | 2.47 (1.82-3.13) |
| Ghana | 5732 (3155-8080) | 56.76 (31.02-82.25) | 8499 (5132-12215) | 33.58 (19.96-47.75) | -1.98 (-2.14--1.82) |
| Greece | 3104 (2563-3981) | 22.52 (18.82-28.5) | 4959 (4403-5398) | 21.1 (19.22-22.67) | -0.28 (-0.35--0.21) |
| Greenland | 9 (7-12) | 21.72 (15.29-27.39) | 13 (6-19) | 19.24 (9.66-28.24) | -0.01 (-0.34-0.32) |
| Grenada | 55 (49-62) | 73.84 (65.75-82.7) | 86 (73-98) | 77 (66.12-87.84) | 0.11 (-0.02-0.23) |
| Guam | 26 (15-51) | 24.05 (13.96-48.23) | 52 (28-70) | 30.15 (15.87-40.65) | 2.63 (2.07-3.2) |
| Guatemala | 1323 (1131-1618) | 16.52 (14.92-19.45) | 2119 (1779-2480) | 15.07 (12.65-17.62) | -0.13 (-0.35-0.09) |
| Guinea | 2557 (1353-3706) | 52.83 (30.22-80.9) | 4609 (2361-6941) | 47.15 (24.47-71.1) | -0.31 (-0.38--0.24) |
| Guinea-Bissau | 466 (225-696) | 68.15 (34.64-102.96) | 778 (439-1126) | 55.48 (31.53-80.27) | -0.62 (-0.68--0.55) |
| Guyana | 107 (94-121) | 16.04 (14.23-17.87) | 527 (393-688) | 70.69 (52.92-91.99) | 4.69 (3.49-5.91) |
| Haiti | 4474 (2927-6711) | 76.03 (53.45-111.92) | 9015 (5843-12772) | 77.27 (50.84-107.63) | 0.25 (0.19-0.31) |
| Honduras | 775 (609-1110) | 20.84 (16.01-30.57) | 1688 (1227-2325) | 21.16 (15.69-28.28) | 0.08 (-0.08-0.24) |
| Hungary | 2421 (2180-2696) | 19.49 (17.63-21.61) | 5020 (4229-5931) | 32.9 (27.57-38.69) | 1.8 (1.39-2.21) |
| Iceland | 10 (9-11) | 3.55 (3.32-3.77) | 66 (58-73) | 12.4 (11.03-13.65) | 4.91 (4.11-5.7) |
| India | 187494 (130197-236627) | 28.28 (20.59-35.18) | 297736 (231113-360859) | 22.81 (17.73-27.55) | -0.65 (-0.7--0.59) |
| Indonesia | 53280 (35813-86059) | 35.61 (23.77-59.25) | 77190 (48486-135622) | 28.11 (17.41-49.97) | -0.82 (-0.97--0.67) |
| Iran (Islamic Republic of) | 8359 (6221-10911) | 16.7 (13.44-21.17) | 7667 (6206-9654) | 9.34 (7.59-11.71) | -1.23 (-1.51--0.96) |
| Iraq | 2839 (2089-3969) | 17.31 (12.73-24.69) | 3851 (2839-5356) | 12.05 (8.85-16.64) | -1.48 (-1.68--1.28) |
| Ireland | 224 (213-235) | 5.68 (5.41-5.96) | 956 (843-1067) | 12.8 (11.36-14.27) | 3.53 (2.63-4.43) |
| Israel | 392 (368-417) | 8.19 (7.67-8.72) | 2036 (1847-2199) | 16.75 (15.25-18.1) | 3.11 (2.43-3.79) |
| Italy | 18763 (15806-23047) | 24.22 (20.39-29.82) | 37529 (32666-40775) | 28.25 (25.71-30.21) | 0.84 (0.59-1.09) |
| Jamaica | 915 (778-1012) | 43.45 (36.59-48.35) | 1420 (1085-1875) | 46.83 (35.75-61.8) | 0.4 (0.1-0.71) |
| Japan | 19318 (18122-20030) | 12.66 (11.83-13.13) | 59336 (47754-66049) | 15.13 (13.45-16.23) | -0.12 (-0.79-0.55) |
| Jordan | 2036 (1505-2588) | 60.55 (46.66-76.36) | 2747 (2159-3568) | 27.7 (21.68-36.72) | -2.82 (-3.09--2.55) |
| Kazakhstan | 2144 (1860-2652) | 13.59 (11.92-16.61) | 2190 (1703-2673) | 11.35 (8.88-13.79) | -1.4 (-1.79--1.01) |
| Kenya | 5644 (3569-7877) | 34.87 (22.79-50.65) | 11174 (7550-15209) | 29.52 (19.72-40.33) | -0.37 (-0.45--0.29) |
| Kiribati | 42 (31-63) | 72.29 (50.7-115.31) | 74 (51-115) | 71.12 (49.76-114.61) | -0.04 (-0.09-0.01) |
| Kuwait | 147 (132-162) | 10.37 (9.36-11.51) | 309 (265-363) | 8.49 (7.27-9.91) | 0.47 (-0.77-1.73) |
| Kyrgyzstan | 236 (212-262) | 5.25 (4.74-5.78) | 306 (252-368) | 4.76 (3.91-5.71) | -0.18 (-0.6-0.25) |
| Lao People's Democratic Republic | 1651 (1016-2718) | 51.48 (30.66-88.59) | 2971 (1623-5130) | 42.94 (23.39-74.64) | -0.38 (-0.47--0.29) |
| Latvia | 472 (416-526) | 16.11 (14.28-17.89) | 767 (656-870) | 31.73 (27.1-36.11) | 2.17 (1.92-2.42) |
| Lebanon | 1056 (718-1427) | 40.8 (28.92-55.45) | 915 (735-1167) | 15.69 (12.62-19.98) | -3.47 (-3.64--3.29) |
| Lesotho | 488 (251-817) | 42.33 (21.6-69.69) | 787 (367-1292) | 48.14 (22.02-79.56) | 0.97 (0.7-1.24) |
| Liberia | 1011 (523-1429) | 52.23 (28.74-74.18) | 1909 (964-3087) | 47.73 (24.51-77.19) | -0.5 (-0.66--0.34) |
| Libya | 1349 (915-2034) | 26.29 (18.55-36.86) | 1269 (874-1831) | 23.43 (16.4-33.45) | 0.03 (-0.31-0.38) |
| Lithuania | 573 (467-677) | 14.51 (11.87-17.18) | 983 (855-1102) | 27.43 (24.06-30.46) | 2.55 (2.18-2.93) |
| Luxembourg | 94 (88-99) | 18.72 (17.64-19.72) | 501 (443-551) | 47.23 (42.45-51.98) | 3.68 (2.75-4.6) |
| Madagascar | 9767 (5544-13136) | 105.5 (62.56-140.76) | 20763 (13033-29870) | 91.07 (56.51-135.06) | -0.54 (-0.6--0.49) |
| Malawi | 4082 (2091-5823) | 51 (28.61-71.66) | 6371 (3757-9492) | 45.13 (26.37-68.3) | -0.47 (-0.54--0.39) |
| Malaysia | 9175 (7163-11043) | 64.51 (50.13-76.56) | 15002 (11374-18361) | 47.15 (35.68-57.01) | -1 (-1.09--0.91) |
| Maldives | 34 (24-47) | 22.12 (16.35-30.36) | 66 (43-109) | 12.24 (8.86-17.95) | -1.89 (-1.99--1.8) |
| Mali | 3224 (1628-4710) | 51.6 (27.28-79.13) | 6539 (3582-10044) | 39.39 (22.26-62.43) | -0.94 (-1--0.88) |
| Malta | 39 (36-41) | 9.61 (8.96-10.23) | 259 (228-291) | 30.26 (26.98-33.75) | 4.32 (3.39-5.24) |
| Marshall Islands | 20 (13-32) | 73.15 (47.78-120.07) | 44 (28-64) | 88.55 (58.87-126.01) | 0.77 (0.62-0.91) |
| Mauritania | 664 (387-920) | 46.89 (27.02-66.04) | 1059 (668-1540) | 34.23 (21.5-50.69) | -1.18 (-1.25--1.11) |
| Mauritius | 67 (63-71) | 7.13 (6.73-7.58) | 434 (399-460) | 28.63 (26.22-30.43) | 5.16 (3.88-6.45) |
| Mexico | 9957 (9324-10794) | 11.37 (10.81-12.11) | 18226 (16277-20217) | 14.43 (12.89-16.05) | 1.14 (0.39-1.89) |
| Micronesia (Federated States of) | 55 (35-86) | 77.02 (48.47-124.67) | 74 (49-108) | 82.33 (54.57-117.72) | 0.41 (0.32-0.49) |
| Monaco | 34 (24-45) | 56.88 (41.11-73.29) | 39 (27-53) | 46.32 (31.74-61.87) | -0.69 (-0.91--0.47) |
| Mongolia | 222 (144-327) | 12.21 (8.3-17.24) | 248 (185-329) | 7.91 (5.91-10.45) | -1.59 (-1.71--1.48) |
| Montenegro | 28 (22-36) | 4.43 (3.42-5.59) | 27 (19-35) | 3.23 (2.35-4.25) | -0.95 (-1.17--0.73) |
| Morocco | 6907 (4970-9729) | 26.91 (19.39-37.96) | 6231 (4519-9606) | 17.65 (12.95-26.96) | -1.21 (-1.32--1.11) |
| Mozambique | 5818 (3131-8378) | 60.31 (33.31-87.92) | 11964 (6753-18479) | 59.64 (33.15-94.84) | 0.18 (0.09-0.27) |
| Myanmar | 19251 (12875-29312) | 55.91 (36.8-89.65) | 20383 (12458-35591) | 37.21 (22.71-64.89) | -1.68 (-1.85--1.5) |
| Namibia | 466 (234-735) | 44.08 (22.28-68.36) | 767 (433-1242) | 36.18 (20.25-57.53) | -0.78 (-0.88--0.69) |
| Nauru | 5 (3-8) | 59.62 (37.86-102.78) | 7 (4-11) | 74.79 (47.86-111.3) | 0.93 (0.59-1.27) |
| Nepal | 4492 (3036-6026) | 30.29 (22.03-40.32) | 7099 (5034-9373) | 26.5 (18.87-34.74) | -0.28 (-0.34--0.23) |
| Netherlands | 2959 (2764-3127) | 15.78 (14.81-16.61) | 18839 (16580-20497) | 55.85 (50.06-60.1) | 4.03 (2.9-5.17) |
| New Zealand | 480 (451-509) | 12.89 (12.12-13.67) | 1262 (1154-1366) | 16.91 (15.67-18.24) | 1.23 (0.02-2.46) |
| Nicaragua | 908 (671-1179) | 24.06 (19.73-30.55) | 982 (783-1246) | 16.24 (12.93-20.49) | -0.95 (-1.08--0.82) |
| Niger | 2997 (1363-4605) | 54.93 (28.03-84.48) | 7016 (3231-11092) | 45.25 (21.93-72.13) | -0.76 (-0.88--0.65) |
| Nigeria | 35407 (19463-51468) | 52.46 (29.53-78.18) | 55625 (27750-81306) | 31.59 (16.78-46.56) | -2.15 (-2.4--1.9) |
| Niue | 1 (1-2) | 57.56 (35.16-102.15) | 1 (1-2) | 83.01 (65.27-113.14) | 0.26 (-0.1-0.61) |
| North Macedonia | 133 (101-200) | 6.92 (5.3-10.28) | 151 (101-200) | 5.25 (3.54-6.86) | -0.18 (-0.51-0.14) |
| Northern Mariana Islands | 7 (4-12) | 19.4 (12.91-31.42) | 15 (8-19) | 28.82 (16.42-38.13) | 2.18 (1.8-2.56) |
| Norway | 626 (591-655) | 9.95 (9.46-10.38) | 2236 (2001-2410) | 21.91 (19.96-23.38) | 2.91 (2.14-3.69) |
| Oman | 148 (96-221) | 9.34 (6.23-13.4) | 147 (109-199) | 4.18 (3.13-5.44) | -2.03 (-2.24--1.81) |
| Pakistan | 25764 (18936-32994) | 29.59 (22.65-38.06) | 54772 (38395-71305) | 28.96 (21.1-38.03) | -0.07 (-0.22-0.08) |
| Palau | 6 (4-10) | 44.52 (27.56-74.68) | 9 (7-12) | 49.33 (36.2-66.26) | 0.7 (0.53-0.88) |
| Palestine | 153 (109-206) | 7.48 (5.62-9.85) | 179 (142-251) | 4.47 (3.57-6.04) | -1.53 (-1.73--1.32) |
| Panama | 586 (539-649) | 28.33 (25.99-31.26) | 1434 (1159-1714) | 33.31 (26.94-39.84) | 0.75 (0.61-0.9) |
| Papua New Guinea | 2041 (1300-3105) | 64.51 (41.57-103.63) | 7087 (4797-10121) | 78.45 (53.82-114.25) | 0.81 (0.74-0.88) |
| Paraguay | 1390 (1122-1820) | 38.95 (31.45-52.09) | 2287 (1674-3009) | 34.37 (25.05-45.66) | -0.07 (-0.21-0.07) |
| Peru | 4782 (3191-6182) | 23.08 (16.09-29.54) | 4561 (3428-6091) | 12.81 (9.66-17.16) | -1.95 (-2.07--1.83) |
| Philippines | 17487 (11807-21041) | 34.38 (24.65-40.26) | 34095 (22994-41928) | 32.11 (22.07-38.98) | 0.04 (-0.07-0.16) |
| Poland | 5888 (5684-6112) | 14.52 (14.04-15.06) | 14420 (13264-15755) | 25.57 (23.53-27.78) | 1.63 (1.05-2.21) |
| Portugal | 1324 (1263-1385) | 11.06 (10.56-11.55) | 7605 (6768-8218) | 32.22 (29.51-34.39) | 4.13 (3.5-4.75) |
| Puerto Rico | 566 (531-600) | 15.8 (14.82-16.71) | 1242 (1030-1455) | 28.14 (23.57-32.99) | 1.89 (1.27-2.51) |
| Qatar | 43 (30-68) | 15.2 (11.15-22.79) | 162 (112-224) | 7.81 (5.31-10.74) | -1.77 (-2.11--1.43) |
| Republic of Korea | 14089 (8197-18483) | 48.47 (27.66-63.83) | 12938 (9902-19743) | 16.08 (12.5-24.1) | -4.14 (-4.41--3.87) |
| Republic of Moldova | 473 (427-521) | 10.47 (9.53-11.48) | 997 (888-1121) | 22.39 (19.75-25.31) | 3.05 (2.78-3.33) |
| Romania | 1952 (1782-2145) | 7.8 (7.08-8.57) | 3072 (2684-3488) | 11.28 (9.87-12.75) | 1.53 (1.37-1.7) |
| Russian Federation | 15729 (15406-16049) | 9.76 (9.57-9.97) | 78150 (72219-84467) | 44.63 (41.36-48.3) | 4.61 (3.85-5.38) |
| Rwanda | 5077 (2547-7524) | 90.02 (48.43-129.08) | 4647 (2915-7077) | 44.72 (27.39-67.86) | -2.79 (-2.99--2.6) |
| Saint Kitts and Nevis | 6 (5-7) | 15.73 (13.49-19.01) | 10 (8-12) | 14.69 (12.2-17.63) | -0.07 (-0.26-0.13) |
| Saint Lucia | 53 (49-57) | 50.02 (46.23-54.72) | 145 (119-173) | 66.4 (54.83-79.74) | 0.99 (0.61-1.36) |
| Saint Vincent and the Grenadines | 11 (10-12) | 12.18 (11.27-13.16) | 34 (30-40) | 27.25 (23.7-31.46) | 2.08 (1.22-2.95) |
| Samoa | 75 (49-127) | 64.35 (42.11-112.11) | 125 (91-176) | 71.46 (51.96-100.45) | 0.57 (0.46-0.68) |
| San Marino | 9 (7-13) | 27.86 (21.99-37.01) | 12 (8-17) | 16.54 (10.47-23.71) | -0.78 (-1.09--0.48) |
| Sao Tome and Principe | 34 (18-49) | 37.02 (19.53-55.68) | 56 (34-83) | 33.14 (20.27-48.2) | -0.46 (-0.64--0.28) |
| Saudi Arabia | 2158 (1526-3000) | 17.25 (11.85-24.74) | 4131 (2207-6029) | 12.49 (7.83-16.76) | -0.63 (-0.89--0.36) |
| Senegal | 2793 (1525-4143) | 51.14 (27.01-78.16) | 5166 (3090-7634) | 43.76 (26.23-64.47) | -0.41 (-0.52--0.31) |
| Serbia | 1463 (1123-1987) | 14.17 (10.98-19.19) | 1222 (812-1573) | 8.9 (6.19-11.28) | -1.16 (-1.31--1.01) |
| Seychelles | 19 (13-31) | 28.43 (19.33-46.59) | 20 (13-33) | 17.56 (11.04-28.8) | -1.09 (-1.2--0.99) |
| Sierra Leone | 1769 (871-2672) | 50.46 (26.65-78.17) | 3163 (1609-4918) | 45.53 (23.93-70.45) | -0.3 (-0.38--0.21) |
| Singapore | 315 (301-330) | 12.76 (12.14-13.32) | 848 (774-911) | 10.97 (10.03-11.76) | -1.26 (-2.09--0.41) |
| Slovakia | 657 (507-925) | 11.78 (9.07-16.46) | 774 (518-1030) | 10.32 (6.93-13.69) | 0.1 (-0.13-0.33) |
| Slovenia | 203 (188-219) | 8.78 (8.11-9.45) | 389 (337-448) | 10.6 (9.11-12.25) | 0.65 (0.27-1.04) |
| Solomon Islands | 89 (57-140) | 43.27 (27.21-70.43) | 283 (199-409) | 54.92 (38.96-79.81) | 1.05 (0.95-1.15) |
| Somalia | 4671 (2143-7423) | 84.04 (41.39-136.84) | 9136 (4259-15229) | 63.83 (29.19-105.32) | -0.89 (-0.97--0.81) |
| South Africa | 15742 (10777-18840) | 47.79 (33.7-57.61) | 21078 (17789-27732) | 37.29 (31.58-48.89) | -0.87 (-1.11--0.62) |
| South Sudan | 2614 (1309-3797) | 55.94 (28.78-84.15) | 3396 (1709-5125) | 44.67 (23.66-68.96) | -0.94 (-1.3--0.58) |
| Spain | 7491 (6958-7912) | 15.33 (14.31-16.12) | 31766 (27452-34728) | 32.55 (29.25-35.13) | 2.99 (2.05-3.94) |
| Sri Lanka | 6060 (4813-8229) | 41.63 (33.25-57.05) | 7994 (5295-11369) | 32.91 (22.27-46.7) | -0.29 (-0.51--0.06) |
| Sudan | 6991 (4476-10803) | 31.27 (21.64-45.29) | 7760 (5135-11048) | 20.56 (14.03-29.44) | -1.28 (-1.38--1.19) |
| Suriname | 56 (45-74) | 16.28 (12.97-22.18) | 92 (63-121) | 15.21 (10.31-20.05) | 0.03 (-0.11-0.18) |
| Sweden | 2352 (2101-2648) | 16.88 (15.17-19.21) | 3289 (2856-3645) | 15.21 (13.48-16.81) | 0.23 (0-0.46) |
| Switzerland | 2213 (2007-2451) | 23.52 (21.48-26.05) | 11483 (9574-12711) | 59.01 (51.3-64.23) | 4.3 (3.48-5.12) |
| Syrian Arab Republic | 1140 (764-1676) | 9.5 (6.54-13.38) | 724 (490-979) | 5.45 (3.8-7.31) | -1.99 (-2.26--1.73) |
| Taiwan (Province of China) | 2106 (2009-2197) | 11.84 (11.33-12.32) | 17772 (15952-19240) | 50.38 (45.91-54.05) | 6.85 (5.84-7.87) |
| Tajikistan | 83 (48-134) | 1.35 (0.9-2.02) | 139 (92-204) | 1.31 (0.89-1.86) | -0.28 (-0.5--0.07) |
| Thailand | 54085 (43555-68567) | 113.04 (90.71-144.18) | 65797 (48830-90728) | 77.83 (59.13-105.63) | -1.36 (-1.49--1.23) |
| Timor-Leste | 251 (167-408) | 43.02 (26.21-72.76) | 669 (365-1206) | 57.96 (30.63-106.58) | 1.46 (1.24-1.68) |
| Togo | 1204 (633-1771) | 51.43 (28.36-78.41) | 2718 (1382-4227) | 44.64 (23.61-68.82) | -0.5 (-0.57--0.43) |
| Tokelau | 1 (1-2) | 74.92 (46.08-129.49) | 1 (1-2) | 99.95 (76.93-135.08) | 0.01 (-0.36-0.39) |
| Tonga | 30 (20-52) | 42.64 (28.4-76.96) | 40 (28-58) | 44.03 (30.89-64.32) | 0.36 (0.26-0.45) |
| Trinidad and Tobago | 384 (360-409) | 36.11 (33.87-38.39) | 666 (508-858) | 41.58 (31.67-53.38) | 0.4 (0.22-0.57) |
| Tunisia | 1631 (1217-2267) | 21.29 (16.05-30.73) | 2146 (1312-3505) | 17.06 (10.52-27.48) | -0.47 (-0.7--0.24) |
| Turkey | 27506 (18757-38796) | 55.05 (38.86-73.94) | 20256 (15963-26939) | 23.35 (18.5-30.82) | -2.78 (-2.83--2.72) |
| Turkmenistan | 275 (183-457) | 7.37 (5.39-11.14) | 522 (371-779) | 10.35 (7.37-15.3) | 0.94 (0.68-1.21) |
| Tuvalu | 7 (5-11) | 89.2 (59.17-147.91) | 9 (7-12) | 80.16 (59.73-111.32) | -0.16 (-0.24--0.07) |
| Uganda | 7683 (4108-11773) | 57.56 (32.54-93.06) | 12242 (7512-18426) | 38.59 (22.54-58.48) | -1.67 (-1.8--1.53) |
| Ukraine | 8721 (7585-10094) | 14.87 (12.84-17.31) | 12017 (8745-15869) | 23.13 (17.06-30.45) | 1.25 (1.03-1.48) |
| United Arab Emirates | 364 (240-557) | 30.72 (20.2-44.47) | 1234 (831-1876) | 19.07 (14.71-25.36) | -0.26 (-0.62-0.11) |
| United Kingdom | 5925 (5731-6047) | 7.47 (7.26-7.61) | 33896 (31024-35398) | 28.55 (26.72-29.64) | 5.52 (4.57-6.49) |
| United Republic of Tanzania | 11999 (6940-16816) | 62.35 (36.98-86.49) | 19272 (12347-28116) | 42.99 (28.13-63.88) | -1.33 (-1.4--1.27) |
| United States of America | 117779 (109576-126117) | 38.77 (36.1-41.59) | 194858 (179626-206136) | 39.85 (37.29-41.99) | 0.1 (0.01-0.2) |
| United States Virgin Islands | 47 (35-68) | 46.87 (35.05-68.14) | 34 (24-44) | 35.05 (24.93-46.92) | -0.21 (-0.43-0.01) |
| Uruguay | 2173 (2032-2303) | 60.75 (56.94-64.32) | 3286 (3032-3525) | 65.95 (61.23-70.75) | 0.34 (0.22-0.46) |
| Uzbekistan | 2310 (1685-2979) | 9.89 (7.55-12.33) | 1945 (1567-2371) | 5.75 (4.62-7) | -1.7 (-2.13--1.27) |
| Vanuatu | 60 (38-101) | 58.07 (35.88-101.04) | 183 (130-271) | 72.2 (51.86-107.15) | 0.68 (0.59-0.76) |
| Venezuela (Bolivarian Republic of) | 1597 (1444-1736) | 9.72 (8.85-10.55) | 3997 (3026-5159) | 14.44 (11.04-18.44) | 1.16 (0.83-1.5) |
| Viet Nam | 18601 (11684-31705) | 35.8 (21.86-64.32) | 25476 (14696-46900) | 24.67 (14.28-45.77) | -1.27 (-1.32--1.22) |
| Yemen | 3982 (2419-6095) | 28.37 (19.64-39.76) | 6585 (4500-9231) | 25.16 (17.57-35.93) | -0.49 (-0.57--0.41) |
| Zambia | 3308 (1781-4612) | 57.51 (32.86-78.81) | 5426 (3304-7845) | 41.33 (24.56-58.34) | -1.25 (-1.39--1.11) |
| Zimbabwe | 1234 (939-1765) | 17.37 (13.12-25.2) | 3438 (2328-4999) | 26.39 (18.09-37.76) | 1.92 (1.58-2.26) |
