# Supplementary material for: Analysis of the International, Regional, and National Endocarditis-Related Disease Burdens (1990–2021), and Changes to Projections for the Next 15 Years: A Population-Based Study
Source: Rev Cardiovasc Med. 2025 May 20;26(5):27168. doi: 10.31083/RCM27168 (PMC12135659; doi:10.31083/RCM27168)
Supplement: Supplementary file 1 [file 2153-8174-26-5-27168-s1.zip › Supplementary Figures.docx]

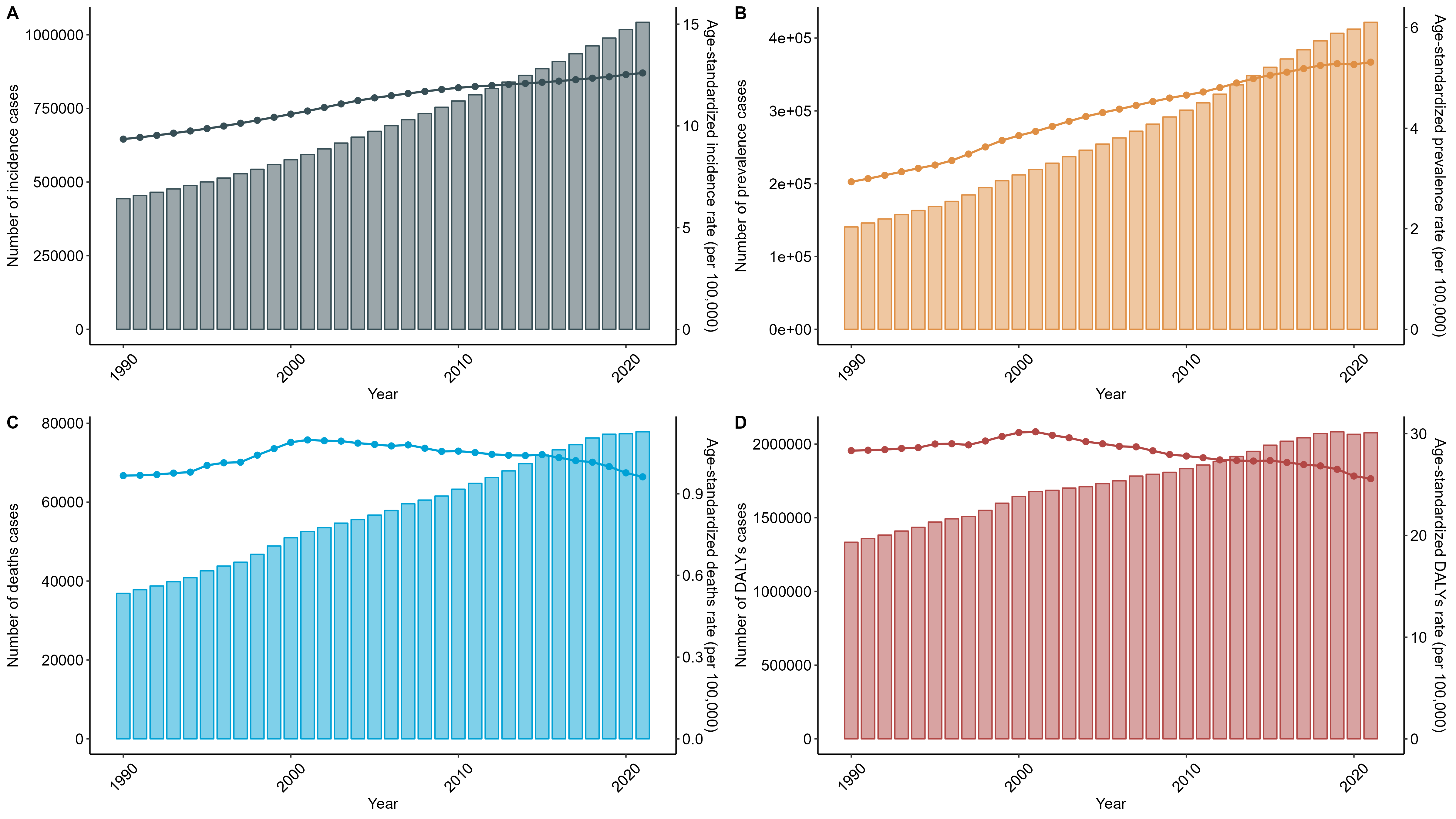


**Supplement Figure 1** Time trends in the absolute numbers and age-standardized rates (ASR) of incidence, prevalence, deaths, and disability-adjusted life years (DALYs) due to endocarditis from 1990 to 2021. Panel A (Gray): Shows the annual number of new incidence cases (left Y-axis, absolute count) and age-standardized incidence rate (ASR, right Y-axis, per 100,000 population). The absolute number of cases increases steadily over time, while ASR remains relatively stable with a slight upward trend. Panel B (Orange): Illustrates the annual number of prevalence cases (left Y-axis, absolute count) and the age-standardized prevalence rate (ASR, right Y-axis, per 100,000 population). Both absolute counts and ASR show significant growth during the observed period. Panel C (Blue): Displays the annual number of deaths (left Y-axis, absolute count) and the age-standardized death rate (ASR, right Y-axis, per 100,000 population). Deaths have steadily increased, while ASR remains relatively stable or slightly declining, potentially reflecting improvements in medical care. Panel D (Red): Represents the annual number of disability-adjusted life years (DALYs, left Y-axis, absolute count) and age-standardized DALY rate (ASR, right Y-axis, per 100,000 population). DALYs increase over time, but the ASR shows a slight downward trend, suggesting that the burden of disease might be stabilizing when standardized by age.


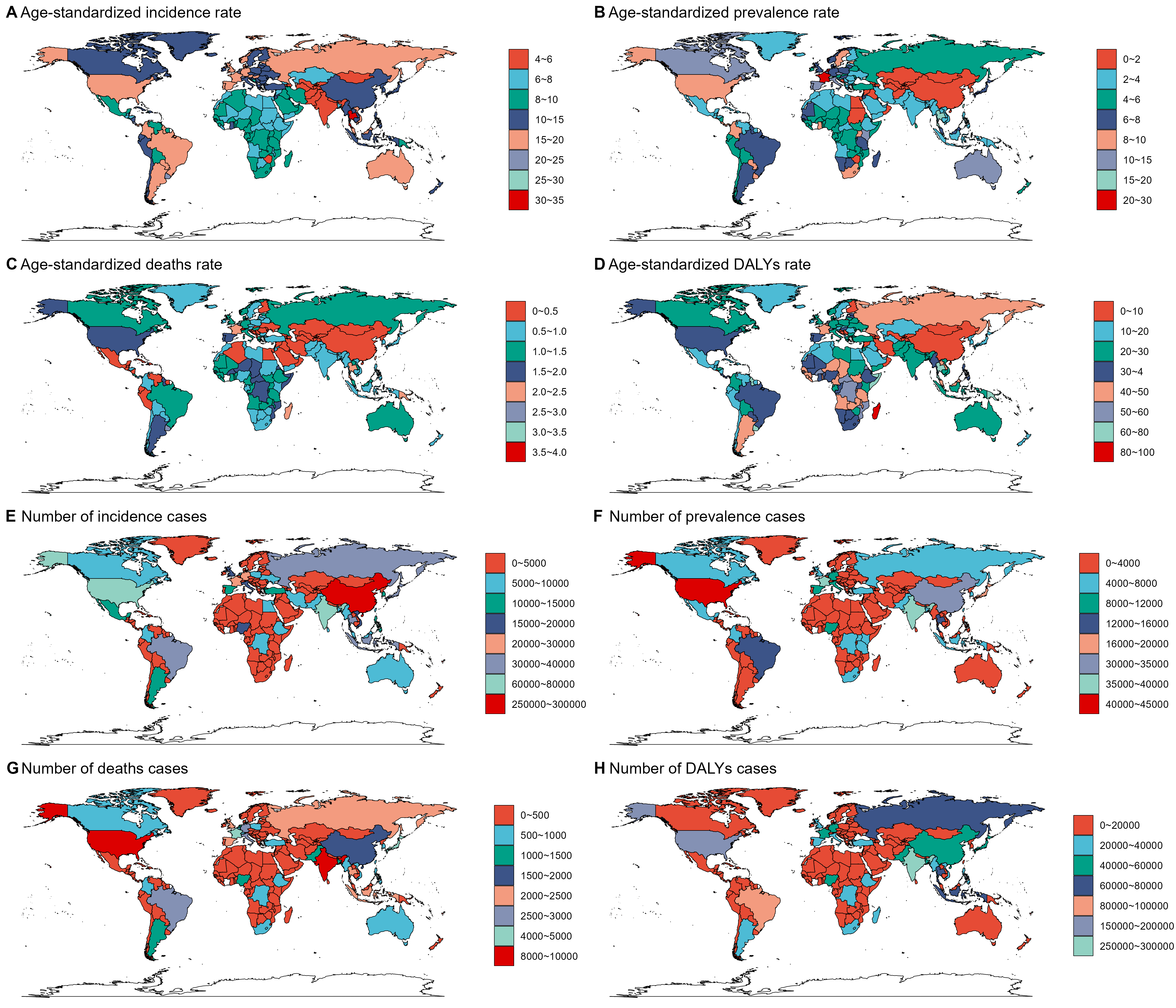


**Supplement Figure 2** Distribution of ASR and Absolute Numbers of Incidence, Prevalence, Deaths, and DALYs in 204 Countries and Territories Worldwide in 2021. Panels A-D: Depict the distribution of age-standardized rates (ASR) across countries, including incidence, prevalence, death rates, and disability-adjusted life years (DALYs). The gradient from blue to red represents different ranges of values (see legend on the right). Panel A: Age-standardized incidence rate (ASIR). Higher ASIR is observed in regions such as the Thailand. Panel B: Age-standardized prevalence rate (ASPR). Higher ASPR is concentrated in North Africa, the Middle East, and some Southeast Asian countries.Panel C: Age-standardized death rate (ASDR). The highest ASDR is found in China etc. , while other regions have relatively lower rates.Panel D: Age-standardized DALY rate. China, etc. exhibit the highest burden of health loss, while developed regions such as North America and Western Europe show lower rates.Panels E-H: Show the global distribution of absolute numbers of cases, including incidence, prevalence, deaths, and DALYs. The gradient from blue to red represents the range of total case counts.Panel E: Number of incidence cases. The highest counts are observed in densely populated regions such as South Asia (e.g., India), China, and Sub-Saharan Africa. Panel F: Number of prevalence cases. the United States, the Middle East, and Sub-Saharan Africa show the highest prevalence numbers, while North America and Europe have relatively lower counts.Panel G: Number of death cases. The highest death tolls are observed in the United States, with lower numbers in developed countries. Panel H: Number of DALYs. The burden of DALYs is concentrated in India.


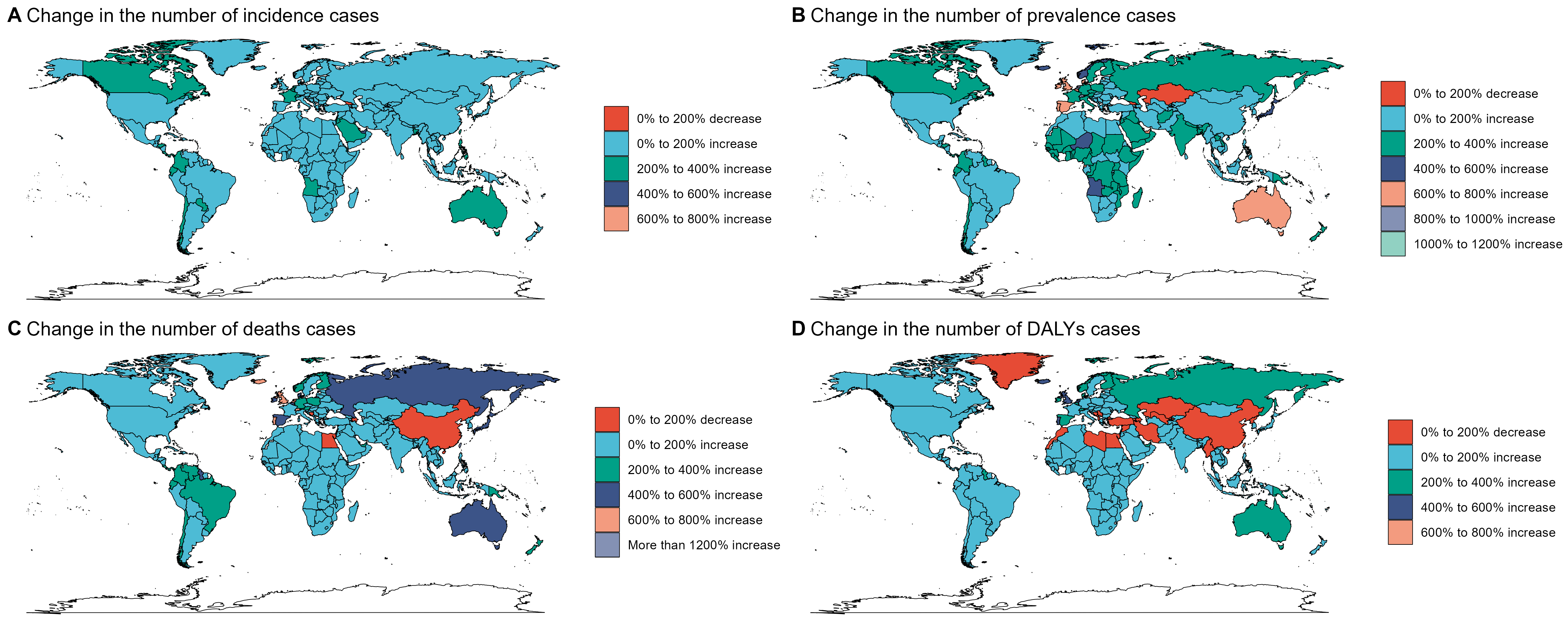


**Supplement Figure 3** Distribution of Changes in the Number of Incidence (A), Prevalence (B), Deaths (C), and DALYs (D) of Endocarditis in 204 Countries and Territories Worldwide, 1990–2021. Panels A-D: Present the percentage changes in the number of incidence, prevalence, deaths, and DALYs of endocarditis between 1990 and 2021 across 204 countries and territories. The color gradient represents different percentage change ranges (see the legend on the right), reflecting the trends and dynamic shifts in disease burden across regions:Panel A: Change in the number of incidence cases. Most countries experienced an increase in incidence cases within the range of 0%-200%. In countries such as Canada and Australia, the increase was between 200%-400%.Panel B: Change in the number of prevalence cases. Prevalence has grown significantly globally, with most countries showing an increase of 200%-400%, reflecting the accumulation of chronic cases and the impact of aging populations.Panel C: Change in the number of deaths. The change in deaths varies greatly across regions, with some countries showing significant increases in death counts, while China shows a declining trend.

Panel D: Change in the number of DALYs. DALYs (disability-adjusted life years) have significantly increased in many regions, while some countries have shown a decrease. This figure comprehensively illustrates the dynamic changes in endocarditis burden across regions, emphasizing the growing challenges of disease burden in resource-limited areas.


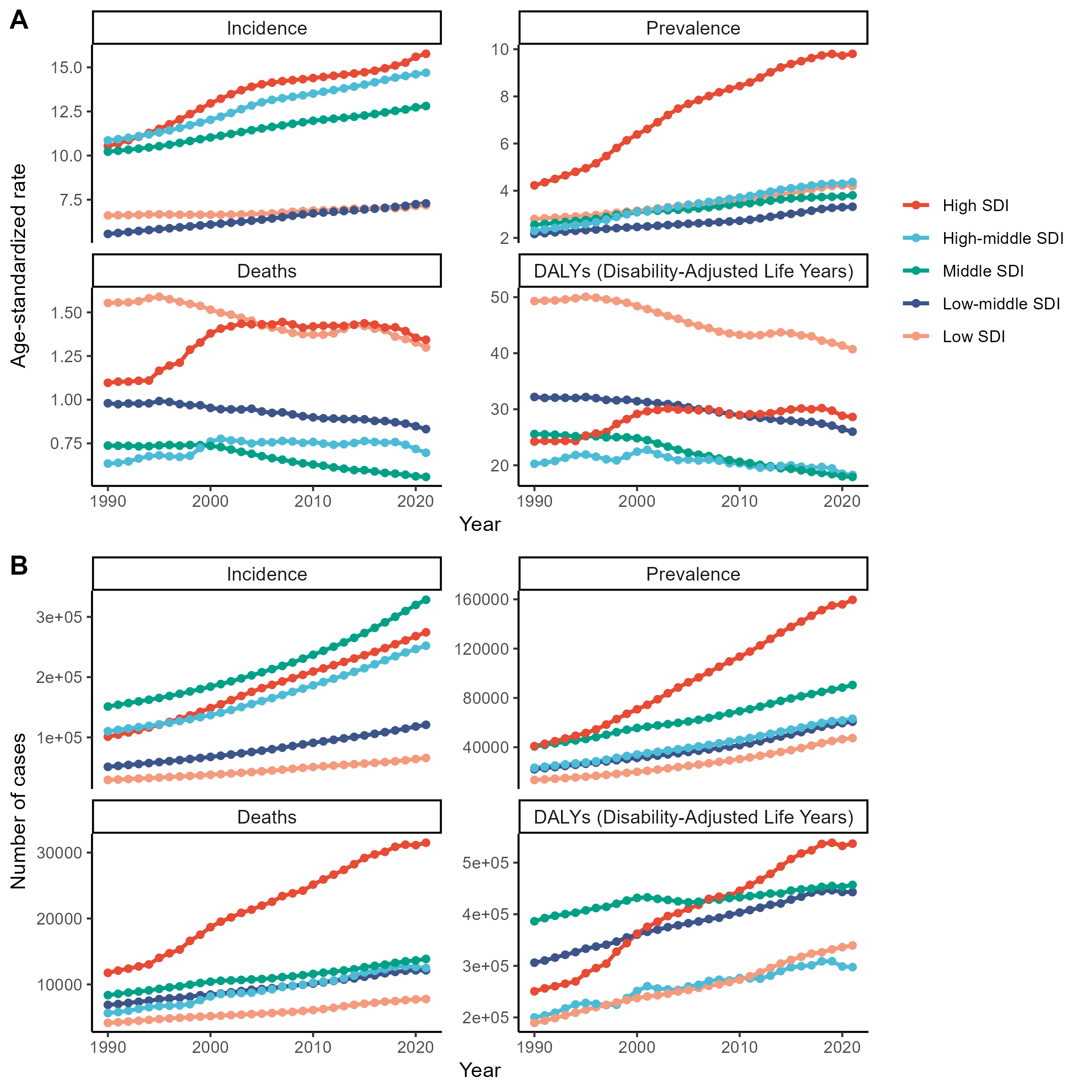


**Supplement Figure 4** Trends in the Temporal Distribution of ASR and the Number of Cases of Endocarditis in the GBD Regions by SDI Quintile, 1990–2021.**Panel A:** Shows the age-standardized rates (ASR) of endocarditis from 1990 to 2021, stratified by SDI (Socio-Demographic Index) quintiles, including incidence, prevalence, deaths, and disability-adjusted life years (DALYs).Incidence rate: All SDI groups show an increasing trend, with high-SDI countries consistently having the highest incidence rates. The low-middle SDI group shows the fastest growth.Prevalence rate: Prevalence rates have risen steadily across all groups, with high-SDI countries demonstrating significantly higher rates than other groups, reflecting the accumulation of chronic cases.Death rate: Death rates in high and high-middle SDI groups have gradually declined after 2000, while low-SDI countries show minimal changes with a slight downward trend.DALYs: The DALY burden in low-SDI countries shows a significant disparity compared to other regions.**Panel B:** Depicts the total number of cases of endocarditis from 1990 to 2021 in each SDI quintile, including incidence, prevalence, deaths, and DALYs.Incidence cases: The number of incidence cases has increased significantly across all SDI groups, with high-SDI countries experiencing the fastest growth.Prevalence cases: Prevalence cases are highest and growing fastest in high-SDI countries.Death cases: High-SDI countries have the highest number of death cases, while low-SDI countries have fewer cases and slower growth.DALYs cases: The burden of DALYs has increased across all SDI groups.


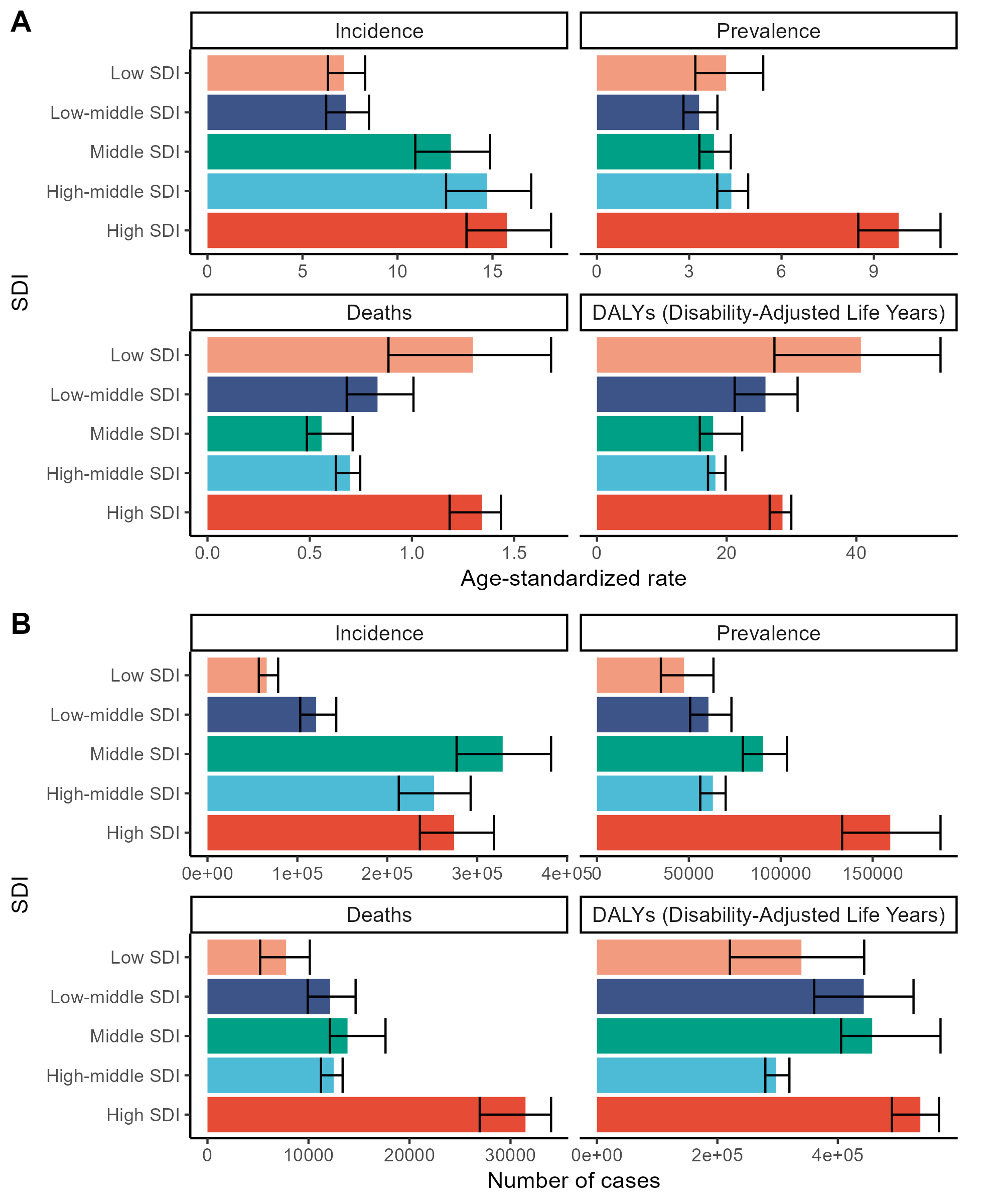


**Supplement Figure 5** The age-standardized rates (ASR) and total number of cases of incidence, prevalence, deaths, and DALYs of endocarditis in the Global Burden of Disease (GBD) regions stratified by SDI (Socio-Demographic Index) quintiles in 2021.**Panel A:** Displays the age-standardized rates (ASR) for different SDI quintiles, including incidence, prevalence, deaths, and disability-adjusted life years (DALYs).Incidence: The incidence rate is highest in high-SDI regions, followed by other SDI groups.Prevalence: The prevalence rate is significantly higher in high-SDI regions, reflecting the chronic accumulation of endocarditis cases.Deaths: Death rates are higher in both low-SDI and high-SDI regions.DALYs: The DALY burden is most pronounced in low-SDI regions, highlighting the vulnerability of resource-limited areas to the disease burden.**Panel B:** Displays the total number of cases of endocarditis in different SDI quintiles, including incidence, prevalence, deaths, and DALYs.Incidence cases: The number of cases is highest in high-SDI and middle-SDI groups.Prevalence cases: The prevalence cases are highest in high-SDI regions.Death cases: The number of deaths remains relatively high in high-SDI regions.DALY cases: High-SDI regions account for a large proportion of DALY cases.


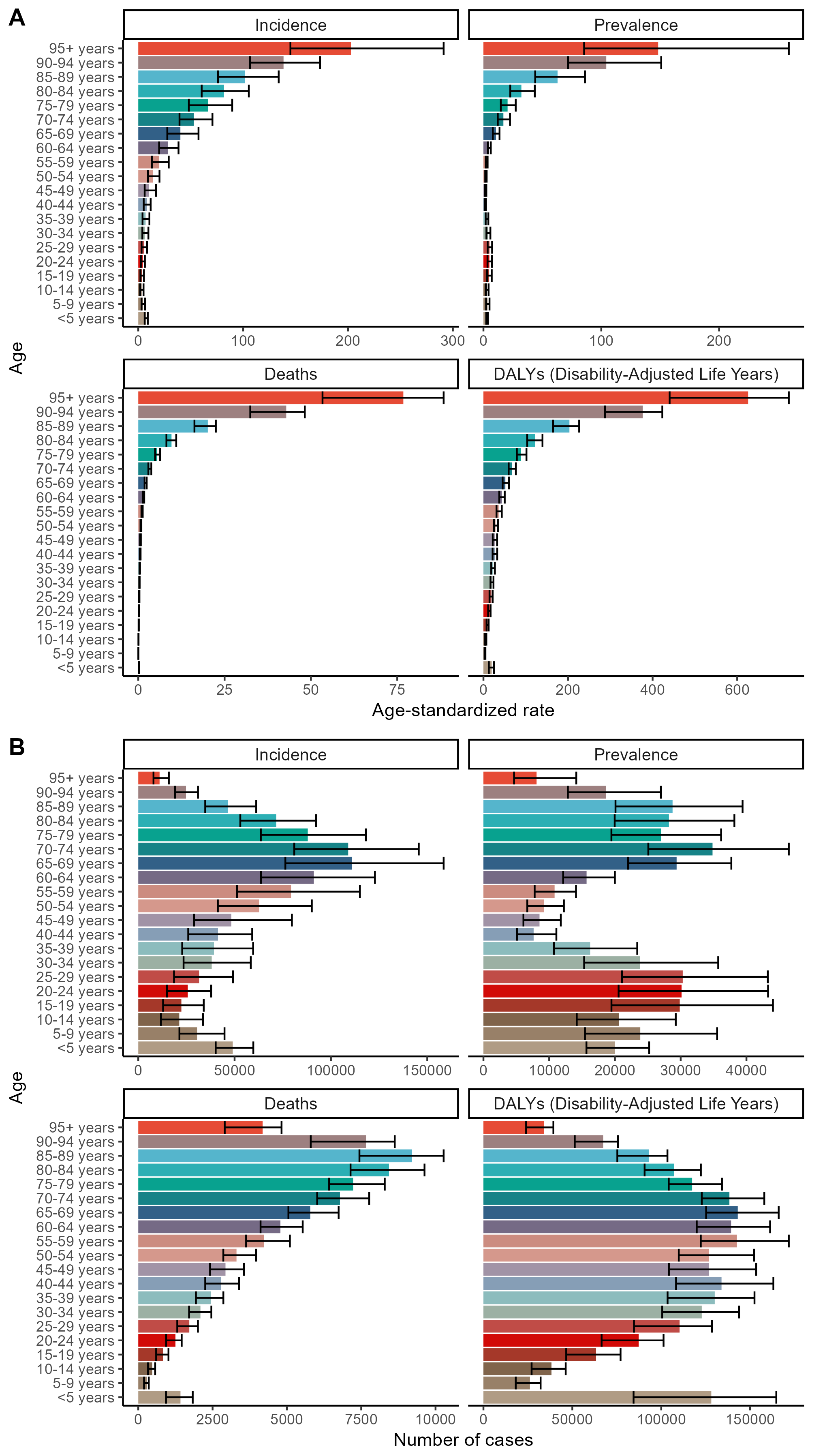


**Supplement Figure 6** Age-standardized rates (ASR, Panel A) and total number of cases (Panel B) of incidence, prevalence, deaths, and disability-adjusted life years (DALYs) of endocarditis by age group in 2021.**Panel A:** Displays the age-standardized rates (ASR) for different age groups, including incidence, prevalence, deaths, and DALYs.Incidence: The incidence rate increases significantly with age, with the highest rate observed in individuals aged 95 years and older.Prevalence: Prevalence rates rise sharply in older age groups, reflecting the accumulation of chronic cases.Deaths: Death rates increase with age, with a significant rise in individuals aged 85 years and older.DALYs: The burden of DALYs is primarily concentrated in people aged 65 years and older, highlighting the substantial impact of the disease on healthy life expectancy in the elderly.**Panel B:** Displays the total number of cases of endocarditis by age group, including incidence, prevalence, deaths, and DALYs.Incidence cases: The number of incidence cases peaks among individuals aged 65-74 years.Prevalence cases: Prevalence cases are notably higher among individuals aged 15-29 years and those aged 65 years and older.Death cases: Death cases are predominantly concentrated in older age groups.DALY cases: The DALY burden is mainly concentrated in middle-aged and elderly populations, underscoring the vulnerability of these age groups to the disease burden.


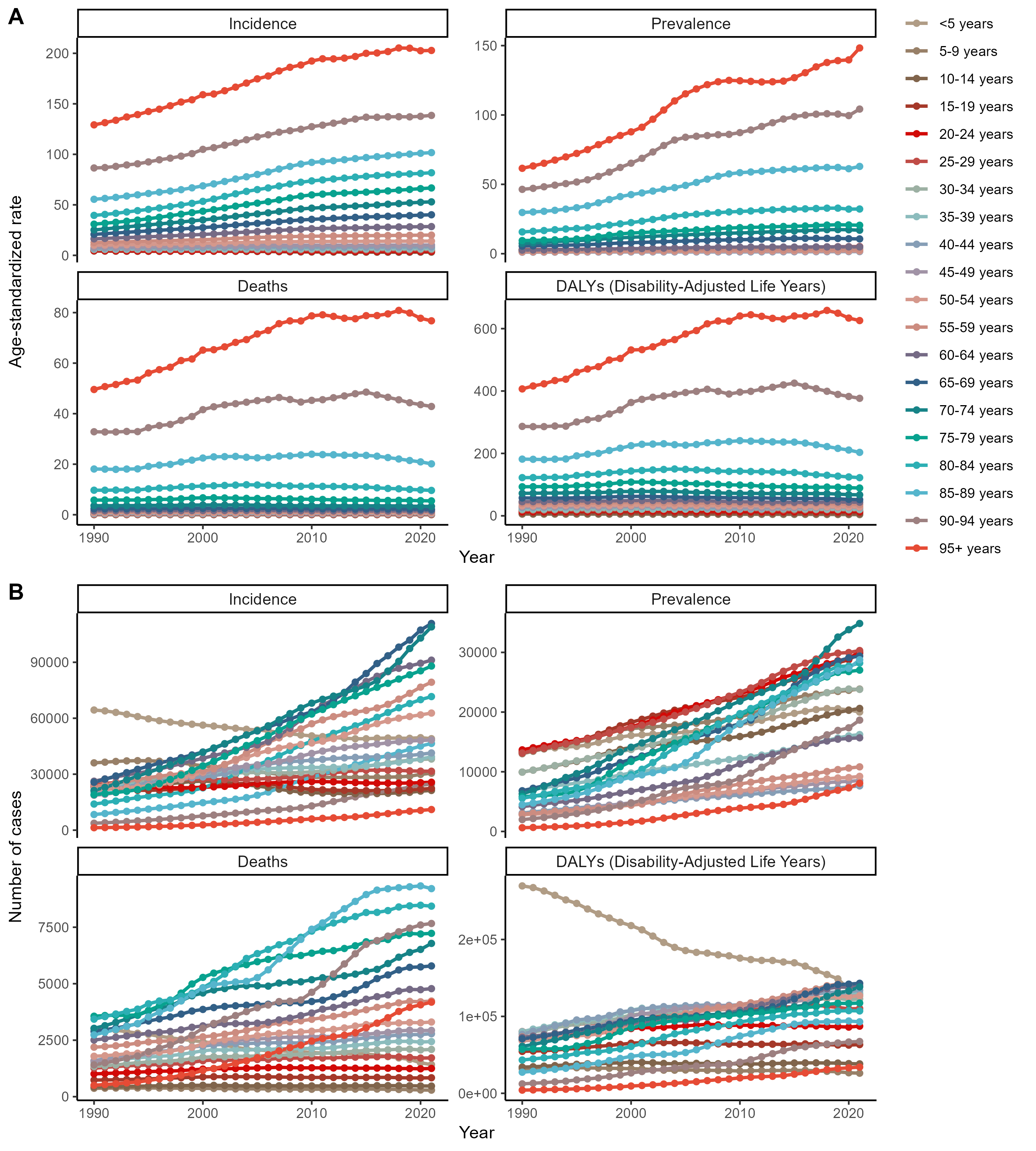


**Supplement Figure 7** Trends in the temporal distribution of age-standardized rates (ASR, Panel A) and total number of cases (Panel B) of incidence, prevalence, deaths, and disability-adjusted life years (DALYs) of endocarditis by age group from 1990 to 2021.**Panel A:** Shows the trends in age-standardized rates (ASR) for different age groups from 1990 to 2021, including incidence, prevalence, deaths, and DALYs.Incidence: Incidence rates have steadily increased across all age groups, with the highest rates consistently observed in individuals aged 95 years and older. Rates in children remain low with minimal changes over time.Prevalence: Prevalence rates have increased annually across all age groups, with older populations (especially those aged 95+ years) showing the most significant growth.Deaths: Death rates rise with age, with a pronounced increase in older adults (90-94 years and 95+ years), while younger age groups exhibit low and relatively stable rates.DALYs: The DALY burden has increased steadily across all age groups, with the highest burden observed in the 95+ years age group.

**Panel B:** Illustrates the trends in the total number of cases for incidence, prevalence, deaths, and DALYs by age group from 1990 to 2021.Incidence cases: The number of incidence cases has shown a consistent upward trend across most age groups.Prevalence cases: Prevalence cases have significantly increased in older populations, reflecting the cumulative effect of chronic disease.Death cases: Death cases have risen sharply in older age groups (75 years and above), while younger age groups maintain consistently lower numbers.DALY cases: The number of DALY cases has increased across most age groups, with a notable decline observed in the 0-5 years age group.


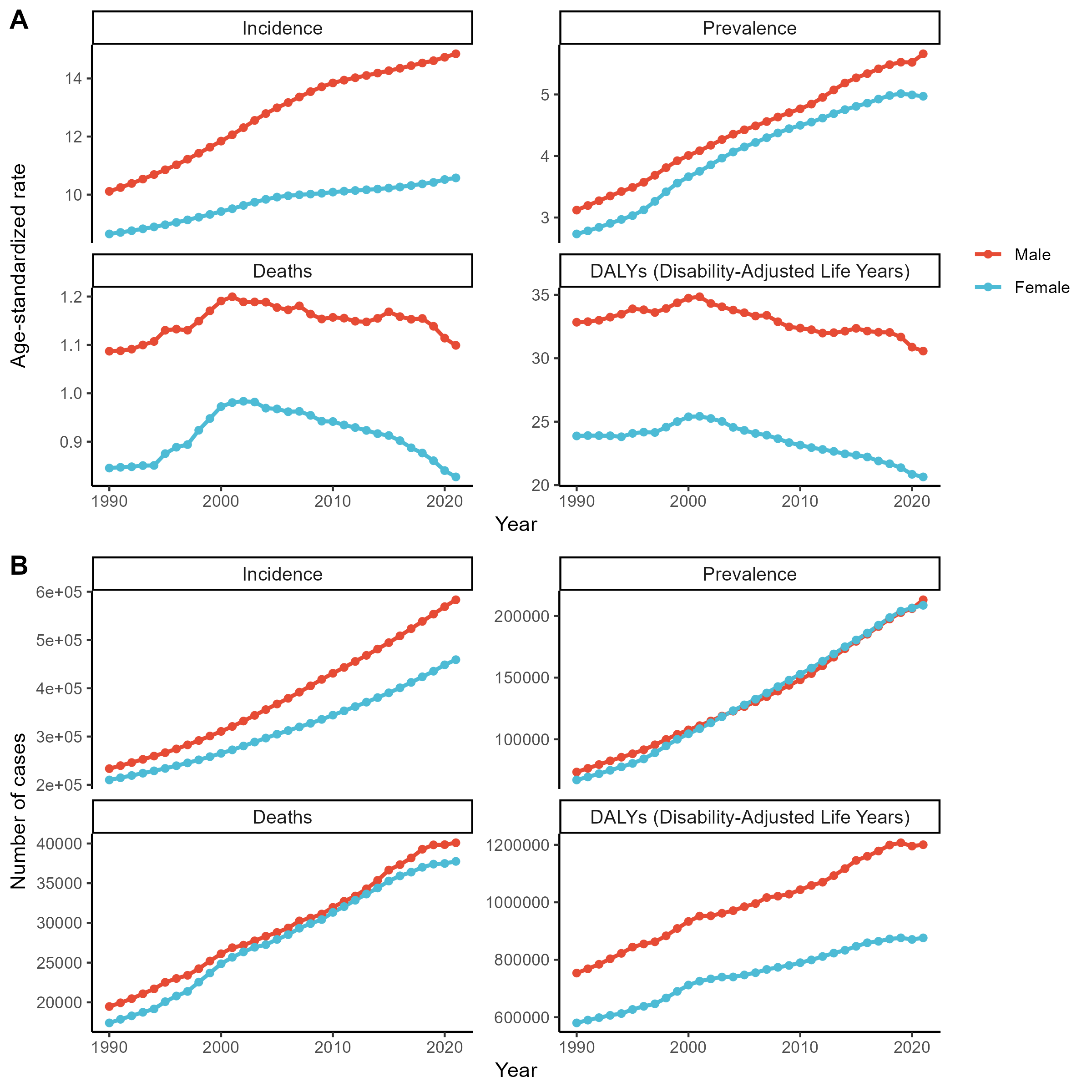


**Supplement Figure 8** Trends in the temporal distribution of age-standardized rates (ASR, Panel A) and total number of cases (Panel B) of incidence, prevalence, deaths, and disability-adjusted life years (DALYs) of endocarditis by sex from 1990 to 2021.**Panel A:** Displays the trends in age-standardized rates (ASR) by sex from 1990 to 2021, including incidence, prevalence, deaths, and DALYs.Incidence: The incidence rate in males is significantly higher than in females, with a steady increase over time and a widening gap between the two sexes.Prevalence: Prevalence rates are higher in males, and both sexes exhibit an upward trend over time.Deaths: Death rates in males peaked around 2000 and subsequently showed a fluctuating decline, whereas female death rates increased slightly before 2000 and then steadily decreased.DALYs: The DALY burden in males is consistently higher than in females. Female DALY burden remains relatively low and has shown a gradual decline since 2000.**Panel B:** Illustrates the trends in total cases of incidence, prevalence, deaths, and DALYs by sex from 1990 to 2021.Incidence cases: The number of incidence cases has increased steadily in both sexes, with males consistently showing higher numbers.Prevalence cases: The prevalence cases show a rising trend for both males and females.Death cases: Death cases have increased gradually in both sexes, with a relatively small difference, though males exhibit slightly higher numbers.DALY cases: The number of DALY cases in males is significantly higher than in females, with a steady increase over time.


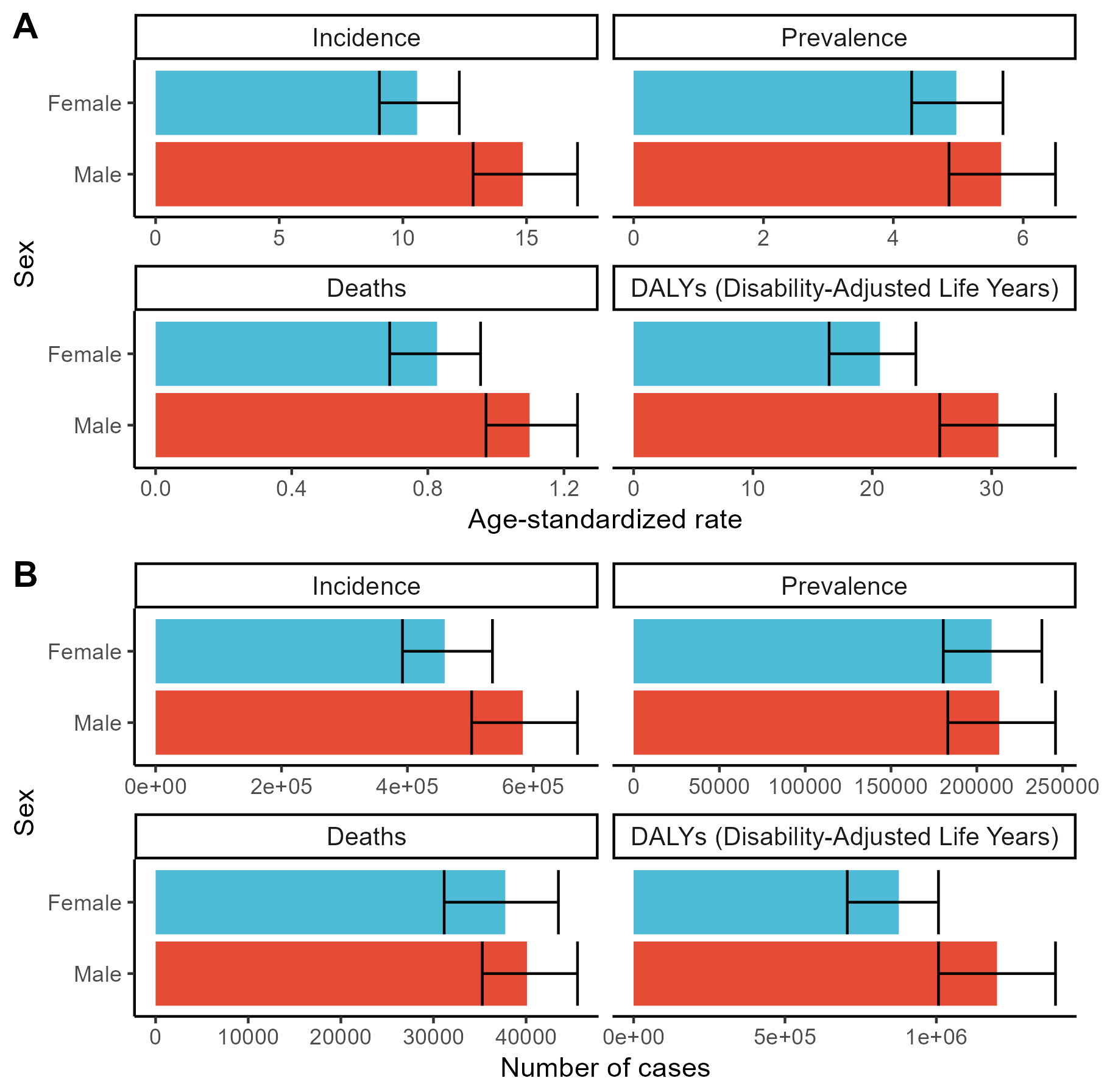


**Supplement Figure 9** The global burden of endocarditis in 2021 by sex: age-standardized rates (ASR, Panel A) and total number of cases (Panel B) of incidence, prevalence, deaths, and disability-adjusted life years (DALYs).**Panel A:** Displays the age-standardized rates (ASR) for males and females, including incidence, prevalence, deaths, and DALYs.Incidence: The incidence rate in males is significantly higher than in females.Prevalence: The prevalence rate is higher in males, with a noticeable gap between the two sexes.Deaths: The death rate in males is higher than in females, with a substantial difference between the two groups.DALYs: The DALY burden is significantly higher in males, reflecting a more considerable impact on healthy life years compared to females.**Panel B:** Shows the total number of cases of endocarditis by sex in 2021, including incidence, prevalence, deaths, and DALYs.Incidence cases: The number of incidence cases is noticeably higher in males than in females.Prevalence cases: Both males and females show high prevalence, with relatively minor differences between the two.Death cases: The number of deaths is higher in males than in females.DALY cases: The number of DALY cases in males is significantly higher than in females, highlighting the greater disease burden among males.


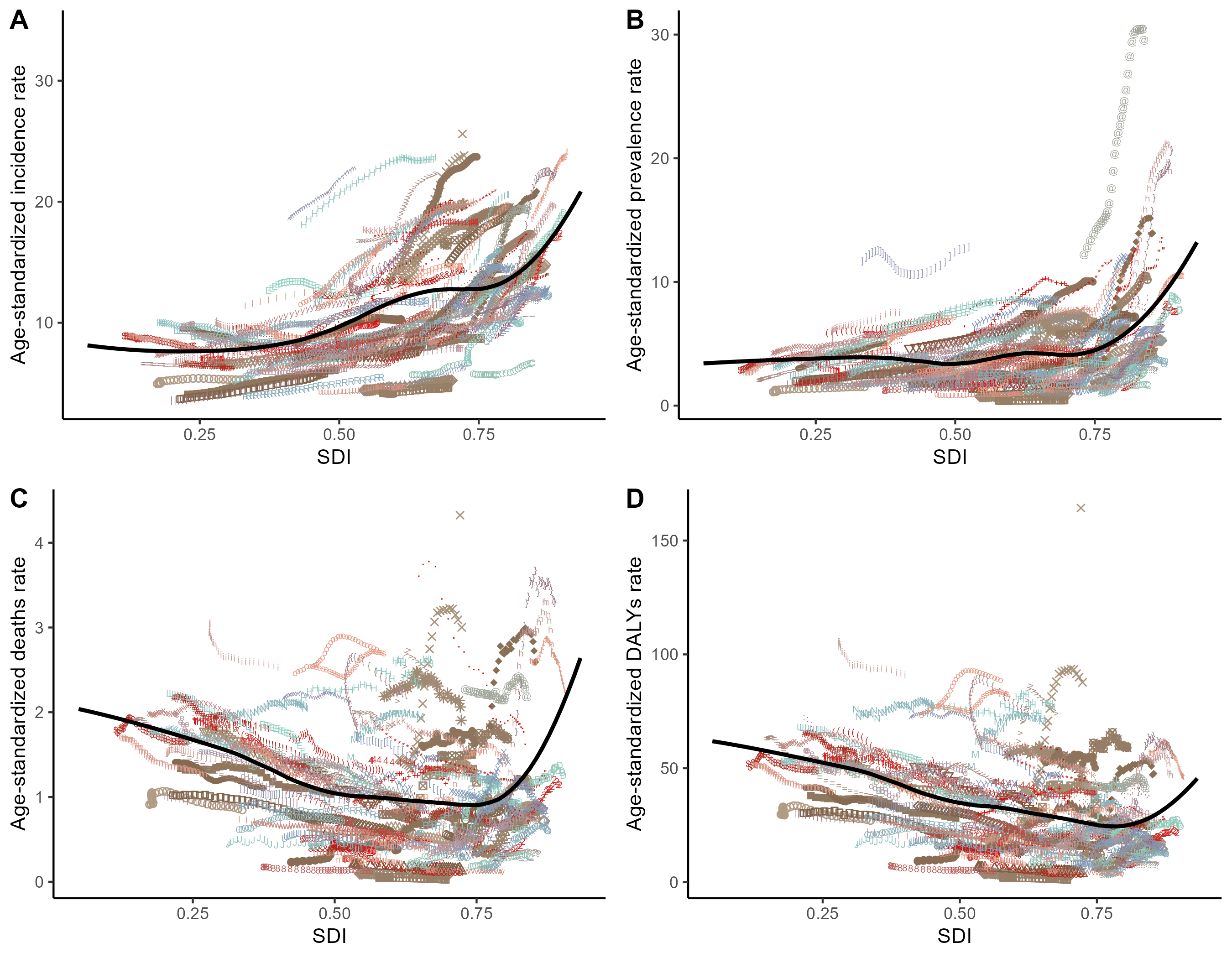


**Supplement Figure 10** Analysis of the correlation between incidence, prevalence, deaths, and disability-adjusted life years (DALYs) of endocarditis and sociodemographic index (SDI) levels in Global Burden of Disease (GBD) regions from 1990 to 2021.Panel A: Shows the relationship between age-standardized incidence rates and SDI levels. Incidence rates increase with rising SDI levels, with a particularly notable upward trend in high-SDI regions.Panel B: Displays the relationship between age-standardized prevalence rates and SDI levels. Prevalence rates show a more pronounced increase in high-SDI regions.Panel C: Illustrates the relationship between age-standardized death rates and SDI levels. Death rates are higher in low-SDI regions and decline as SDI levels improve, but they show a slight rebound in high-SDI regions.Panel D: Depicts the relationship between age-standardized DALY rates and SDI levels. DALY rates are higher in low-SDI regions and gradually decline as SDI levels increase, but there is a slight rise in high-SDI regions, indicating a dual burden of disease.


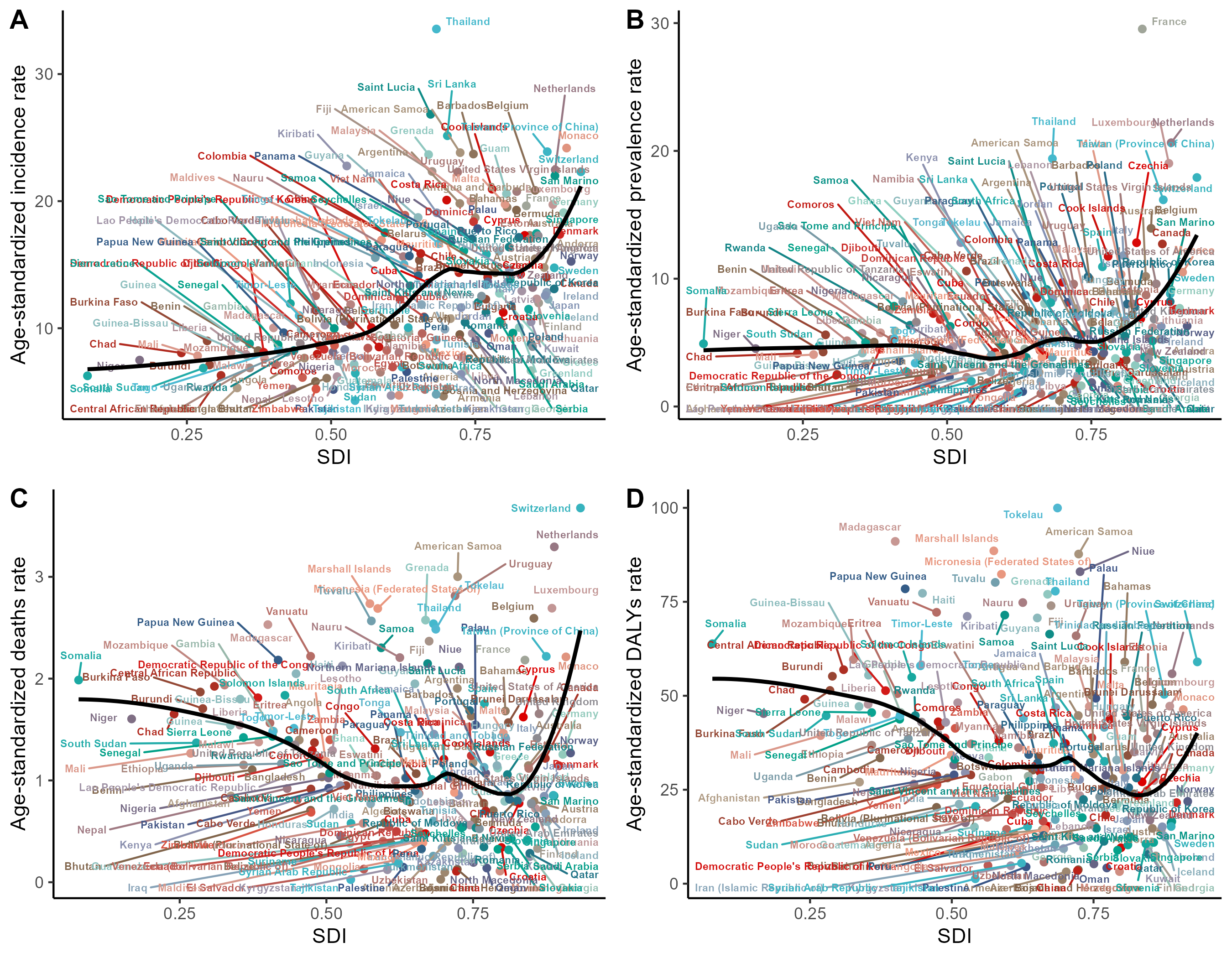


**Supplement Figure 11** Legend: Analysis of the correlation between age-standardized rates (ASR) and sociodemographic index (SDI) levels for incidence, prevalence, deaths, and disability-adjusted life years (DALYs) of endocarditis in 204 countries and regions worldwide in 2021.Panel A: Displays the relationship between age-standardized incidence rates and SDI levels. Incidence rates are relatively low in low-SDI regions but increase progressively as SDI levels rise, with a notable upward trend in high-SDI regions. Certain countries, such as Thailand, exhibit particularly high incidence rates.Panel B: Illustrates the relationship between age-standardized prevalence rates and SDI levels. Prevalence rates are lower in countries with low SDI but increase significantly with rising SDI, particularly in high-SDI regions. France, for instance, shows higher prevalence rates.Panel C: Shows the relationship between age-standardized death rates and SDI levels. Death rates are higher in low-SDI regions and decline as SDI levels improve but show a slight rebound in high-SDI regions. Countries like Chad and Niger have particularly high death rates among low-SDI countries.Panel D: Depicts the relationship between age-standardized DALY rates and SDI levels. DALY rates are elevated in low-SDI regions and decrease as SDI levels rise but display a slight rebound in high-SDI countries, indicating the complex nature of the disease burden. Countries like Tokelau and the Marshall Islands exhibit higher DALY rates.
